# Spin Dynamics of Radical Pairs Using the Stochastic Schrödinger Equation in *MolSpin*

**DOI:** 10.1021/acs.jctc.4c00361

**Published:** 2024-09-16

**Authors:** Gediminas Jurgis Pažėra, Thomas P. Fay, Ilia A. Solov’yov, P. J. Hore, Luca Gerhards

**Affiliations:** † Department of Chemistry, University of Oxford, Physical and Theoretical Chemistry Laboratory, Oxford OX1 3QZ, United Kingdom; ‡ Department of Chemistry, University of California, Berkeley, California 94720, United States; § Institute of Physics, Carl von Ossietzky Universität Oldenburg, Carl-von-Ossietzky Str. 9-11, Oldenburg 26129, Germany; ∥ Research Center for Neurosensory Science, Carl von Ossietzky Universität Oldenburg, Oldenburg 26111, Germany; ⊥ Center for Nanoscale Dynamics (CENAD), Carl von Ossietzky Universität Oldenburg, Institut für Physik, Ammerländer Heerstr. 114-118, Oldenburg 26129, Germany

## Abstract

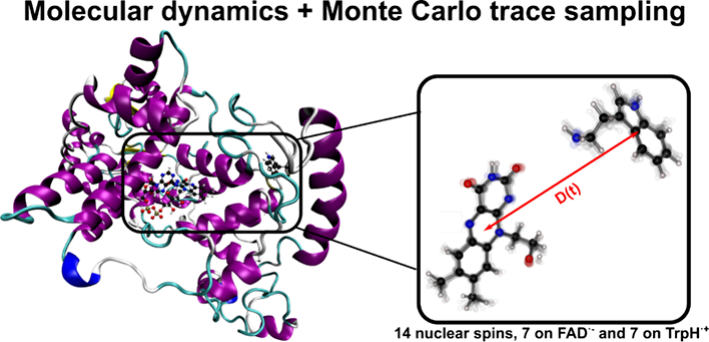

The chemical reactivity of radical pairs is strongly influenced by the interactions of electronic and nuclear spins. A detailed understanding of these effects requires a quantum description of the spin dynamics that considers spin-dependent reaction rates, interactions with external magnetic fields, spin–spin interactions, and the loss of spin coherence caused by coupling to a fluctuating environment. Modeling real chemical and biochemical systems, which frequently involve radicals with multinuclear spin systems, poses a severe computational challenge. Here, we implement a method based on the stochastic Schrödinger equation in the software package *MolSpin*. Large electron–nuclear spin systems can be simulated efficiently, with asymmetric spin-selective recombination reactions, anisotropic hyperfine interactions, and nonzero exchange and dipolar couplings. Spin-relaxation can be modeled using the stochastic time-dependence of spin interactions determined by molecular dynamics and quantum chemical calculations or by allowing rate coefficients to be explicitly time-dependent. The flexibility afforded by this approach opens new avenues for exploring the effects of complex molecular motions on the spin dynamics of chemical transformations.

## Introduction

Radical pairs are short-lived reaction intermediates whose chemistry is primarily governed by weak spin-dependent interactions and the requirement that reactions should conserve electron spin.^1^ The radical pair mechanism (RPM), part of the larger field of spin chemistry,^2^ has seen increased attention lately mainly due to its possible involvement in the magnetic compass sensor of migratory birds^3−8^ and in lipid peroxidation.^9^ Often formed photochemically in spin-correlated electronic singlet or triplet states, radical pairs are sensitive to weak external magnetic fields, which can modify their coherent spin dynamics and so change the yields of their reaction products.^10,11^ These effects are conditioned by internal magnetic interactions, in particular those of the electrons with magnetic nuclei (hyperfine interactions).^12^ An accurate quantum mechanical description of radical pair reactivity becomes challenging when, as is often the case for biologically relevant radicals, a large number of hyperfine interactions must be included and molecular motions, which induce spin relaxation, cannot be ignored.^13−15^

In 2016, Lewis et al.^16^ pioneered a technique for simulating the spin dynamics of radical pair reactions using Monte Carlo trace sampling, which significantly outperformed existing methods, in particular for systems with large numbers of nuclear spins. Five years later, Fay et al.^17^ extended this approach, using the stochastic Schrödinger equation (SSE) to include spin-relaxation processes induced by time-dependent interactions. Here, we develop a generalized framework for SSE simulations of arbitrary radical pair systems using *MolSpin*, a software package designed to efficiently model spin-dependent reactions.^15,18^ It provides a comprehensive suite of functionalities to study the impact of spins on chemical transformations. The intended key attributes include a generalized approach, user-friendly extendibility, and an intuitive input interface, all of which should promote flexibility in modeling spin-dependent chemical reactions. Moreover, for ease of use, *MolSpin* is designed for seamless integration with the web-based platform VIKING (Scandinavian Online Kit For Nanoscale Modeling).^19^

The methods presented here for handling generalized trace-sampled SSEs provide new features, such as correction terms and different propagators. Furthermore, direct linking to molecular dynamics (MD) and quantum chemical (QC) simulations allows complex multiscale problems to be studied. The range and versatility of the SSE framework are demonstrated using selected examples of the RPM.

## Theory and Implementation

The spin dynamics of radical pairs can be described by the Liouville–von Neumann equation, which may be written (expressing energies as angular frequencies) as
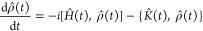
1where ρ̂(*t*) is the density operator of the combined electron–nuclear spin system and [·, ·] and {·,·} denote the commutator and anticommutator, respectively (ℏ = 1). The spin Hamiltonian that characterizes the physical interactions of the system is defined as
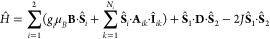
2where the terms in the brackets represent the Zeeman interaction of the *i*th electron spin, **Ŝ**_*i*_, with the external magnetic field, **B**, and the hyperfine interaction of the *i*th electron spin with the *k*th nuclear spin, **Î**_*ik*_, via the coupling tensor **A**_*ik*_ (*N*_*i*_ is the total number of nuclear spins coupled to the *i*th electron). *g*_*i*_ is the *g*-value of radical *i*, and μ_B_ is the Bohr magneton. The tensor **D** describes the dipolar coupling between the two electron spins, while the scalar *J* is the electron exchange interaction.

The spin-dependent reaction operator *K̂*(*t*) in eq 1 is given by^20^
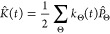
3where *k*_Θ_(*t*) is the rate coefficient for the reaction of state Θ∈{S, T_+1_, T_0_, T_–1_} and the operator *P̂*_Θ_ projects onto Θ.

Exact numerical solution of eq 1 is feasible for small spin systems but becomes computationally challenging for larger numbers of nuclei because of the exponential growth of the Hilbert space. Further complications arise from the interactions of the spins with the fluctuating environment, which leads to spin relaxation. Fortunately, the scaling can be improved using a SSE method, which can incorporate explicit time-dependent interactions and Monte Carlo sampling of the initial nuclear spin configuration to enable larger spin systems to be handled.^17,21^

Following ref (17), we now summarize an efficient formalism for multispin systems, including spin relaxation. The first step is to separate the time-independent and time-dependent parts of the spin Hamiltonian by writing *Ĥ*(*t*) in terms of stochastically fluctuating functions *f*_*j*_(*t*) and spin operators *Â*_*j*_(22,23):
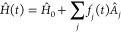
4

Any time dependence of the reaction operator *K̂*(*t*) may be included via the rate coefficients *k*_Θ_(*t*), which are normally not functions of time but can be made so in specific cases, for example, when the inter-radical separation is sinusoidally modulated.^24^ The quantum yield, Φ_Θ_, of the reaction of a specific electronic spin state is given by
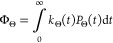
5where:
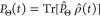
6ρ̂(*t*), the solution of eq 1, is calculated using the propagator *Û*(*t*,0):
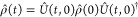
7*Û*(*t*_1_, *t*_0_) propagates the system from *t*_0_ to *t*_1_ and is defined in terms of the time-ordering operator, *T̂*:
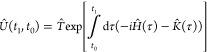
8

The initial state of the system is represented as the Kronecker product of the initial electronic spin state |Θ_init_⟩ (i.e., singlet or triplet) and the identity operator, *Î*_*z*_, for the nuclear spins:
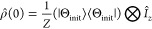
9where *Z* is the dimension of the nuclear spin Hilbert subspace.

In the following, we outline two approaches for evaluating the trace in eq 6, termed direct and stochastic methods. The direct method propagates all possible nuclear spin states and is suitable for small spin systems, while the stochastic approach uses Monte Carlo sampling for the efficient simulation of spin systems with large Hilbert spaces. The two methods enable a wide range of radical pair reactions to be studied.

### Direct Method

We can evaluate the quantum mechanical trace in the following basis^16^:
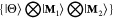
10where |Θ⟩ is the electron-spin state and |**M**_*i*_⟩, the nuclear spin state of radical *i*, is given by
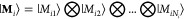
11

*M*_*ik*_ is the magnetic quantum number of the *k*th nuclear spin in radical *i*. We can expand the trace in eq 6 using eq 10 and summing over all nuclear spin states, |**M**_*i*_⟩:
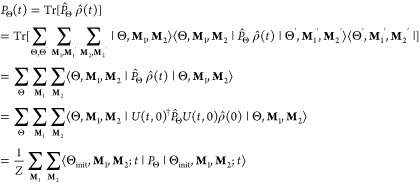
12

The transition from the third line to the fourth in eq 12 is accomplished using eq 7 and we have used the property that operators can be cyclically permuted within the trace. Given eq 9, the sum over Θ vanishes, with only the Θ_init_ state surviving. The states |Θ_init_, **M**_1_, **M**_2_; *t*⟩ = *Û*(*t*,0)|Θ_init_, **M**_1_, **M**_2_⟩ follow the dynamics of the SSE^17^:
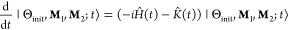
13

This direct method is efficient for small spin systems but becomes impractical when dealing with thousands of nuclear spin states.

### Stochastic Method and Trace Sampling

The requirement to propagate all *Z* states can be avoided by stochastically evaluating the trace in eq 12. This is achieved by defining a resolution of the identity for the normalized nuclear spin states |ψ(ξ)⟩, parametrized by ξ^17^:
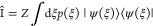
14where *p*(ξ) is the normalized probability density. Replacing *Î*_*z*_ in eq 9 by Î, the trace can be calculated as^17,21^
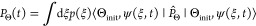
15where |Θ_init_, ψ(ξ,0)⟩ = |Θ_init_⟩ ⊗ |ψ(ξ)⟩ and the time-propagation of |Θ_init_, ψ(ξ, *t*)⟩ follows the SSE as in eq 13.

A good choice of |ψ(ξ)⟩, used throughout this article, is the SU(*Z*) coherent states denoted |**Z**⟩, where **Z** is a vector of complex numbers *Z*_*n*_ = *X*_*n*_ + *iY*_*n*_, where *X*_*n*_ and *Y*_*n*_ are randomly sampled, independent normal deviates. In a chosen basis, |**Z**⟩ is given by
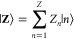
16with the constraint ⟨**Z** | **Z**⟩ = 1. SU(*Z*) states are sampled from the distribution *p*(**Z**) = δ(| **Z** | – 1)/*S*_2*Z*_, where *S*_2*Z*_ is the surface area of a 2*Z*-dimensional hypersphere of unit radius and δ is the Dirac delta function. In comparison to other sampling methods, such as the spin coherent states, the SU(*Z*) states have self-averaging properties as a result of their invariance to unitary transformations. Fay et al. performed a comprehensive analysis of different sampling methods and were able to demonstrate that the SU(*Z*) states require fewer sample states than, e.g., spin coherent states.^17^

Spin coherent states^21^ are also implemented in *MolSpin* (see manual^25^); however, we recommend SU(*Z*) for all calculations and will not discuss spin coherent states further (see refs (16,17,26) for a discussion).

The main advantage of the stochastic methods is that we can use *M* < Z Monte Carlo sampled nuclear spin configurations to be propagated, compared to *Z* state-vectors in the direct method. The condition that *M* ≪ Z for the method to be computationally efficient, means that it is not viable for small spin systems. As in the analytic derivation of Weisse et al. for stochastic trace sampling,^21^ we demonstrate that for small Hilbert-space sizes, relatively more sample states are required to deliver accurate results (Supporting Information, Section 2). For small spin systems, *M* must be comparable to *Z* to produce reliable results leading to a smaller gain in computational efficiency.

### Stochastic Schrödinger Equation

Both direct and stochastic methods rely on time-propagation of state vectors using the SSE. For a generic state |ψ(*t*)⟩, the SSE is given by
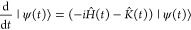
17which has the solution
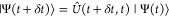
18

The propagator *Û* can be approximated, without the time-ordering operator, by using the first-order Magnus expansion for small time steps, δ*t*^27^:
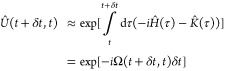
19where the generator Ω, used to propagate the spin state, is given by^17^
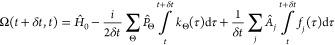
20

In general, the time-dependence of the interactions is included by providing trajectories of the fluctuating components, *f*_*j*_(*t*), for each operator in eq 20 (see Supporting Information, Section 8). For the recombination operators, the time dependence is carried by the rate coefficients, *k*_Θ_(τ). This framework allows explicit and stochastic time-dependent interactions to be included such that complex systems can be modeled, e.g., in biological environments, with minimal impact on the computation time.

Time propagation of the state vectors is straightforward, using eqs 18–20 to find expectation values and quantum yields. In simple terms, this means evaluating *e*^A*t*^·**B**, where **A** is an *n* × *n* matrix and **B** is an *n*-element vector. *MolSpin* performs this propagation using either the short iterative Lanczos method^28^ or the short iterative Arnoldi method,^29^ both of which are Krylov subspace techniques. *MolSpin* also uses “*autoexpm*”,^30^ an alternative algorithm that seems to be new to the field of spin dynamics (see *MolSpin* manual^25^ and Supporting Information, Section 3), which has been employed for all the examples presented here.

Due to the sparse nature of the spin Hamiltonian, the state-vector propagation scales as Ο[*Z* log *Z*].^16^ The direct method, in which *Z* state-vectors are propagated for *N*_*t*_ time-steps, therefore scales as Ο[*N*_*t*_*Z*^2^ log *Z*]. The stochastic method, on the other hand, requires propagation of only *M* Monte Carlo samples, reducing the overall scaling to Ο[*N*_*t*_*M Z* log *Z*]. For SU(*Z*) states in the stochastic method, the error scales as .^17^ This is a significant improvement compared to spin coherent states, which have an error scaling of .^16^ For very large systems, only one state may be sufficient to obtain results to graphical accuracy, allowing systems with 20 or more coupled nuclear spins to be treated (a more detailed analysis is given in Supporting Information, Section 2).

### Correction Factor

One of the challenges faced by both direct and stochastic methods is the explicit numerical evaluation of the integral in eq 5. The computational cost increases with the lifetime of the radical pairs, and it may be difficult to calculate the *f*_*j*_(*t*) terms in eq 4 (e.g., using MD simulation methods) for the complete lifetime of the radical pairs. An extrapolation method that reduces the required number of time steps is therefore desirable.

In the Supporting Information (section 1), we derive a method for evaluating the reaction yields that can significantly reduce the total propagation time. For a time-independent rate constant, the product yield is given by
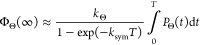
21where *k*_sym_ (= *k*_S_ = *k*_T_) is the rate constant for the spin-selective recombination. By careful choice of the integration time, *T*, this approximation can reduce the time required to calculate Φ_Θ_(∞) by up to a factor of 6 without sacrificing numerical accuracy (Supporting Information, Section 1). With care, the approach is also applicable to time-dependent Hamiltonians.

### Features of the SSE Approach in *MolSpin*

The *MolSpin* software package is designed to provide a framework to address a variety of spin-dependent reactions. It reduces the need to construct new spin Hamiltonians and related components for each specific case. In the context of the SSE method, *MolSpin* accommodates all linear and bilinear (single-spin and two-spin) interactions, offering the user flexibility in customizing these interactions.^15^ To enhance its adaptability, *MolSpin* allows direct parsing of complex time-dependent interactions and rate coefficients. Comprehensive documentation on this file format is available in the Supporting Information (Section 8 and in the *MolSpin* manual^25^).

The implementation of SSE methods in *MolSpin* supports various initial spin states for the radical pair, including S, T_0_, T_–1_, and T_+1_. Mixed initial states can be simulated by combining results from each of the states involved. As mentioned above, there are two distinct methods, *direct* for small spin systems and *stochastic* for large spin systems, which use either *Krylov* subspace or *autoexpm* algorithms (discussed in Supporting Information, Section 3) for state-vector propagation. For the *stochastic* method, *MolSpin* includes SU(*Z*) states and spin coherent states.

*MolSpin* provides several different approaches for calculations using direct and stochastic methods (Supporting Information, Table S3). Additional information is available in the *MolSpin* manual.^25,31^

## Results

In this section, a demonstration of the capabilities of the generalized framework for simulating RPM spin dynamics is provided by replicating established results and by exploring new radical pair systems. First, systems of more than 15 spins are simulated with a static spin Hamiltonian, including phenomena such as charge separation along a “molecular wire”, to mimic photosynthetic electron transfer,^32^ and anisotropic reaction yields in the context of magnetoreception.^33^ Next, recent work on driven dynamics of radical pairs, in which evidence was found for the enhancement of the anisotropy of reaction yields, is revisited.^24^ This is followed by calculations of spin-relaxation effects in the [FAD^•*–*^ TrpH^•+^] radical pair in European robin cryptochrome-4a induced by fluctuating hyperfine interactions extracted from MD simulations. Finally, we explore regimes, motions, and spin-system sizes that have not been studied previously due to the limitations of earlier methods.

### Numerical Accuracy of the Stochastic Method

**Figure 1:**
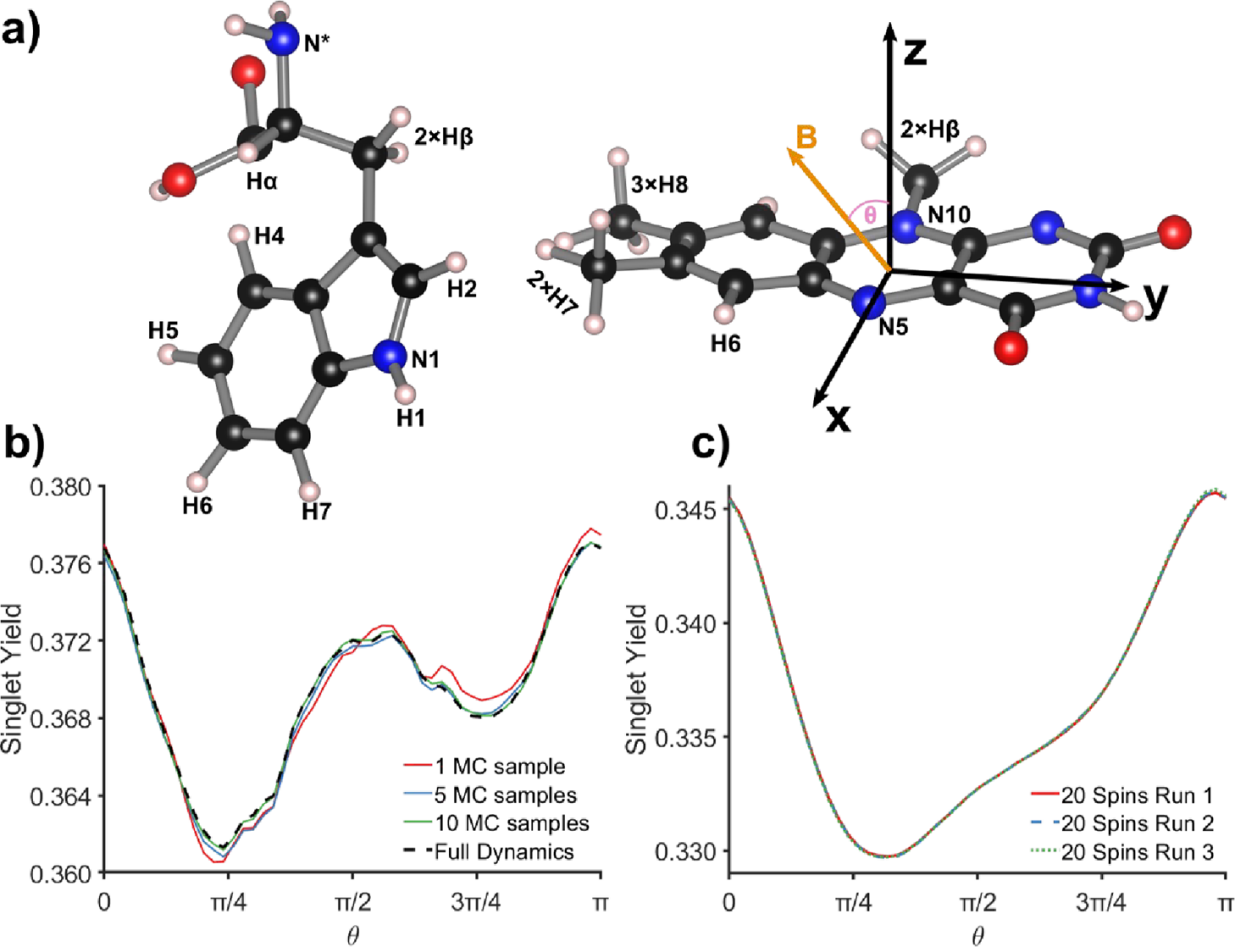
(a) Schematic of the [FAD^•–^ TrpH^•+^] radical pair model (TrpH on the left, FAD on the right), with the atoms used in the calculations labeled. (b) Singlet yield, Φ_S_, as a function of the magnetic field direction, θ, for a 12-nucleus [FAD^•–^ TrpH^•+^] radical pair model. The full spin dynamics, using matrix diagonalization,^34,35^ is compared to calculations made using the SSE method, with *M* = 1, 5, or 10 SU(*Z*) spin states. The integration time, *T*, for calculating the quantum yields was 6908 ns (eq 21) and the time-step was 4 ns. (c) Singlet yield, Φ_S_, as a function of the magnetic field direction, θ, for a 20-nucleus [FAD^•–^ TrpH^•+^] radical pair model (i.e., a 22-spin system). Because the full calculation was impossible for a spin system of this size, the stochastic simulation was performed three times using the different single (*M* = 1) SU(*Z*) spin states (runs 1–3), with almost identical results. The integration time, *T*, was 693 ns (4 ns time step). In panels (b) and (c), the magnetic field strength was 1 mT and the symmetric recombination rate constant *k*_sym_ (= *k*_S_ = *k*_T_) was 1 μs^–1^. To demonstrate the capability of the *MolSpin* method, nuclear spins were chosen to maximize the dimension of the Hilbert space rather than on the basis of their hyperfine tensors. The *MolSpin* input file can be found in the Supporting Information (Section 8).

To assess the accuracy of the stochastic method as implemented in *MolSpin*, we compared it with an exact spin dynamics method that requires the spin Hamiltonians of the two radicals to be diagonalized.^34,35^ Briefly, the full Hilbert space is divided into separate subspaces, one for each radical, so that the spin evolution of the radicals can be calculated separately. The method from refs (34) and (35) allows relatively large spin systems to be handled, provided there are no exchange or dipolar interactions of the radical pair. The spin system chosen for this test was a model of [FAD^•*–*^ TrpH^•+^], the magnetically sensitive radical pair in cryptochrome (FAD = flavin adenine dinucleotide, TrpH = tryptophan). The system comprises two electron spins and 12 nuclear spins: N5, N10, H6, 3 × H8, Hβ in FAD^•*–*^ and N1, H1, H2, H4, H6 in TrpH^•+^ (see Figure 1a), with spin quantum numbers  for ^1^H and *I* = 1 for ^14^N. The anisotropic hyperfine tensors were taken from ref (33), and the dipolar and exchange parameters from ref (36). Φ_S_ was calculated as a function of the angle, θ, between the flavin *z*-axis and the magnetic field vector, **B**, in the *z-x* plane (defined in Figure 1a). A long propagation time of 6908 ns was chosen to avoid any numerical error when comparing the trace-sampling results to the exact method.

A single SU(*Z*) nuclear spin state (*M* = 1) was sufficient to capture the essential details of the orientation dependence (see Figure 1b). With *M* ≥ 5, the stochastic approach agrees with the exact calculation to within plotting accuracy. That this can be achieved with only 5 samples can be attributed to the self-averaging property of SU(*Z*) states and the effectiveness of Monte Carlo sampling for large nuclear spin spaces. The complete Hilbert space of the spin system in Figure 1b has dimension 55,296, which is close to the largest feasible size using the direct method.^34,35^ While *Ĥ* has a sparse matrix representation, the same is not true of its eigenvectors. Like other matrix diagonalization techniques, the QR algorithm employed here requires the matrices to be stored in a dense format. For the 14-spin system, 45.6 GB of RAM is required. In addition, the time required to diagonalize a matrix of dimension *n* scales as Ο[*n*^3^]. The SSE implementation does not require diagonalization of *Ĥ* and uses sparse matrices within the Armadillo framework^37^ to overcome these limitations.

To demonstrate the power of the stochastic method, we performed a large-scale simulation of [FAD^•*–*^ TrpH^•+^] (to the best of our knowledge, the largest hitherto attempted without explicit approximations). The results are shown in Figure 1c. The system consisted of 20 nuclear spins: N5, N10, H6, 3 × H8, 2 × Hβ, 2 × H7 in FAD^•*–*^, and N1, N*, H1, H2, H4, H6, H7, 2 × Hβ, Hα in TrpH^•+^. Here, an ε-value of 0.5 was chosen for the propagation to decrease the computational time. The Hilbert-space dimension (21,233,664) is such that dense-matrix methods would require 6561 TB of RAM to calculate a quantum yield. The spin Hamiltonian stored as a sparse matrix is 88.6 GB (approximate upper bound: the memory used by *MolSpin* was only 32.88 GB), a size that modern computer clusters can handle. Figure 1c also reveals the importance of including as many nuclear spins as possible when simulating the dependence of the product yield on the direction of the magnetic field. The loss of some of the features in Figure 1b seems to result from the added nuclei having hyperfine tensors with different symmetries, such that some of the anisotropic effects are “smeared out”. A similar trend has been found for smaller spin systems.^12^ In subsequent sections, we highlight the features and flexibility of the SSE methods in *MolSpin* and extend our calculations to include protein motions in cryptochrome as a way of exploring their effects on the spin dynamics of radical-pair-based magnetoreception.

### Molecular Wires as Models of Photosynthetic Reaction Centers

**Figure 2:**
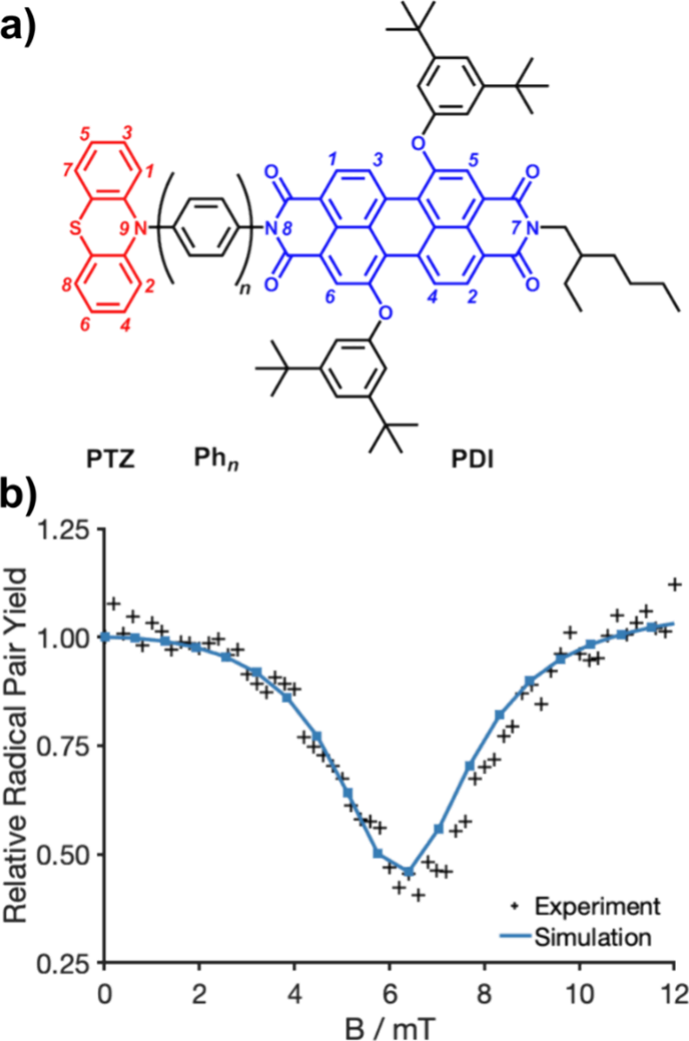
(a) Chemical structure of the PTZ–Ph_*n*_–PDI molecular wire with the donor (PTZ) shown in red and the acceptor (PDI) shown in blue. The 17 labeled nuclei were included in the spin dynamics calculation. (b) Dependence of the relative radical pair yield on the magnetic field strength for a PTZ^•+^–Ph_4_–PDI^•–^ radical pair. Experimental data (crosses) were taken from Weiss et al.^32^ The *MolSpin* input file can be found in the Supporting Information (Section 8).

Molecular wires^32,38^ are typically donor-bridge-acceptor molecules designed to mimic the efficient long-range charge transport found in photosynthetic reaction center proteins.^39^ Weiss et al.^32^ have measured triplet and radical yields in PTZ^•+^–Ph_*n*_–PDI^•*–*^ systems, consisting of a phenothiazine (PTZ) electron donor, a perylene-3,4:9,10-bis(dicarboximide) (PDI) acceptor, and a bridge of *n* = 2–5 para-phenylene rings. The structure is shown in Figure 2a. Transient absorption data for these compounds have been modeled successfully by Fay et al.^40^ In this section, we replicate the simulation for the molecular wire with 4 para-phenylenes separating the donor and acceptor. One of the main challenges in computing the quantum yield is the number of nuclei to be considered: 17 in total (Figure 2a).

The spin Hamiltonian used to model these radical pairs in solution is given by
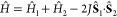
22
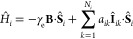
23where γ_e_ is the gyromagnetic ratio of the electron and *a*_*ik*_ is the isotropic hyperfine coupling constant of nucleus *k* in radical *i*. The spin dynamics follow eq 1. The 17 nuclei (^1^H, ^14^N) labeled in Figure 2a were included in the calculations. The hyperfine couplings were taken from experimental measurements in ref (32), and the signs of the interactions were determined by DFT calculations.^40^ The exchange interaction was 2*J* = 6.4 mT and the experimentally determined recombination rate constants for the triplet and singlet states were *k*_T_ = 350 μs^–1^ and *k*_S_ = 2.45 μs^–1^, respectively.^40^

The experiments for the *n* = 4 molecular wire measured the relative radical pair yield, Φ_RP_(*B*, *t*)/Φ_RP_(0, *t*), *t* = 50 ns after photoexcitation,^32^ where
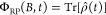
24

Φ_RP_(*B*, *t*)/Φ_RP_(0, *t*) was calculated using Monte Carlo sampling with the SU(*Z*) spin-states method for a time-independent Hamiltonian (*StaticHS-StochYields*, Supporting Information, Table S3). Only one SU(*Z*) state was required to simulate the system, compared to the 200 spin coherent states required in ref (40), reflecting the error scaling properties mentioned above. The relative yield of the radical pair calculated in this way is compared with the experimental data in Figure 2b. The influence of the magnetic field is reproduced, revealing a minimum in the yield at around 6.4 mT as expected for a “2*J*-resonance”.^40^

### Singlet Yield Anisotropy in a Model Magnetoreceptor

The RPM hypothesis of magnetoreception in migratory birds has attracted attention in recent years.^4−7,41^ It proposes that photoinduced radicals in cryptochrome flavoproteins could enable a bird to detect the direction of the geomagnetic field as the basis of a navigational compass. Specifically, it has been suggested that the sensor comprises FAD^•–^ and TrpH^•+^ radicals formed by a cascade of light-induced electron transfers within the protein.^5^ In a computational study, Hiscock et al.^33^ examined the [FAD^•*–*^ TrpH^•+^] spin system and found a pronounced “spike” in the reaction yield when the magnetic field vector was in the plane of the tricyclic flavin group (θ = 90°) and the spin-coherence lifetime was more than ∼10 μs. This feature was interpreted as arising from avoided energy-level crossings, a purely quantum mechanical effect.^36^ Due to computational limitations, the calculations were restricted to 14 nuclear spins, with no exchange or dipolar coupling.^33^

**Figure 3:**
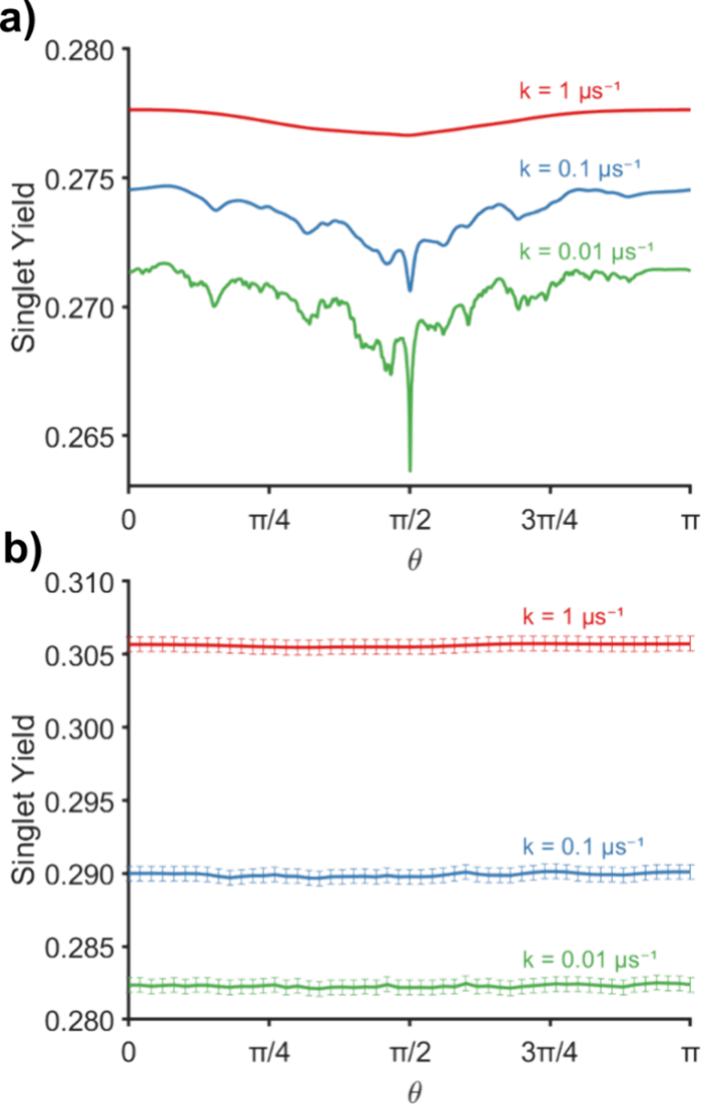
(a) Graphs of singlet yield, Φ_S_, as a function of the magnetic field direction, θ, for radical pairs with symmetric recombination rate constants between 1 and 0.01 μs^–1^, and no inter-radical couplings. The calculations were performed using the *MolSpin* task described earlier.^18^ For clarity, the *k* = 0.1 μs^–1^ graph has been shifted vertically by +0.0025. (b) As a) except that electron–electron couplings were included and the calculation was performed using the SSE method with one SU(*Z*) state. The evaluation of the errors arising from the Monte Carlo sampling is described in the Supporting Information (Section 2). The time-step for propagation was set to 4 ns. Total integration times for evaluating singlet yields were 0.691 μs for *k* = 1 μs^–1^, 6.91 μs for *k* = 0.1 μs^–1^, and 69.08 μs for *k* = 0.01 μs^–1^. In both panels (a) and (b), 14 nuclear spins were included: N5, N10, H6, 3 × H8, and Hβ in FAD, and N1, H1, H2, H4, H6, H7, and Hβ1 in TrpH. In both cases, the radical pairs were initially in a singlet state. The magnetic field strength was set to 50 μT in both cases. The *MolSpin* input file can be found in the Supporting Information (Section 8).

In Figure 3a, we reproduce the calculation from an earlier study^33^ for the 14-nucleus [FAD^•*–*^ TrpH^•+^] radical pair (structure in Figure 1a). Figure 3b shows the results of a similar calculation, now performed using the SSE approach with dipolar and exchange interactions included. The full coupling tensor, **C** = **D** – 2*J***I** was^36^
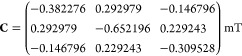
25

Figure 3b shows that when electron–electron coupling is included, the distinct spike (visible at θ = 90° in Figure 3a) is strongly attenuated, as found previously.^42^ This observation does not necessarily preclude the occurrence of a spike in systems with specific inter-radical couplings. One possibility is that it could be restored by partial cancellation of the effects of exchange and dipolar interactions.^43^ Nevertheless, Figure 3 illustrates that large systems and long radical pair lifetimes (1–100 μs) can be effectively simulated by using the stochastic method implemented in *MolSpin*.

### Driven Dynamics in Magnetoreception

Motivated by the expected harmful impact of inter-radical spin interactions on the anisotropy of the reaction yield (illustrated in Figure 3), various authors have explored additional factors that might compensate for these effects. Smith et al. introduced the concept of a “live” radical pair with a coherently driven distance between the radicals and hence coherently modulated recombination rate coefficients, exchange, and dipolar interactions.^24^ Their analysis concluded that such a dynamic system could, in principle, exhibit greater sensitivity to the direction of a weak field than that of its static counterpart. The authors went on to suggest potential scenarios that might lead to harmonic oscillations, including structural rearrangements following the initial charge transfer or sensory transduction,^44^ and changes in protein structure due to proton pumping.^45^ This proposal remains hypothetical and has not been experimentally validated. Nevertheless, it provides an opportunity to demonstrate the implementation of time-dependent spin interactions by using the SSE method in *MolSpin*.

**Figure 4:**
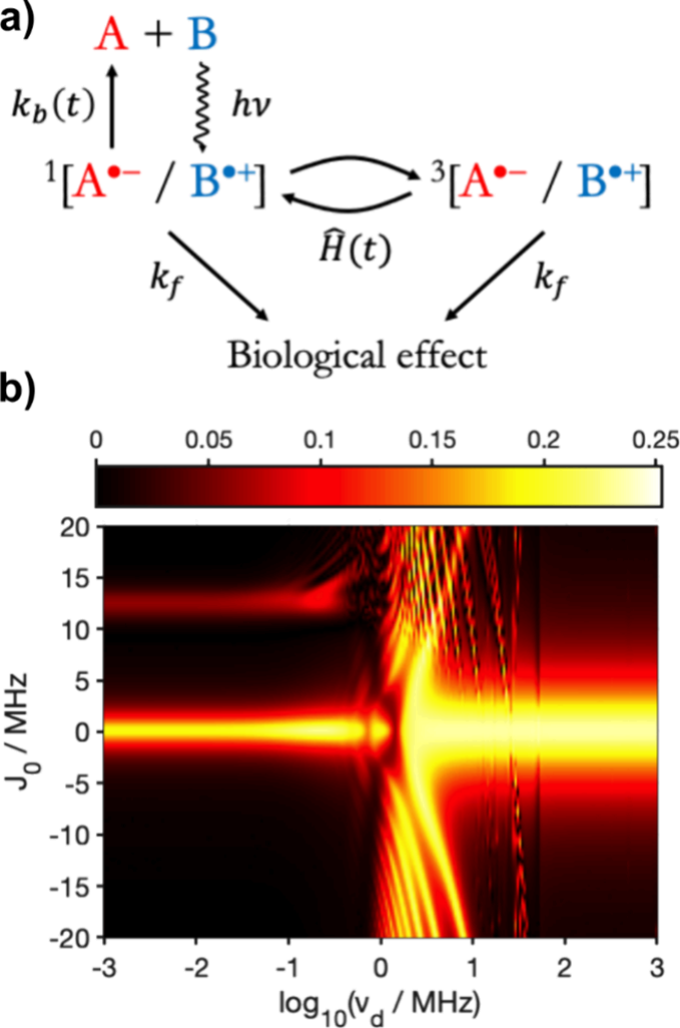
(a) Reaction scheme for a driven radical pair model in which the inter-radical separation is coherently modulated. (b) Heat map of the relative anisotropy, χ (eq 30) as a function of the exchange interaction, *J*_0_, and the driving frequency, *v*_d_. The *MolSpin* input file can be found in the Supporting Information (Section 8).

The master equation used here corresponds to the reaction scheme in Figure 4a and includes time-dependent singlet–triplet interconversion, forward reaction of singlet and triplet states (rate constant *k*_f_), and time-dependent backward recombination of the singlet state to the ground state (rate coefficient *k*_b_(*t*)).^24^ The effective Hamiltonian^24^:
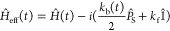
26was employed to account for both the spin interactions and the reactivity.

To demonstrate the feasibility of such a calculation in *MolSpin*, we repeated an earlier simulation^24^ consisting of a single hyperfine-coupled nitrogen atom (spin *I* = 1) in one radical, with no hyperfine interactions in the other radical. The inter-radical distance was taken to be^24^
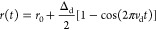
27where Δ_d_ = 3 Å is the amplitude of the oscillation, *v*_d_, the frequency of the oscillation, ranged from 1 kHz to 1 GHz, and *r*_0_ = 17.8 Å.^24^

The singlet recombination rate coefficient and the electron exchange interaction were^24^
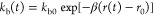
28
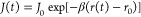
29with *k*_b0_ = 2 μs^–1^, *k*_f_ = 0.5 μs^–1^, and β = 1.4 Å^*–*1^.^46^ This value of takes into account an extraneous factor of 1/2 in eq 3 in ref (24). The exchange parameter, *J*_0_, was varied between −20 and +20 MHz. A dipolar interaction was not included. The single hyperfine interaction was assumed to have the same axial symmetry and magnitude as the N5 atom in FAD^•−^, with principal components *A*_*xx*_ = *A*_*yy*_ = −2.6 MH*z* and *A*_*zz*_ = 49.2 MH*z*. The magnetic field was 50 μT.

To evaluate the directional magnetic field effect, the relative anisotropy χ was calculated
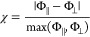
30where Φ_∥_ and Φ_⊥_ are the singlet yields of the radical pair recombination reaction computed for a static magnetic field parallel or perpendicular to the *z*-axis of the hyperfine interaction. The resulting variation of χ for a range of *J*_0_ and *v*_d_ values is shown in Figure 4b. In the static case (*v*_d_ = 0), inter-radical coupling suppresses the magnetic field sensitivity when |*J*_0_| ≥ 1 MHz. However, as the driving frequency increases, this effect is mitigated, such that when *v*_d_ is in the range of 1–10 MHz an appreciable magnetic field effect is predicted across the range of *J*_0_ values considered. Figure 4b is in excellent agreement with the previous calculation.^24^

Artificial model Hamiltonians may, however, not always be suitable for describing motions that might induce spin relaxation. Therefore, it is also crucial to have the ability to use dynamical data extracted from multiscale approaches, such as those combining quantum chemical (QC) calculations and MD simulations. The next section illustrates how *MolSpin* can address such challenges.

### Simulation of Realistic Spin Systems Using MD Trajectories

Reliable simulation of the spin dynamics of radical pairs in proteins requires an accurate description of the thermal motions that modulate electron-spin interactions and cause the spins to relax. The nature of these structural fluctuations can be determined from MD simulations and their effects on, for example, hyperfine tensors can be calculated using QC methods.^12,13^ The challenge is how to incorporate this information into a spin dynamics calculation. One approach is to use perturbative spin-relaxation theories, such as Bloch–Redfield–Wangsness, which are computationally intensive for large spin systems and inapplicable when the molecular dynamics are not much faster than the spin evolution.^17^ A more satisfactory method is to include the time-dependence of the spin interactions explicitly using the SSE technique.

**Figure 5:**
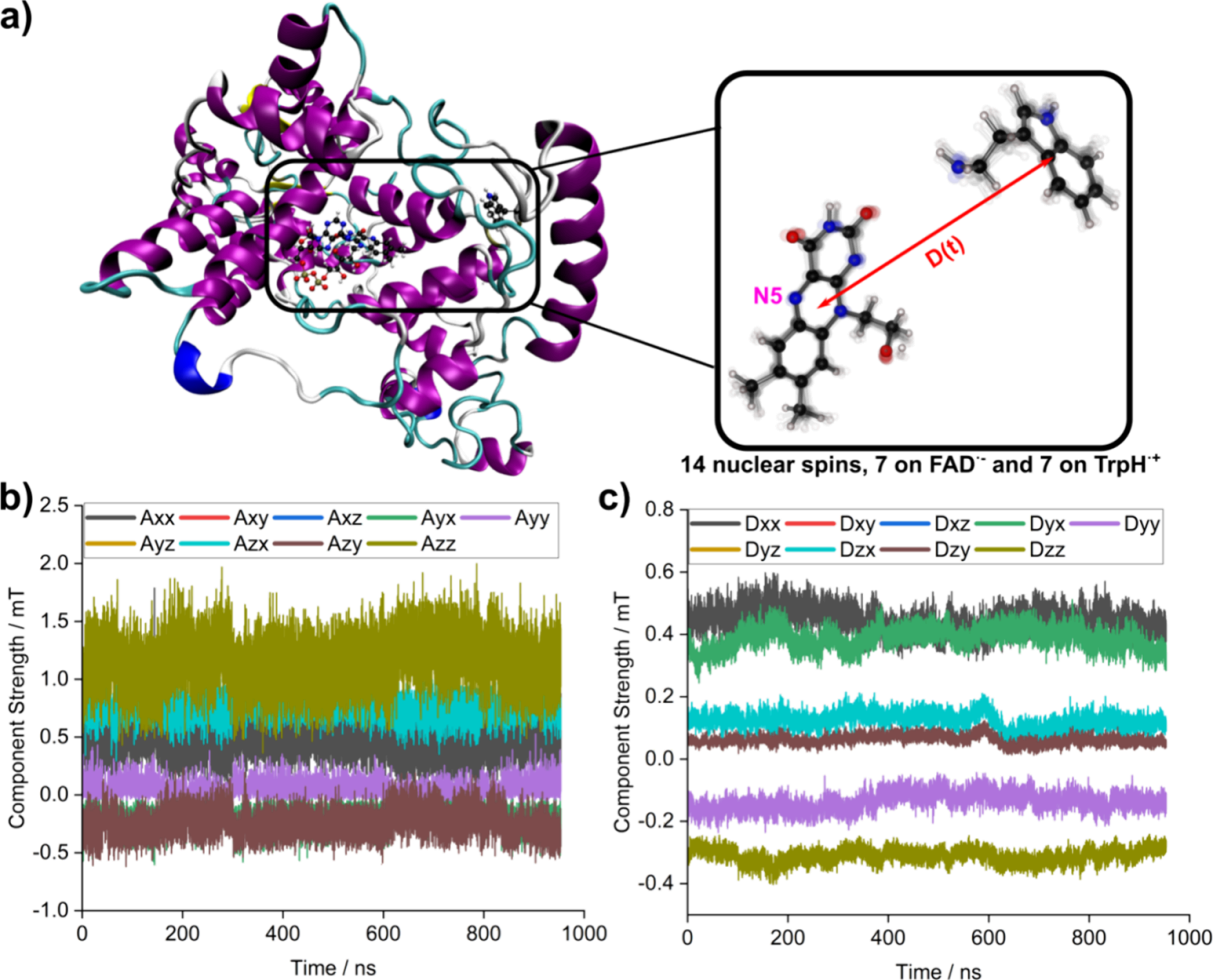
(a) Structures of European robin cryptochrome-4a and the FAD^•–^ and TrpH^•+^ radicals considered in the MD simulation. (b) Fluctuations in the components of the hyperfine tensor of the N5 nucleus in FAD^•–^. The length of the MD trajectory was 0.953 μs. (c) Fluctuations in the dipolar coupling of the FAD^•–^ and TrpH^•+^ radicals.

To illustrate this approach, the spin dynamics of [FAD^•–^ TrpH^•+^] in European robin cryptochrome-4a (see Figure 5a) were calculated using an MD simulation from an earlier study^12^ extended to 0.95 μs. Every 50 ps throughout the MD trajectory, the geometries of the FAD^•–^ and TrpH^•+^ radicals (W318, the third component of the Trp-tetrad) were extracted as described previously,^12,13^ optimized, and the hyperfine tensors of the 14 nuclei calculated using the hybrid-functional B3LYP and the EPR-II basis set (see Supporting Information, Sections 6 and 7) employing Gaussian16.^47^ As an example, Figure 5b,c shows the time-dependence of the hyperfine components of the N5 atom in FAD^•–^ and of the dipolar coupling of the two radicals. Further details, including a list of the 14 hyperfine coupling tensors, are given in the Supporting Information (Section 7).

**Figure 6:**
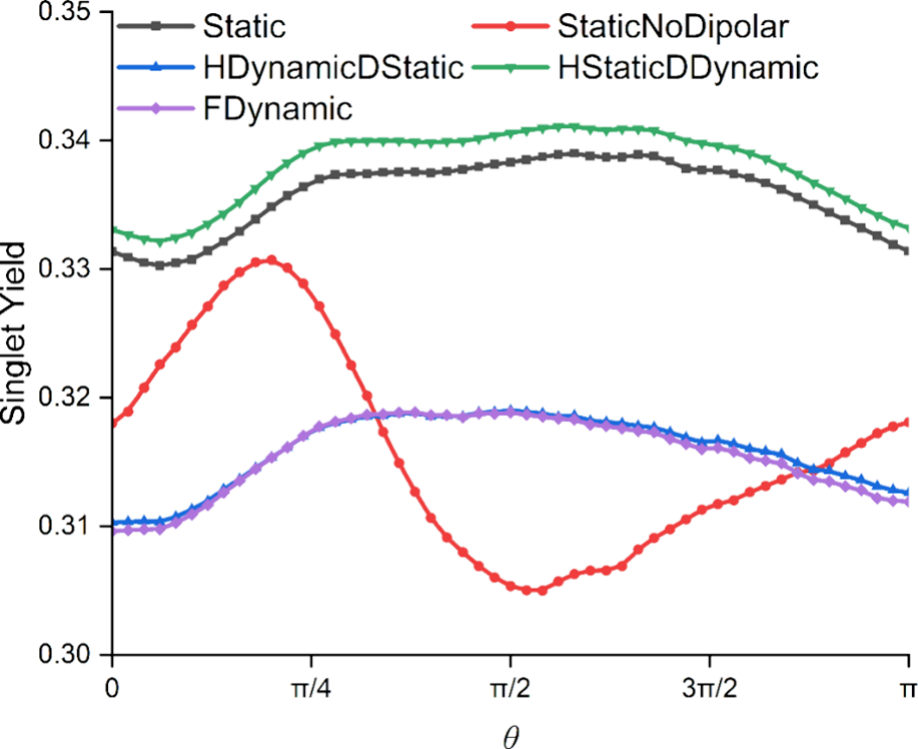
Singlet yields of a 14-nucleus radical pair with a 1 μs^–1^ symmetric reaction rate constant, subject to a magnetic field of 1 mT, as a function of the direction of the field (θ is defined in Figure 1a). The dynamic Hamiltonian was constructed by using a 50 ps time step for the hyperfine and dipolar interactions. Five random SU(*Z*) nuclear spin states were used. The total integration time for calculating the singlet yield was 953.0 ns. The correction factor method was employed to produce singlet yields. The *MolSpin* input file can be found in the Supporting Information (Section 8).

The results of the singlet yield calculations are listed in Figure 6. The lifetime of the singlet-born radical pair was taken as 1 μs, with equal singlet and triplet reaction rate constants. For this demonstration, the singlet yield was calculated for 51 directions of a 1 mT external magnetic field. The quantum yield for a static Hamiltonian (*Static*) was calculated and compared to the full dynamic Hamiltonian for the hyperfine and dipolar interactions (*FDynamics*). To give insight into the origin of the spin relaxation effects, three further simulations were performed: one using the static Hamiltonian with the dipolar interaction removed (*StaticNoDipolar*), and two in which either the dipolar interaction (*HDynamicDStatic*) or the hyperfine interactions (*HStaticDDynamic*) were not allowed to fluctuate.

The static scenario without a dipolar interaction (*StaticNoDipolar*) gives a reaction yield anisotropy (Figure 6) similar to that in an earlier study.^48^ Inclusion of a static dipolar coupling (*Static*) reduces the anisotropy and shifts the positions of the maximum and minimum singlet yields. When fluctuations in the dipolar coupling are introduced (*HStaticDDynamic*), the reaction yield differs only slightly from the *Static* case, suggesting that little spin relaxation results from modulation of this interaction. By contrast, incorporating the time-dependence of the 14 hyperfine interactions and keeping the dipolar coupling static (*HDynamicDStatic*) markedly changed the singlet yield. The quantum yield is substantially reduced in line with previous studies.^13−15^ Furthermore, there is little effect of adding the time-dependence of the dipolar interaction (*FDynamic*), again supporting the idea that the dominant spin relaxation effects come from the hyperfine interactions.

## Conclusions

The stochastic Schrödinger equation method with trace sampling offers a powerful and efficient method for simulating the yields of spin-dependent reactions.^16,17^ In this article, we have described the implementation of the SSE in the spin-dynamics software package *MolSpin*. Large electron–nuclear spin systems can be simulated, with asymmetric spin-selective recombination kinetics, anisotropic hyperfine interactions, and nonzero exchange and dipolar couplings. Spin-relaxation can be modeled using the explicit time-dependence of spin interactions determined from MD/QC simulations or by allowing rate coefficients to be explicitly time-dependent. New features such as correction factors and propagators further enhance the SSE approach. The flexibility afforded by the *MolSpin* SSE method opens new avenues for exploring the effects of complex molecular motions on the spin dynamics of arbitrary radical pairs. *MolSpin* provides a versatile and easily extendable toolkit applicable to all kinds of spin dynamics problems. Furthermore, its direct link to MD/QC data and embedding in the VIKING^19^ web suite makes it ideal for investigating multiscale problems found in spintronics or quantum biology.

## Supporting Information Available

Correction factor for quantum yields accuracy of the stochastic method; *AutoExpm* method; implemented tasks and performance in *MolSpin*; comparison of SSE with Bloch-Redfield-Wangsness theory; time-step choice for electronic structure calculations; average hyperfine interactions from MD data; *MolSpin* features and input files (PDF)
