# Supplementary material for: Spin Dynamics of Radical Pairs Using the Stochastic Schrödinger Equation in MolSpin
Source: J Chem Theory Comput. 2024 Sep 16;20(19):8412–21. doi: 10.1021/acs.jctc.4c00361 (PMC11465467; doi:10.1021/acs.jctc.4c00361)
Supplement: Supplementary file 1 — ct4c00361_si_001.pdf [file ct4c00361_si_001.pdf]

Spin dynamics of radical pairs using the stochastic Schrödinger equation in *MolSpin* - Supporting Information

Gediminas Jurgis Pažėra[1*], Thomas P. Fay[2], Ilia A. Solov'yov[3,4,5], P. J. Hore[1], and Luca Gerhards[1*]

[1]Department of Chemistry, University of Oxford, Physical and Theoretical Chemistry Laboratory, Oxford, OX1 3QZ, United Kingdom

[2]Department of Chemistry , University of California , Berkeley , California , 94720, USA

[3]Institute of Physics, Carl von Ossietzky Universität Oldenburg, Carl-von-Ossietzky Str. 9-11, 26129 Oldenburg, Germany

[4]Research Center for Neurosensory Science, Carl von Ossietzky Universität Oldenburg, 26111 Oldenburg, Germany

[5]Center for Nanoscale Dynamics (CENAD), Carl von Ossietzky Universität Oldenburg, Institut für Physik, Ammerländer Heerstr. 114-118, 26129 Oldenburg, Germany

[*]Corresponding authors: luca.gerhards@quantum-chemistry.org, gediminaspazera@gmail.com

June 14, 2024

Contents

S1 Correction Factor For Quantum Yields **S3**

S2 Accuracy of the Stochastic Method **S12**

S3 AutoExpm Method **S14**

S3.1 *AutoExpm* Algorithm S15

S4 Implemented Tasks and Perfomance in *MolSpin* **S18**

S5 Comparison of SSE Method with Bloch-Redfield-Wangsness Theory using 550 ns MD trajectory **S19**

S5.1 Selection of Time Step for Molecular Dynamics Data (550 ns) S26

S5.2 Averaged Hyperfine Interactions for MD calculations (550 ns) S29

S6 Time-step Choice for Electronic Structure Calculations of 953 ns MD trajectory **S33**

S7 Average Hyperfines from MD data (953 ns) **S35**

S8 *MolSpin* Features & Input Files **S39**

S8.1 Format of .mst Files S39

S8.2 12 Nuclear Spin System - Figure 1b S40

S8.3 20 Nuclear Spin System - Figure 1c S41

S8.4 Molecular Wires - Figure 2b S43

S8.5 Quantum Needle - Figure 3b S45

S8.6 Driven Recombination Dynamics - Figure 4b S47

S8.7 1 Nuclear Spin Relaxation (550 ns) - Figure S6 . . . . S48

S8.8 14 Nuclear Spin Relaxation (550 ns) - Figure S7 . . . . S49

S8.9 14 Nuclear Spin Relaxation for Full Dynamic Picture (0.953 μs) - Figure 6 S51

S1 Correction Factor For Quantum Yields

We define the total yield of the products of a radical pair reaction, $\Phi_{\mathrm{Total}}(T)$, for a propagation time T when $k_{\mathrm{S}} = k_{\mathrm{T}}$, i.e. recombination constants are symmetric, as:

$$\Phi_{\mathrm{Total}}(T) = k_{\mathrm{S}} \int_0^T P_{\mathbb{I}}(t) dt \tag{S1}$$

Here $P_{\mathbb{I}}(t) = Tr\left[\hat{\rho}(t)\right]$ and $\Phi_{\mathrm{Total}}(\infty) = 1$. Since the spin-dependent radical recombination rate constants for triplet and singlet states are symmetric ($k_{\mathrm{S}} = k_{\mathrm{T}}$), then, $\hat{P}_{\mathrm{S}} + \hat{P}_{\mathrm{T}} = \mathbb{I}$ and the recombination operator simplifies to $\hat{K} = k_{\mathrm{S}}\mathbb{I}$. Hence, the radical pair yield simplifies to [1]:

$$\Phi_{\mathrm{Total}}(T) = k_{\mathrm{S}} \int_0^T e^{-k_{\mathrm{S}}t} P_{\mathbb{I}}(t) dt = 1 - e^{-k_{\mathrm{S}}T} \tag{S2}$$

In this case $P_{\mathbb{I}}(t)$ only includes dynamics due to interactions described in the spin Hamiltonian. We will denote $e^{-k_{\mathrm{S}}T}$ as ϵ for simplicity. When determining the quantum yields of various operators, it is essential to compute the integral as time approaches infinity, corresponding to the point where all radical pairs have completely decayed (i.e., when $\epsilon = 0$). Under such conditions, the following equation is valid:

$$\Phi_{\mathrm{S}}(\infty) + \Phi_{\mathrm{T}}(\infty) = \Phi_{\mathrm{Total}}(\infty) = 1 \tag{S3}$$

When evaluating the integral up to a time T, we obtain:

$$\Phi_{\mathrm{S}}(T) + \Phi_{\mathrm{T}}(T) = \Phi_{\mathrm{Total}}(T) = 1 - e^{-k_{\mathrm{S}}T} \tag{S4}$$

In practical situations, we do not integrate the expectation values to $T = \infty$. Instead, we integrate up to a point where ϵ is close to 0. Nonetheless, we also want to ensure that $\Phi_{\mathrm{S}} + \Phi_{\mathrm{T}} = 1$. Hence, we can introduce a novel heuristic:

$$\frac{\Phi_{\mathrm{S}}(T)}{1 - e^{-k_{\mathrm{S}}T}} + \frac{\Phi_{\mathrm{T}}(T)}{1 - e^{-k_{\mathrm{S}}T}} = \Phi_{\mathrm{S}}(\infty) + \Phi_{\mathrm{T}}(\infty) = 1 \tag{S5}$$

Or, in general for a projection operator Θ:

$$\Phi_\Theta(\infty) = \frac{k_\Theta}{1 - e^{-k_\mathrm{S} T}} \int_0^T P_\Theta(t) dt \tag{S6}$$

$P_\Theta(t)$ has both Hamiltonian and recombination dynamics.

The parameter ϵ serves as a total time estimator in *MolSpin* simulations. Users have the option to specify their preferred total time in ns or to define ϵ as a variable between 0 and 1, upon which the program will compute the total time as:

$$T = \frac{\ln \epsilon^{-1}}{k_\mathrm{S}} \tag{S7}$$

Some common choices of ϵ are 0.5, 0.1 and 0.01 ($\epsilon = 0.5$ will have the largest error in the quantum yield estimation and $\epsilon = 0.01$ will have the smallest error out of the three).

Our modification to the quantum yield calculations has proven effective in substantially reducing the integration time required for converged results. To illustrate this, we will examine a static 14 nuclear spin $\left[\mathrm{FAD}^{\bullet-}\ \mathrm{TrpH}^{\bullet+}\right]$ radical pair model. This model incorporates the hyperfine parameters for N5, N10, H6, $\mathrm{H8_1}$, $\mathrm{H8_2}$, $\mathrm{H8_3}$, Hβ nuclei in FAD, as well as N1, H1, H2, H4, H6, H7, $\mathrm{H}\beta_1$ nuclei in TrpH, sourced from ealier work [2]. The stochastic method was used with $M = 4$ MC samples. Notably, the system had EED and exchange interactions described in Ref. [3], and $|\mathbf{B}| = 50\ \mu\mathrm{T}$, with magnetic field vector aligned with the z-axis. Figure S1 illustrates the simulation results for the 14 spin system system under varying symmetric recombination rates, both with and without the correction factor.

Remarkably, across various recombination rate constants, the total integration time required to achieve results with graphical precision decreases distinctly. In every instance, yields calculated with the correction factor reached convergence by $\epsilon = 0.5$ ($T = T_1$), if not sooner. In contrast, without the yield correction, convergence typically requires $\epsilon = 0.01$ ($T = T_2$). Thus, the ratio of time between T_1 and T_2 is:

$$\frac{T_2}{T_1} = \frac{\ln 0.01^{-1} k_\mathrm{S}}{k_\mathrm{S} \ln 0.5^{-1}} = \frac{\ln 0.01}{\ln 0.5} \approx 6.6 \tag{S8}$$

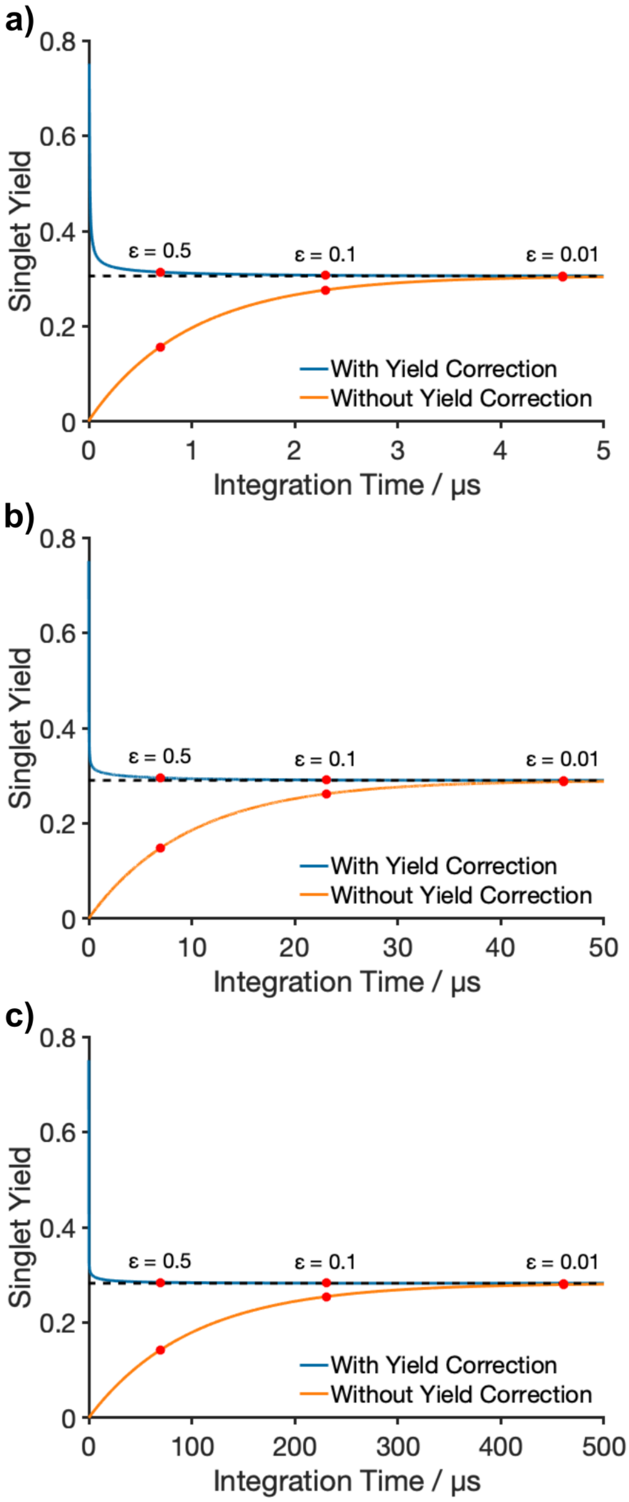

Figure S1: Singlet yield of $\left[\mathrm{FAD}^{\bullet-}\ \mathrm{TrpH}^{\bullet+}\right]$ radical pair model, Φ_S, versus integration time, T, graphs for different symmetric recombination rates: a) $k_\mathrm{S} = 1\ \mu\mathrm{s}^{-1}$, b) $k_\mathrm{S} = 0.1\ \mu\mathrm{s}^{-1}$, c) $k_\mathrm{S} = 0.01\ \mu\mathrm{s}^{-1}$. The black dashed line illustrates the converged value of the singlet yield. The system that was simulated contained 14 nuclear spins: N5, N10, H6, $\mathrm{H8_1}$, $\mathrm{H8_2}$, $\mathrm{H8_3}$, Hβ nuclei in FAD, as well as N1, H1, H2, H4, H6, H7, $\mathrm{H}\beta_1$ nuclei in TrpH sourced from ealier work [2]. Different highlighted values of ϵ correspond to times calculated by Eq. (S7) and illustrate the accuracy using the correction factor approach.

A computational reduction of at least 6 times is a significant computational speed-up that offers calculations of larger systems previously unavailable, and this reduction is achieved without compromising accuracy. The principle has not been extended for asymmetric recombination rates.

Lastly, the approach can also be extended to time-dependent dynamics. The correction factor heuristic, in principle, predicts the time-evolution of projection operators from T to ∞. In time-independent cases, this works significantly well since the interactions are static, thus, the initial trajectory within a small time interval is enough to predict the trajectory of the remaining time-dependent dynamics with a small error. However, in time-dependent calculations, where interactions are fluctuating and changing during the reaction, it is not as straightforward to apply the correction factor or to estimate the error of the singlet yield calculation. The sufficient time of propagation will depend on the type, magnitude and frequency of change of time-dependent interactions. Hence, we have examined a model system of 4 nuclear spins with varying parameters,that was inspired by the driven radical pair dynamics dynamics from Smith *et al.* [4], to arrive at a general rule of thumb of the total time choice for time-dependent systems.

The spin system contains the 4 nuclear spins: N5 and N10 in radical 1 (modeled as FAD) and N1 and H1 in radical 2 (modeled as TrpH). Hyperfine interaction tensors are taken from Ref. [2]. The symmetric recombination rate is $k_S = 1\ \mu\text{s}^{-1}$ and $|\mathbf{B}| = 1$ mT ($\mathbf{B}$ pointed in the z-direction). The time-dependent fluctuation is the exchange coupling between the electrons:

$$H(t) = -2J(t)\hat{\mathbf{S}}_1 \cdot \hat{\mathbf{S}}_2 \tag{S9}$$

Here, $J(t)$ is taken in the form of Ref. [4] as an oscillating function of time:

$$J(t) = J_0 \exp(-\beta(r(t) - r_0)), \tag{S10}$$

where $\beta = 1.4\ \mathring{A}^{-1}$ and J_0 are empirical constants [4]. The time-dependent distance $r(t)$

between the radicals is taken as:

$$r(t) = \frac{\Delta_d}{2}(1 - cos(2\pi\nu_d t)) + r_0. \tag{S11}$$

Here, $\Delta_d = 3$ Å, $r_0 = 17.8$Å, and ν_d is the driving frequency [4].

Figure S2 illustrates simulations of the described spin system with $\nu_d = 1$ MHz and different strengths of J_0 parameter. This figure aims to evaluate how the size of the time-dependent perturbation affects the accuracy of applying the heuristic to reduce the required total time of integration. J_0 is varied from 0 mT to 50 mT. In the simulation where $J_0 = 0$ mT, it is observed that a similar trend to the static case is found: the quantum yield value calculated with the correction factor quickly converges to the exact singlet yield for small integration times. This trend remains valid until $J_0 = 1$ mT, where larger oscillations appear in the singlet yield values calculated with the correction factor and longer integration times are required for convergence. Nonetheless, it has been found that $\epsilon = 0.1$ is sufficient to get converged expectation values for all cases examined in Fig. S2. If a comparison between the time integration value and the lifetime ($\tau_{Total} = 1/k_S$) of the radical pair is made the following ratio is found:

$$\frac{T_1}{\tau_{\text{Total}}} = \frac{\ln 0.1^{-1}}{k_{\text{S}}} \times k_{\text{S}} = \ln 0.1^{-1} \approx 2.3 \tag{S12}$$

Hence, we have found from this example, that an integration time of at most 2.3 × lifetime is sufficient to model time-dependent processes. The other observation found was that for smaller magnitude time-dependent variables a shorter integration time would be sufficient.

Moreover, we also analyzed the effect of the frequency of time-dependent variables on the use of the heuristic to estimate quantum yield values. We have chosen an interaction with $J_0 = 10$ mT and varied ν_d. Results are shown in Fig. S3. It is evident that higher motion frequencies lead to a more accurate approximation of the yield correction, while slower motions necessitate a longer integration time. Overall, we can conclude from the examples we analysed, that integration time equivalent to $\epsilon = 0.1$ should provide

an accurate approximation for moderately fast fluctuations with interactions of small to moderate magnitude, relative to other interactions in the system.

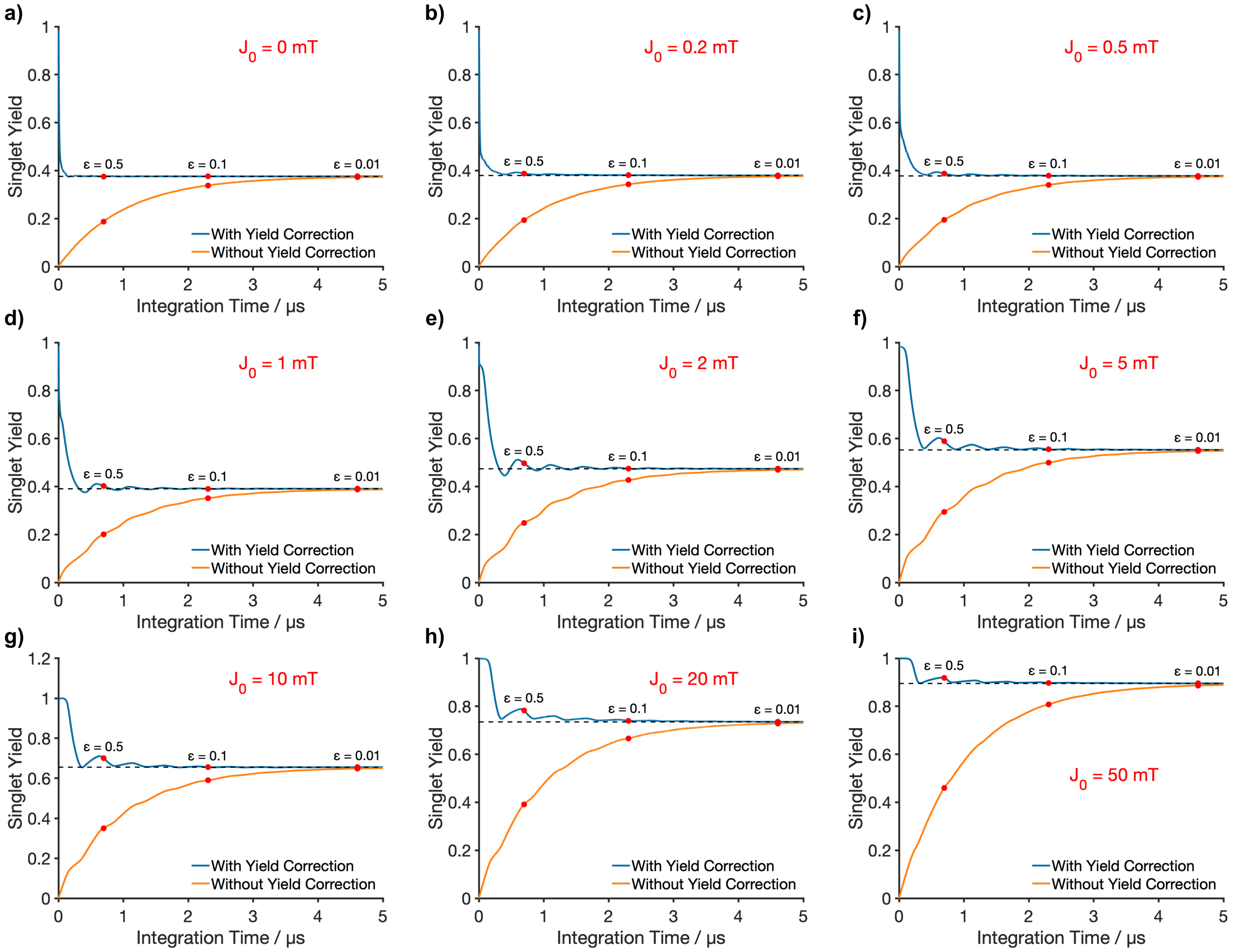

Figure S2: Singlet yield of $[\mathrm{FAD}^{\bullet-}\ \mathrm{TrpH}^{\bullet+}]$ radical pair model, Φ_S, versus integration time, T, graphs for different J_0 parameter values used to define $J(t)$ in Eq. (S10): **a)** $J_0 = 0$ mT, **b)** $J_0 = 0.2$ mT, **c)** $J_0 = 0.5$ mT, **d)** $J_0 = 1$ mT,**e)** $J_0 = 2$ mT, **f)** $J_0 = 5$ mT, **g)** $J_0 = 10$ mT, **h)** $J_0 = 20$ mT, **i)** $J_0 = 50$ mT. The black dashed line illustrates the converged value of the singlet yield. The system that was simulated contained 4 nuclear spins: N5 and N10 in radical 1 (modeled as FAD) and N1 and H1 in radical 2 (modeled as TrpH). Different highlighted values of ϵ correspond to times calculated by Eq. (S7) and illustrate the accuracy using the correction factor approach.

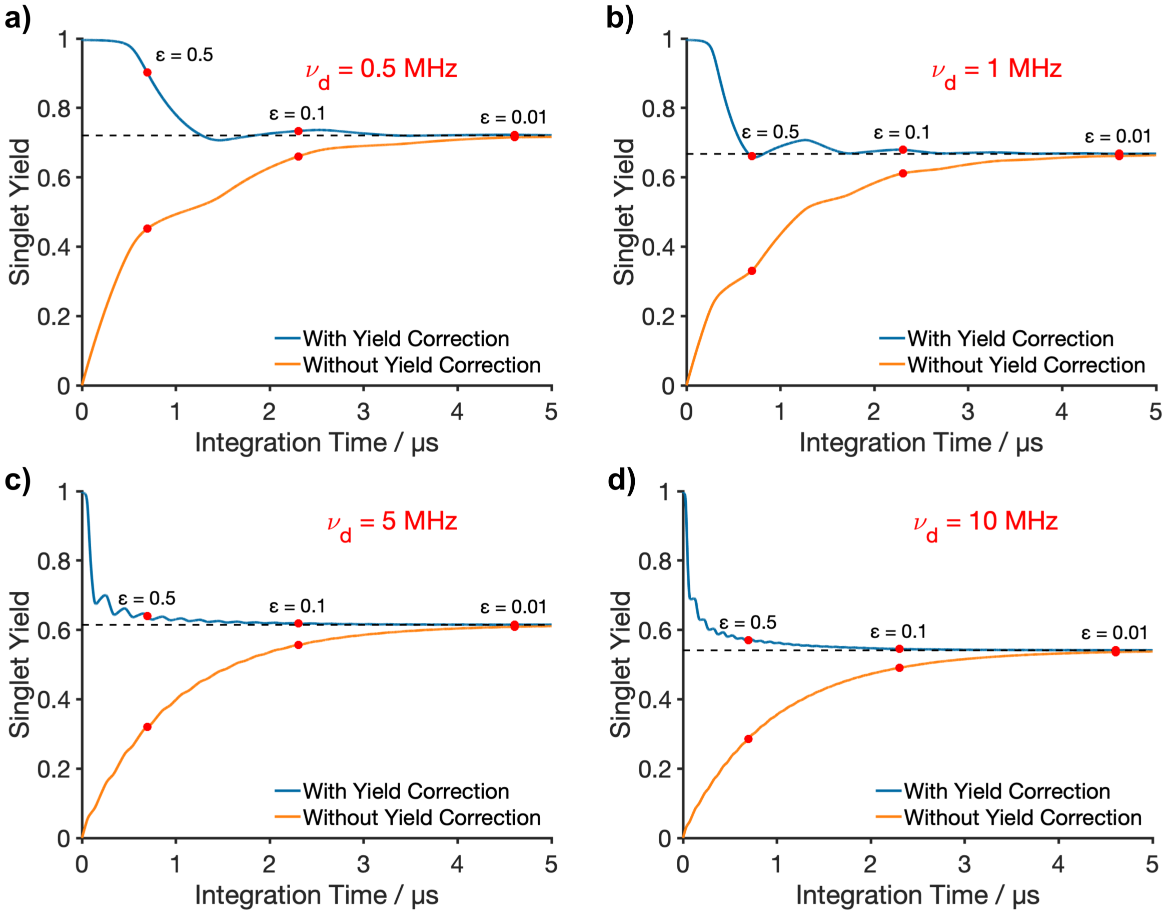

Figure S3: Singlet yield of $\left[\mathrm{FAD}^{\bullet-}\ \mathrm{TrpH}^{\bullet+}\right]$ radical pair model, Φ_S, versus integration time, T, graphs for different ν_d parameter values used to define $J(t)$ in Eq. (S10), with $J_0 = 10$ mT: **a)** $\nu_d = 0.5$ MHz, **b)** $\nu_d = 1$ MHz, **c)** $\nu_d = 5$ MHz, **d)** $\nu_d = 10$ MHz.

Finally, we have also evaluated the accuracy of the correction factor when simulating systems with interactions that follow MD trajectories as described in Section S5. MD simulations are significantly resource demanding and the trajectory of cryptochrome 4 used in Section S5 has a total time of 550 ns. For MD of proteins, a trajectory of that duration is considered unusually long. However, for spin dynamics of radical pairs which have lifetimes of the order of magnitude of 1 μs such a trajectory may be too short to yield accurate results. Hence, investigating whether the heuristic could shorten the integration time is relevant to the simulation of the MD data because we would hope to simulate systems with lifetimes of the order of magnitude of 1 μs.

In this work, the main reason for estimating longer lifetime trajectories of radicals than the MD data provides is the comparison of spin dynamics estimations of spin relaxation

between BRW and SSE theory. Additionally, it is known that the lifetime of radical pairs interacting with the geomagnetic field would require a lifetime of 1 μs [5].

In the context of BRW theory, due to its approximations, the interplay between values of correlation times for spin relaxation effects and the dynamics of the radical pair are of mandatory importance. For instance, if the correlation times increase in relation to the dynamics of $\hat{\rho}(t)$, the positivity of the density matrix is not guaranteed due to the perturbative treatment and erratic expectation values will be calculated [6, 7]. Thus, shortening lifetimes of a radical pair with constant correlation times may lead to erratic behavior in the BRW equations. Because of this, the behavior at around $k_S = 1\ \mu\text{s}^{-1}$ as shown in Fig. S6 should be considered for comparison of BRW and SSE. On the other hand, to calculate longer lifetimes using SSE, the correction factor approximation has to be used when only 550 ns MD data is available. With a rate constant of $k_\text{S} = 1\ \mu\text{s}^{-1}$ a radical pair with a lifetime of 1 μs - a quantity around 2 times longer than the total trajectory time - needs to be considered.

Hence, it is mandatory to confirm whether the total time of 550 ns is enough and what sort of error is to be expected. A model four nuclear spin system, which comprised N5, N10 for the FAD radical and Hβ_1, Hβ_2 for the TrpH radical, described and labeled in Sec. S5.2 is used. Firstly, the symmetric recombination rate is set to $k_\text{S} = 10\ \mu s^{-1}$ - chosen so that the 550 ns trajectory capture converged singlet yield values for this system without the use of the heuristic. $|\mathbf{B}| = 1$ mT, the magnetic field aligned with the z-axis. The direct method was used which gives accurate results without any approximations and a time-step of $dt = 0.5$ ns. The results are shown in Fig. S4.

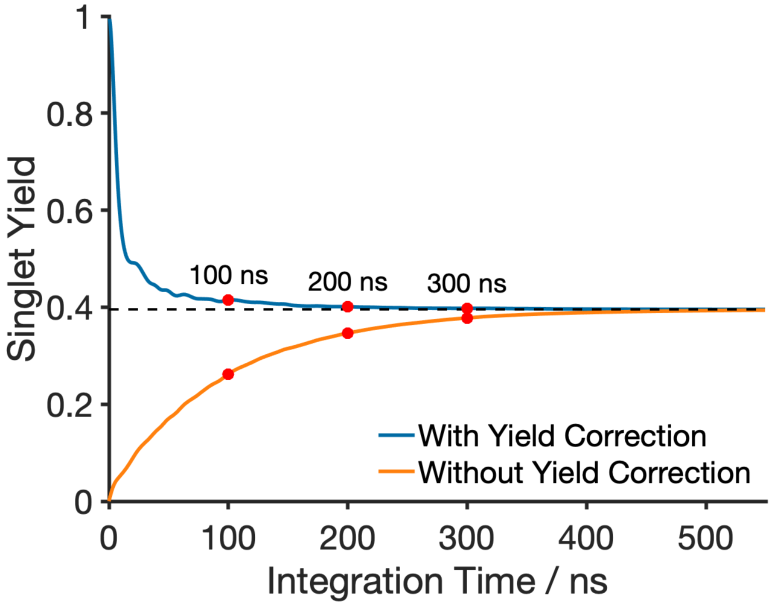

Figure S4: Singlet Yield of $\left[\mathrm{FAD}^{\bullet-}\ \mathrm{TrpH}^{\bullet+}\right]$ radical pair model, Φ_S, versus integration time, T, graphs for 4 nuclear spin radical pair system that had time-dependent interaction from MD trajectory of 550 ns.

In Fig. S4, the labeled red dots reveal that an integration time of $T = 200$ ns is suitable to model the spin system accurately. Since the lifetime was equal to $\tau_{\mathrm{Total}} = 100$ ns, our rule of thumb described before still holds and you would need an integration time that is around 2 times the lifetime of the radical pair to accurately model this system with the heuristic. Besides that, for an integration time that is equal to the lifetime, the results are close to converged values yet a minor error in the quantum yield can be observed. For an integration time that is 3 times as large as the lifetime (300 ns), the singlet yield is clearly fully converged without any errors.

In conclusion, for the spin system presented here, integration times that are around 2 times longer than the lifetime would be sufficient to model singlet yield values (similar to the time-independent case). If the total time is 550 ns, this means a system with a lifetime of 275 ns could be confidently modeled. Hence, the symmetric recombination rate for this lifetime is $k_S \approx 3.64\ \mu\mathrm{s}^{-1}$, and k_S can be $k_S \geq 3.6\mu\mathrm{s}^{-1}$.

Here, only the $k_S = 10\ \mu\mathrm{s}^{-1}$ scenario is considered and it is questionable whether estimating a system's dynamics for a longer lifetime would be appropriate. However, it can be assumed that in the first approximation the frequency of motions and their perturbation

strength would not change significantly after 550 ns. Hence, if the interactions would not change drastically, then using the here presented heuristic to yield results for longer lifetimes can be applied.

S2 Accuracy of the Stochastic Method

In order to evaluate the accuracy of the Monte Carlo (MC) sampling, the standard deviation σ of the singlet yield was calculated for N MC-sampled initial nuclear spin configurations:

$$\sigma = \sqrt{\frac{1}{N-1}\sum_{i=1}^{N}(\Phi_{\mathrm{S}i} - \mu)^2},$$

where μ is the mean singlet yield and $\Phi_{\mathrm{S}i}$ is the singlet yield for the i-th MC sample. The data are assumed to follow a normal distribution. Calculations are performed for various systems containing nuclear spin numbers ranging from 10 to 16 to examine their statistical error. The selected systems are models for the $\left[\mathrm{FAD}^{\bullet-}\mathrm{TrpH}^{\bullet+}\right]$ radical pair, chosen due to their previous analysis. Each system includes at least the following 10 nuclear spins: N5, N10, H6, H8_1, H8_2, H8_3, Hβ_1 for FAD, and N1, H1, H2 for TrpH. For each additional spin, the following hyperfine constants and nuclei are added in direct order: H4, H6, H7, Hβ_1 for TrpH, and Hβ_2, H7_1 for FAD. The hyperfine interaction parameters are listed in Ref. [2]. The symmetric recombination rate is set to $k_S = 1\,\mu\mathrm{s}^{-1}$. Only one MC sample, $M = 1$, is used to observe how the error scales with Z. The heuristic for shortening integration time is applied, with the total time estimated by $\epsilon = 0.5$. The time step is set to 4 ns, and the magnetic field is directed along the z-axis, with a strength of $|\mathbf{B}| = 1\,\mathrm{mT}$. It is important to note that the standard deviation values presented in the Table are calculated using 500 simulations per spin system, each of which used a single SU(Z) state (M = 1).

For each data set, the mean, μ, and standard deviation, σ, are determined, and all values are presented in Table S1.

No. of Nuclear Spins	R	μ	σ
10 spins	13824	0.39480	0.00216
11 spins	27648	0.38853	0.00140
12 spins	55296	0.38546	0.00102
13 spins	110592	0.38290	0.00070
14 spins	221184	0.34984	0.00046
15 spins	442368	0.34706	0.00031
16 spins	884736	0.34661	0.00022

Table S1: Properties of systems with varying numbers of nuclear spins. R denotes the size of the full spin space, μ represents the mean, and σ is the standard deviation of 500 MC samples.

Selected probability distributions, drawn with σ values from Table S1, are depicted in Fig. S5. It is evident that the error diminishes, and the error distribution narrows, as the size of the spin space increases. This result is in excellent agreement with the theoretical evaluation of Weisse et al. [8], which concluded that the relative error of the trace estimation scales as $\mathcal{O}(1/\sqrt{(}MZ))$. Therefore, it can be concluded that with $M = 1$, the errors are sufficiently small for large systems, those with more than 14 nuclear spins, for most applications.

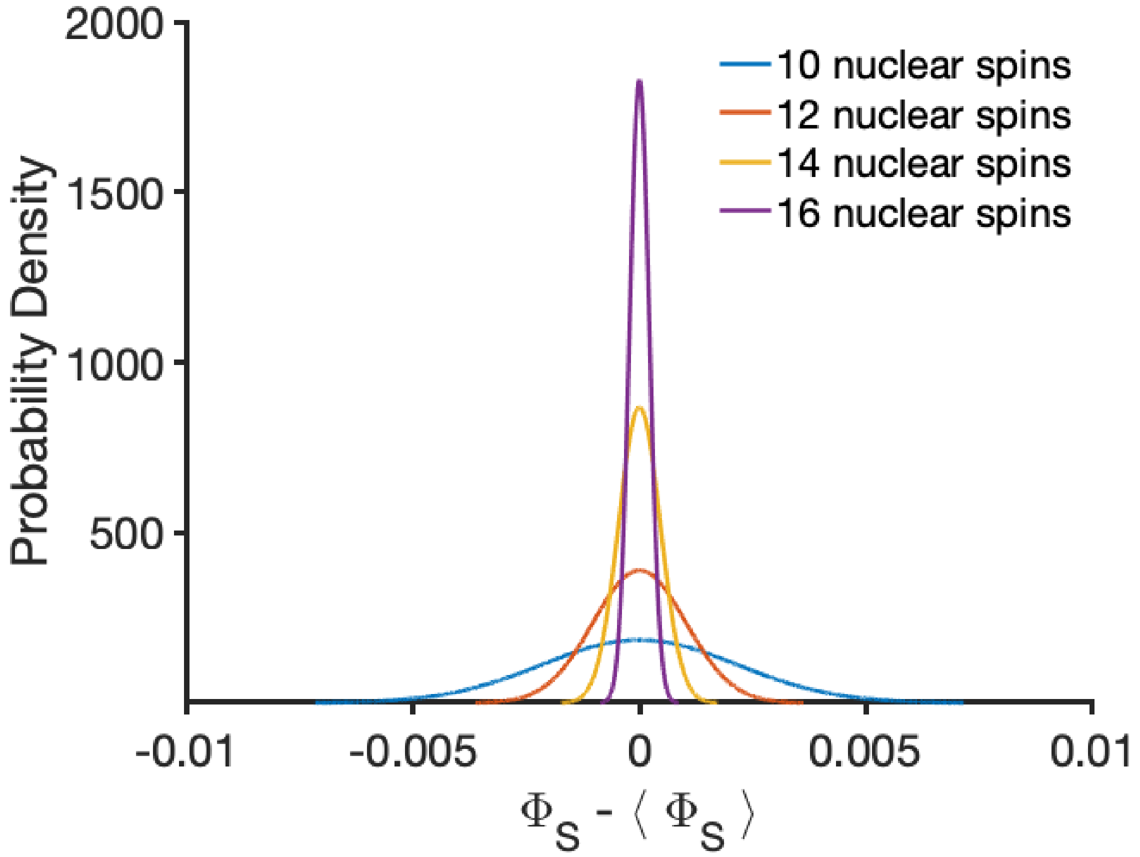

Figure S5: Fitted probability density functions (assuming a normal distribution) for $\Phi_S - \langle \Phi_S \rangle$ values for arbitrary radical pair systems with different numbers of nuclear spins. Here, $\langle \Phi_S \rangle$ denotes the mean singlet yield, also represented as μ. The effect of increasing number of nuclear spins is a decrease in error and a narrowing of the probability density, a phenomenon well established in theory [9].

S3 AutoExpm Method

The AutoExpm [10] method is, to our knowledge, a new implemented algorithm in terms of radical pair spin dynamics for computing the action of a matrix exponential on a vector (or block vectors). The method was introduced by Al-Mohy et al. [10] who found it to be superior to the Chebyshev polynomial [11–13] method or the Krylov subspace method (which is also available for the MolSpin SSE method [14]). It is based on the scaling and squaring method that is usually used to compute full matrix exponentials and computes the action of the matrix exponential as [10]:

$$e^{-i\hat{H}\delta t}B \approx (T_m(s^{-1}(-i\hat{H}\delta t)))^s B \tag{S13}$$

Here s is an chosen integer ≤ 1 [10], T_m is truncated m-term Taylor series:

$$T_m(\hat{A}) = \sum_{j=0}^{m} \frac{\hat{A}^j}{j!}. \tag{S14}$$

The trunctation m of the Taylor series and the parameter s are evaluated by *MolSpin* as described in the next section (see Manual [15]).

B is a block of n state vectors:

$$B = \Big[|\Psi_1(t)\rangle \quad |\Psi_2(t)\rangle \quad \ldots \; |\Psi_n(t)\rangle \Big] \tag{S15}$$

A more detailed derivation can be found in Ref. [10]. The method described in the section below uses a few key ideas to make this computation as efficient as possible. It carefully estimates parameters m and s to ensure that the backward error is suitably bounded while the computational cost is minimized, it does shifting to reduce the norm of A (which reduces the cost) and terminates prematurely when a certain accuracy is reached. All of these feature combined makes a unique algorithm that has several key advantages over Krylov Subspace algorithms:

1. It can handle multiple columns in B.

2. There are no parameters to be estimated and the algorithm chooses the best ones itself.

3. It can be quicker than Krylov methods in some cases.

4. Predictable cost of the method. Also cost tends to increase with increasing $||\hat{A}||_1$. Whereas for Krylov methods there is only some dependence with $||\hat{A}||_1$.

We will describe the algorithm below which we named *AutoExpm*.

S3.1 *AutoExpm* Algorithm

The main algorithm is in Alg. 1. It uses $\hat{A}$, δt, B and tolerance *tol* as an input to solve $\exp(-i\hat{A}\delta t)B$. $\hat{A}$ will be $\hat{H}$ or $\hat{H} - i\hat{K}$. The tolerance will determine how accurate the

approximation will be and there are three choices of double, single or half digit precision. $||\cdot||_\infty$ in the algorithm itself is the infinity-norm.

Algorithm 1: AutoExpm method for computing the action of the matrix exponential

$\mu = \mathrm{Tr}(\hat{A})/n$

$\hat{A} = \hat{A} - \mu\mathcal{I}$

$[m_*, s] = parameters(-iA\delta t, tol)$

```
// The function is in code Fragment in Alg.  2
```

$F = B$

$\eta = e^{t\mu/s}$

for $i = 1 : s$ **do**

    $c_1 = ||B||_\infty$

    for $j = 1 : m_*$ **do**

        $B = -i\hat{A}B\delta t/(sj)$

        $c_2 = ||B||_\infty$

        $F = F + B$

        if $c_1 + c_2 \leq tol||F||_\infty$ **then**

            break

        $F = \eta F$

        $B = F$

The algorithm that estimates optimal parameters $s, m_*(= m$ in Eq. S14) is shown in Alg. 2. In the algorithm, $||\cdot||_1$ is the 1-norm, $\lceil\cdot\rceil$ is the ceiling function, $argmin(\cdot)$ finds the minimum given different arguments and $max(\cdot)$ computes the maximum from given inputs. Besides the algorithm, there a few extra equations used for parameter estimation. First, the equation for optimal cost for a given $\hat{A}$:

$$C_{m_*}(\hat{A}) = min\left\{m\lceil\alpha_p(\hat{A})/\theta_m\rceil : 2 \leq p \leq p_{max}, p(p-1) - 1 \leq m \leq m_{max}\right\} \tag{S16}$$

Here p_{max} and m_{max} are maximum parameters for m and p. They are suggested by the authors of the algorithm from experience as $m_{max} = 55$ and $p_{max} = 8$. Also, $\alpha_p(\hat{A})$ is defined as :

$$\alpha_p(\hat{A}) = max(d_p, d_{p+1}),\ d_p = ||\hat{A}^p||_1^{1/p} \tag{S17}$$

θ_m are a number of parameters for different values of m that are used for parameter estimation. They are linked to the tolerance of the method and have different values if either one of *double*, *single* and *half* tolerance is used. Selected constants for θ_m are shown in Table S2. Lastly, the algorithm for parameter estimation also uses this bound on $||\hat{A}||_1$. Here n_0 is the number of rows and l is the number of columns in B.

$$||\hat{A}||_1 \leq 2\frac{l}{n_0}\frac{\theta_{m_{max}}}{m_{max}}p_{max}(p_{max}+3) \tag{S18}$$

Algorithm 2: Parameter estimation for autoexpm method

Given: tol, m_{max}, p_{max}
if *If Eq. S18 is satisfied* **then**
    $m_* = argmin_{1\leq m\leq m_{max}} m\lceil ||\hat{A}||_1/\theta_m \rceil$
    $s = \lceil ||\hat{A}||_1/\theta_m \rceil$
else
    Let m_* be the smallest m achieving the minimum in Eq. S16
    $s = max(C_{m_*}(\hat{A})/m_*, 1)$

m	5	10	15	20	25	30	35	40	45	50	55
half	8.1×10^{-1}	2.3	3.8	5.3	6.8	8.2	9.7	1.1×10^{1}	1.3×10^{1}	1.4×10^{1}	1.5×10^{1}
single	2.3×10^{-1}	1.0	2.2	3.6	4.9	6.3	7.7	9.1	1.1×10^{1}	1.2×10^{1}	1.3×10^{1}
double	2.4×10^{-3}	1.4×10^{-1}	6.4×10^{-1}	1.4	2.4	3.5	4.7	6.0	7.2	8.5	9.9

Table S2: Selected constants θ_m for tolerances of half ($tol = 2^{-10}$), single ($tol = 2^{-24}$) and double ($tol = 2^{-53}$) precisions.

A more in depth discussion of the theory behind the algorithm can be found in Ref. [10]. The an open-source implementation of a more general method of the algorithm can be found here:

https://github.com/higham/expmv

S4 Implemented Tasks and Perfomance in *MolSpin*

Table S3 illustrates the available tasks and their respective perfomance as currently implemented in *MolSpin*. Different methologies are available for time-independent and time-dependent Hamiltonians. The memory usage scales with $\mathcal{O}(Z\log(Z))$ in all tasks. For more information we refer to the user manual which can be found at Ref. [15].

Table S3: Available tasks and properties in *MolSpin* to use the SSE method. The tasks are divided into two Hilbert Space (**HS**) regimes: **StaticHS** (time-independent Hamiltonian) and **DynamicHS** (time-dependent Hamiltonian). Furthermore, direct (**Direct**) and stochastic (**Stoch**) methods are available for quantum yields (**Yields**) and pure time-evolution of spin states (**TimeEvo**). **Type:** describes what can be calculated, which is either the time-evolution (**TE**) or quantum yields (**QY**). **TD** (**Time-Dependency**): Describes if the method can be used with time-dependent interactions. All of the methods utilize Haberkorn reaction operators for describing radical reactions. **Speed:** describes the performance with M as the number of Monte Carlo samples and Z as the size of nuclear spin subspace. **Error**: describes the error in the final results of simulations. *Exact* means that there are no errors in calculations and the scaling was given only for SU(Z) spin states. In the case of coherent spin states, the scaling would be $\mathcal{O}(1\ \sqrt{M})$.)

Task class	Type	TD	Speed	Error
**StaticHS-StochYields**	QY	No	$\mathcal{O}(N_t MZ\log(Z))$	$\mathcal{O}(1/\sqrt{MZ})$
**StaticHS-StochTimeEvo**	TE	No	$\mathcal{O}(N_t MZ\log(Z))$	$\mathcal{O}(1/\sqrt{MZ})$
**StaticHS-DirectYields**	QY	No	$\mathcal{O}(N_t Z^2\log(Z))$	Exact Method
**StaticHS-DirectTimeEvo**	TE	No	$\mathcal{O}(N_t Z^2\log(Z))$	Exact Method
**DynamicHS-StochYields**	QY	Yes	$\mathcal{O}(N_t MZ\log(Z))$	$\mathcal{O}(1/\sqrt{MZ})$
**DynamicHS-StochTimeEvo**	TE	Yes	$\mathcal{O}(N_t MZ\log(Z))$	$\mathcal{O}(1/\sqrt{MZ})$
**DynamicHS-DirectYields**	QY	Yes	$\mathcal{O}(N_t Z^2\log(Z))$	Exact Method
**DynamicHS-DirectTimeEvo**	TE	Yes	$\mathcal{O}(N_t Z^2\log(Z))$	Exact Method

S5 Comparison of SSE Method with Bloch-Redfield-Wangsness Theory using 550 ns MD trajectory

In recent years, various theoretical approaches have been employed to investigate the impact of spin relaxation of radical pairs in biological systems [6, 7, 16, 17]. One approach gaining considerable attention is the phenomenological Lindblad operator, owing to its ease of application. It primarily requires relaxation rates, which can be derived from experimental data or theoretical computations [6]. However, these methods often lack a detailed microscopic physical representation of the actual movements and magnetic interactions of the radicals.

An alternative approach, called Bloch-Redfield-Wangsness (BRW), provides a quantum master equation characterizing the interaction between a spin system and its environment as a perturbation introduced through stochastic functions [6, 16, 18]. Furthermore, it can include time-dependent trajectories generated by the previously mentioned multiscale approach. The theory is derived by splitting the Hamiltonian $\hat{H}$ into a system part $\hat{H}_{\mathrm{S}}$ and an environment part $\hat{H}_{\mathrm{Env}}$ coupled by a time-dependent interaction Hamiltonian $\hat{H}_{\mathrm{I}}(t)$:

$$\hat{H} = \hat{H}_{\mathrm{S}} + \hat{H}_{\mathrm{Env}} + \hat{H}_{\mathrm{I}}(t). \tag{S19}$$

The goal of BRW theory is to neglect the explicit description of the environment dynamics, $\hat{H}_{\mathrm{Env}}$, focusing instead on the spin dynamics of the system. It modifies $\hat{H}_{\mathrm{I}}$ in a manner that allows all time-dependence to be described by a simpler stochastic coupling function.

BRW is based on three assumptions, namely:

1. Assuming that the ensemble-averaged expectation value of the interaction operator $\hat{H}_{\mathrm{I}}$ is zero.

2. The environment of the spin system is unaffected by the dynamics of the spin system.

3. The time-dependent perturbation has a significantly lower amplitude than the static Hamiltonian $\hat{H}_{\mathrm{S}}$ [18].

Several writings detail the full derivation of the BRW master equation [6, 7, 16, 18]. The final equation of motion to solve can be written in Liouville space as:

$$\frac{d\hat{\rho}(t)}{dt} = -i\hat{\hat{H}}_{\mathrm{S}}\hat{\rho}(t) - \hat{\hat{\mathcal{K}}}\hat{\rho}(t) + \hat{\hat{R}}_{RF}\hat{\rho}(t) = \hat{\hat{L}}\hat{\rho}(t), \tag{S20}$$

where $\hat{\hat{R}}_{RF}$ denotes the Redfield relaxation superoperator, which can be formally formulated in the interaction picture (subscript I) as:

$$\begin{aligned}\hat{\hat{R}}_{RF}\hat{\rho}_I(t) = -\sum_{\alpha,\beta}\int_0^{\infty} & d\tau g_{\alpha,\beta}(\tau)[\hat{A}_{I,\alpha}(t), \hat{A}_{I,\beta}(t-\tau)\hat{\rho}_I(t)] \\ & - g^*_{\alpha,\beta}(\tau)[\hat{A}_{I,\alpha}(t), \hat{\rho}_I(t)\hat{A}_{I,\beta}(t-\tau)],\end{aligned} \tag{S21}$$

where $\hat{A}_{I,\alpha}(t)$ are spin system operators (i.e. $\hat{S}_{1x}$, $\hat{S}_{1y}$), and $g_{\alpha,\beta}(\tau)$ is a correlation function condensing the fluctuations of the environment with the lag time τ as:

$$g_{\alpha,\beta}(\tau) = \langle b_\alpha(t)|b_\beta(t+\tau)\rangle, \tag{S22}$$

with $b_\alpha(t)$ being the coupling parameters of interaction that are modulated by stochastic fluctuations and defined through the stochastic part of the Hamiltonian:

$$\hat{H}(t) - \hat{\hat{H}} = \sum_\alpha b_\alpha(t)\hat{A}_\alpha. \tag{S23}$$

Here, $\hat{\hat{H}}$ is the static part of the Hamiltonian, from which an eigenbasis is used to bring Eq. S21 into an explicit form. The correlation function can be transformed into spectral densities $J_{\alpha\beta}(\omega_{mn})$ [6]:

$$J_{\alpha\beta}(\omega_{mn}) = \int_0^{\infty} d\tau g_{\alpha,\beta}(\tau)e^{i\omega_{mn}\tau}, \tag{S24}$$

where $\omega_{mn} = \omega_m - \omega_n$ are the eigenvalue differences between states $|m\rangle$ and $|n\rangle$ of the static Hamiltonian [6, 16].

It is assumed that the correlation function decays rapidly in an exponential form,

allowing the solution of the spectral density integral as:

$$J(\omega) = g(0)\frac{1}{\frac{1}{\tau} - i\omega} = g(0)\left(\frac{\tau}{1+\omega^2\tau^2} + \frac{i\omega\tau^2}{1+\omega^2\tau^2}\right), \tag{S25}$$

with $g(0)$ being the amplitude of the correlation function. Kattnig *et al.*[6, 16] showed that the correlation function of complex situations, such as the motion of atoms in proteins, cannot be accurately described by a single exponential correlation function. A more realistic description is achieved by constructing the spectral density from a linear combination of correlation functions:

$$J(\omega) = \sum_q g_q(0)\frac{1}{\frac{1}{\tau_q} - i\omega}, \tag{S26}$$

This formalism can now be used to describe the induced spin relaxation explicitly within the BRW theory using Molecular Dynamics (MD) data, which employs correlation functions to capture stochastic fluctuations. A comparison of this approach with the SSE formalism is made to evaluate the advantages and limitations of both methods in describing spin relaxation.

A simplified spin system of a radical pair within a cryptochrome protein from the European robin, coupled to the N5 nucleus of the FAD, is used for comparison (see Fig. S6a). To describe spin relaxation effects through motion similar to previous works, specific degrees of freedom were extracted from a MD simulation. These are thought to significantly influence the spin dynamics of the radical pair. Earlier studies found that the librational motion of the $FAD^{\bullet-}$ radical and fluctuations in the dihedral angle Ω (Fig. S6a, green) have a significant effect on the hyperfine coupling of nuclei, which reduces the anisotropy of the singlet yield [16].

The geometry of $FAD^{\bullet-}$ was extracted from 550 ns (500 fs time steps) taken from the MD trajectory (the data were provided by Gesa Grüning [19]). The hyperfine tensors for selected motions were calculated using density functional theory (B3LYP/EPR-II) employing Gaussian 09 [20]. The hyperfine tensors calculated for specific dihedral angles and librational angles are mapped to the frames of the MD trajectory to produce a time-

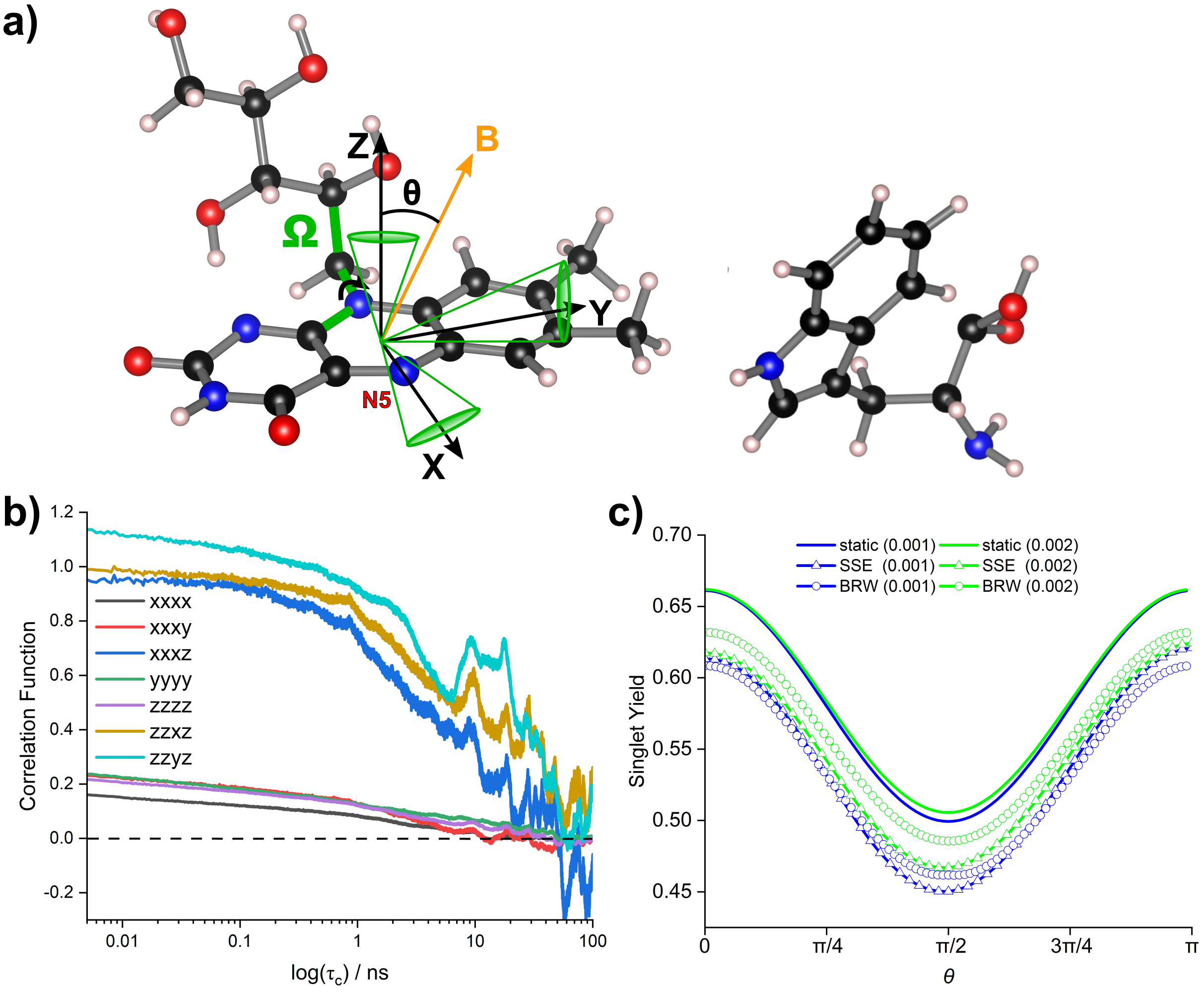

Figure S6: **a)** Schematic representation depicting the included nucleus N5, the dihedral angle Ω, and librational motion around all axes of FAD (green). The magnetic field $|\mathbf{B}|$ (50 μT) is rotated in the xz-plane through an angle θ. The radical pair of $\left[\text{FAD}^{\bullet-}\ \text{TrpH}^{\bullet+}\right]$ is initialized in a singlet state. The N5 nucleus is included to model the motion-modulated hyperfine interaction, with the hyperfine tensor rotated in the eigenframe of the N5 nucleus. The static part of the hyperfine tensor is chosen to be the average of all frames within the MD trajectory; all parameters are taken from Grüning *et al.* [19]. **b)** Selected correlation functions for elements in the N5 hyperfine coupling tensor extracted from a 550 ns MD trajectory. Notably, cross-terms including z-components exhibit the strongest correlation, a well-known phenomenon in FAD due to the hyperfine coupling primarily occurring perpendicular to the FAD plane (z-axis) [6, 16, 19]. **c)** A comparative analysis of the static singlet quantum yield Φ_S of the radical pair. The SSE method has an explicit description of fluctuations over a 550 ns trajectory and BRW results incorporating 81 correlation functions constructed from the nine tensor elements. Two different reaction rate constants (found in brackets and in ns^{-1}) were chosen. For the slower reaction rate constant (0.001 ns^{-1}) the SSE method uses the correction factor described in the main text.

dependent trajectory of the hyperfine tensor under the influence of these two specific motions. The procedure is discussed in detail by Kattnig *et al.* [6, 16]. The amplitudes

$g(0)_q$ and the corresponding correlation times τ^q can be directly used in *MolSpin*, which computes the spectral density automatically [6].

Fig. S6b illustrates exemplary correlation functions of the 3x3 hyperfine tensor in the nucleus principal axis system. The mean of the hyperfine coupling elements was subtracted (see Eq. S23) to only consider the time-dependent fluctuations. As observed, cross-correlations, such as $xxxz$ (Fig. S6b), which incorporate coupling components with z-axis character, are significant and must be considered in the spin relaxation procedure, as previously described by Kattnig *et al.*[16]. The strong fluctuation of the N5 hyperfine tensor in the z-direction (orthogonal to the $FAD^{\bullet-}$ plane) is a known feature [6], also found in the N10 nucleus [6, 16]. Furthermore, it can be observed (i.e. for $zzyz$) that the 550 ns MD trajectory might be to short for smoothly decaying correlation function fits which may lead to inaccuracies in the fitting procedure for BRW theory. In general, longer MD trajectories are required to accurately describing the spin relaxation effects [17]. For the fitting procedure of these correlation functions, 50 exponential functions (logarithmically spaced lag times τ from 0.005 to 300 ns) were taken to extract the amplitudes $g(0)$. These amplitudes are then modulated by the covariance of the two involved coupling elements. The modified amplitudes and lag times τ are used for each of the 81 relaxation terms to construct the Redfield tensor.

For the explicit description of the time-dependent fluctuation of the N5 hyperfine coupling tensor in the framework of SSE, the 1,100,000 frames were averaged over consecutive 5-ns intervals, using the average hyperfine tensor for each 0.5 ns time frame. It was found that faster motions do not drastically influence the spin dynamics and can be neglected here (see Section S5.1). Furthermore, for lifetimes longer then 550 ns the previously discussed quantum yield correction for integration was used due to the absence of longer MD data.

In both the BRW and SSE scenarios, the static component of the N5 hyperfine coupling with one electron is averaged over the entire MD trajectory, and calculations are performed in the N5 nucleus eigenframe. The radical pair originates in a singlet state, with reaction rate constants k_{S} and k_{T} set to 0.001 and 0.002 ns^{-1}, respectively. The magnetic field,

$|\mathbf{B}|$, is set to 50 μT, initially parallel to the z-axis and then rotated 180° around the y-axis, as illustrated in Fig. S6a.

Fig. S6c compares the singlet quantum yields of both relaxation methods (circles = BRW, triangles = SSE) against the static spin dynamics (solid lines). It is evident that spin relaxation decreases the overall quantum yield compared to the static scenario in both cases. However, the SSE method induces stronger spin relaxation than the BRW method, particularly at 0.002 ns^{-1}. At a reaction rate constant of 0.001 ns^{-1} (blue curves), both BRW and SSE curves align well, indicating similar relaxation effects.

Despite the sensitivity of the BRW approach to the correlation function fitting procedure and the use of the discussed extrapolation method for SSE in the small reaction rate constant case, the relaxation effects observed are consistent. The SSE approach, as implemented, effectively describes complex spin relaxation mechanisms extracted from multi-scale MD/QM data. Contrary to the BRW approach, it does not treat the magnitude of time-dependent perturbation as a limiting factor for including spin relaxation, resulting in a more versatile model. However, it necessitates longer MD trajectories for slow reaction rate constants (long lifetimes), as evidenced in Section S1. It was demonstrated that to achieve accurate results with the correction factor, integration times must be at least 2.3 times the lifetime. Therefore, the SSE result for a reaction rate constant of 0.001 ns^{-1} should be considered cautiously.

Similarly, the MD trajectory length is pivotal for accurately capturing spin relaxation effects within the BRW theory. It is also important to note that the BRW theory may not be suitable in specific scenarios due to its perturbative-Markovian foundation [6]. Specifically, the theory may become invalid when the spin dynamics of the system becomes faster. In such situations, alternative theories like the Nakajima-Zwanzig approach have been proven to yield superior results [7].

Having successfully compared the SSE approach with another spin relaxation theory using MD data, it is now possible to investigate the influence of spin relaxation on cryptochromes for a larger number of nuclei. For this, again, the MD trajectory of Grüning *et al.* for the motion of dihedral and librational angles is used [19]. Figure S7 illustrates

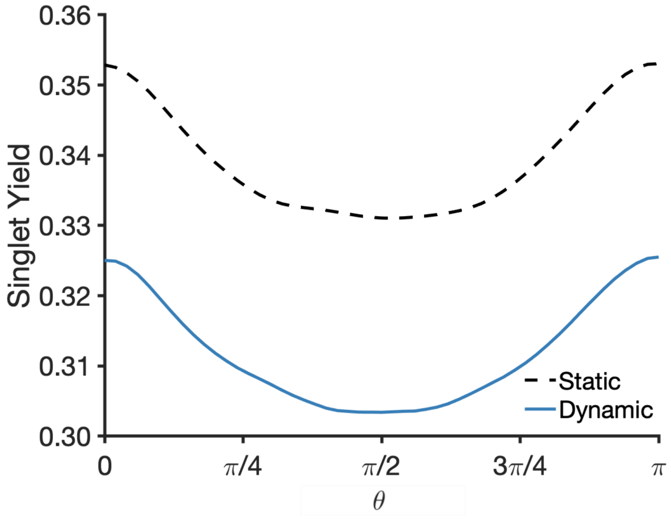

Figure S7: Quantum yield of a $\left[\mathrm{FAD}^{\bullet-}\ \mathrm{TrpH}^{\bullet+}\right]$ radical pair with 14 nuclear spins calculated using the SSE method with and without spin relaxation due to motion-modulation of hyperfine interactions. Similar parameters for the calculation set up as illustrated in Fig. S6 are employed. For the static part of the hyperfine interactions, the average of the time trajectory extracted from MD (550 ns) simulations was used for each nucleus. The reaction rate constants were assumed $k_{\mathrm{S}} = 10\mu\mathrm{s}^{-1}$, $k_{\mathrm{T}} = 10\mu\mathrm{s}^{-1}$

.

the singlet quantum yield for the cryptochrome radical system of European robin from FAD and Trp (similar to Fig. S6) when including in total 14 different nuclei. The chosen nuclei and average hyperfine coupling tensors can be found in Fig. S5.2 and are labled according to Ref. [19].

In the simulation presented here, however, a shorter lifetime of the radical pair is used because only 550 ns MD trajectories are available and no correction factor should be used for this example. The example should only serve as an illustration, that the SSE approach can straight-forwardly include many time-dependent interactions using *.mst*-files extracted from MD/QM calculations. Thus, the reaction rate constants of $k_{\mathrm{S}} = 10\mu\mathrm{s}^{-1}$, $k_{\mathrm{T}} = 10\mu\mathrm{s}^{-1}$ are chosen. Additionally, the applied magnetic field was set to 1 mT which in alignment with the new lifetime of the radical pair to observe a magnetic field effect. For all hyperfine tensors spin relaxation is considered similar to the N5 case in Fig. S6.

Similar to previous work, the overall anisotropy of the singlet quantum yield decreases (Fig. S7 static) when including more nuclei to the spin system [19]. After the consideration of motion-induced spin relaxation through all hyperfine interactions, the overall singlet

quantum yield drops significantly. Thus, spin relaxation still plays a major role when dealing with big spin systems and MD trajectories can be used to describe the complex relaxation mechanisms within a protein

To provide more insight into this feature, we will investigate a radical pair of FAD and Trp within the Eurpean robin cryptochrome using 1 μs MD simulations to not only consider two degrees of freedom such as illustrated here, but all of the motions perturbing the HFI. Furthermore, dipolar coupling will be included, as its importance was stated previously.

S5.1 Selection of Time Step for Molecular Dynamics Data (550 ns)

The MD data for the $\left[\mathrm{FAD}^{\bullet-}\ \mathrm{TrpH}^{\bullet+}\right]$ radical pair in a protein environment, was obtained with a time step of 500 fs. Incorporating all of these frames into spin dynamics calculations is both resource-intensive and unnecessary. It is crucial to select a time step that adequately captures all significant motions affecting the spin dynamics of a radical pair with a lifetime on the order of 1μs or several orders of magnitude more or less. Hence, this section analyses the appropriate choice of a time-step for this system to yield accurate results.

This requisite time step was estimated by calculating singlet yield graphs for a four spin system as described in a previous section (Section S1) with varying time steps for the fluctuation of hyperfine parameters. The system included N5, N10 hyperfine interactions for the FAD radical, and $H\beta_1$, $H\beta_2$ hyperfine interaction for the TrpH radical. The labels and average hyperfine interactions of these atoms are provided in Section SS5.2. In the static case, averaged hyperfine interactions are used, while in the dynamic case, the hyperfine interaction parameters were time-dependent and extracted from MD trajectories. The symmetric recombination rate constant was set to $k_\mathrm{S} = 10\,\mu\mathrm{s}^{-1}$, and the magnetic field strength was $|\mathbf{B}| = 1\,\mathrm{mT}$, with no coupling terms between electron spins. The total simulation time was 550 ns, sufficient to sample the singlet yield values of a radical pair with a 100 ns lifetime. The direct method, which provides singlet yield values without any approximations, was employed. The selected time step values were

$dt = 0.5\,\text{ns}$; $0.1\,\text{ns}$; $0.05\,\text{ns}$; $0.01\,\text{ns}$. The graphs for the static and various dynamic cases are depicted in Fig. S8.

It is concluded that $dt = 0.5\,\text{ns}$ is adequate for modelling this MD data applied to the spin dynamics of the radical pair. To confirm this, a 14-nuclear spin system was also calculated, incorporating all hyperfine interactions to examine the time-dependent effects on the singlet yield.

This 14-nuclear spin radical pair is described in Section S5.2. The static case utilize the average hyperfine interactions outlined in that section, while the dynamic case involve all 14 nuclei following the trajectory of the MD data. Different time steps are employed to model the data, with the symmetric recombination rate set to $k_{\text{S}} = 10\,\mu\text{s}^{-1}$, the magnetic field strength at $|\mathbf{B}| = 1\,\text{mT}$, and no coupling terms between electron spins. The total simulation time is $550\,\text{ns}$. The stochastic method is used, with $MC = 5$ states employed for calculating dynamics. The results are displayed in Fig. S9. For the illustrated scenario as well, $dt = 0.5\,\text{ns}$ is found to be sufficient for modelling the system. Therefore, a time step of $dt = 0.5\,\text{ns}$ will be used for calculations involving the 550 ns MD trajectory.

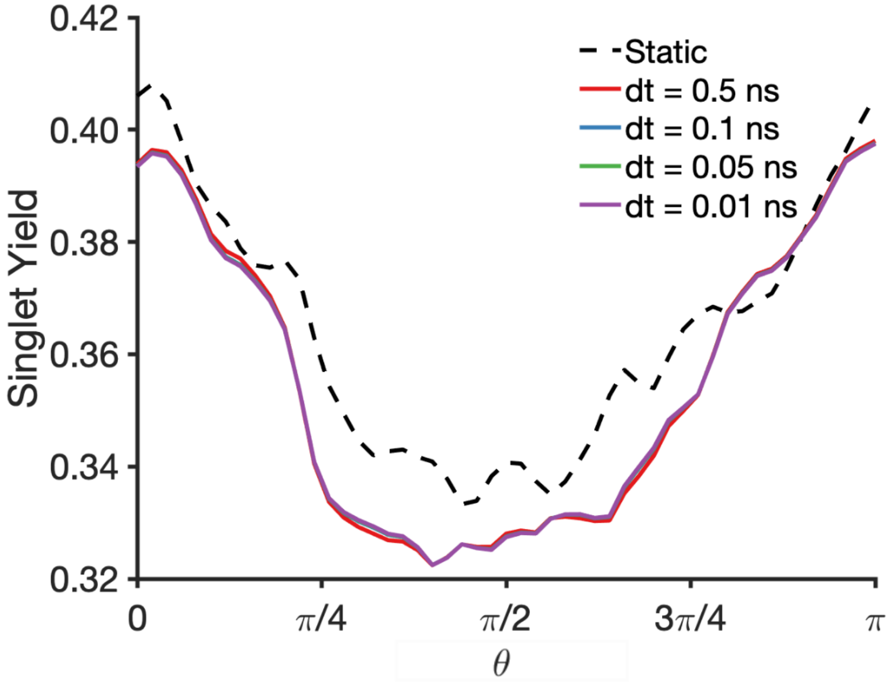

Figure S8: Singlet Yield of a $[FAD^{\bullet -}\ TrpH^{\bullet +}]$ radical pair including 4-nuclear spins vs. angle θ of the external magnetic field. Parameters used in the calculation are described in the text of Section S5.1. The static case without time-dependent interactions in $\hat{H}(t)$ is represented by the black dashed line, while the other lines correspond to interactions that varied with different time step dt values.

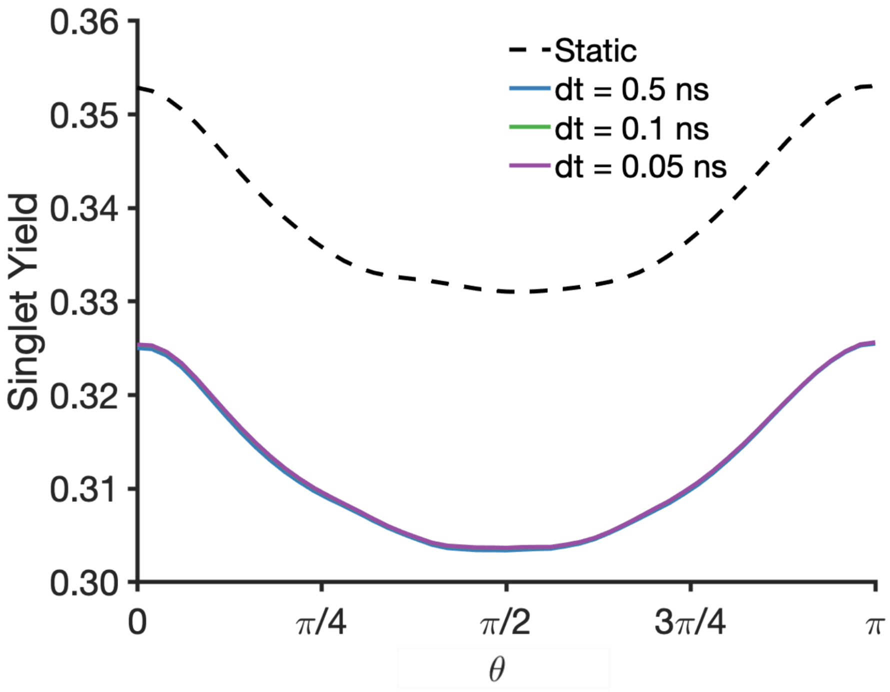

Figure S9: Singlet Yield of a $\left[\mathrm{FAD}^{\bullet-}\ \mathrm{TrpH}^{\bullet+}\right]$ radical piar including 14-nuclear spins vs. angle θ of the external magnetic field. Parameters used in the calculation are described in the text of Section S5.1. The static case without time-dependent interactions in $\hat{H}(t)$ is represented by the black dashed line, while the other lines correspond to interactions that varied with different time step dt values.

S5.2 Averaged Hyperfine Interactions for MD calculations (550 ns)

In this section, we present averaged hyperfine interaction tensors that were constructed from molecular dynamics data. The hyperfine data for the $\mathrm{FAD}^{\bullet-}$ radical is presented in Table S4 and data for $\mathrm{TrpH}^{\bullet+}$ is presented in Table S5. Structures of both of these radicals together with atom labels are shown in Fig. S10.

Table S4: Averaged hyperfine tensors for the $FAD^{\bullet -}$ radical. Data and labelling were taken from a 550 ns molecular dynamics trajectory of the European Robin cryptochrome 4a protein [19]. Each hyperfine tensor was rotated to the frame of N5 of $FAD^{\bullet -}$. $FAD^{\bullet -}$ structure and labels of each atom can be seen in Fig. S10.

Atom	Hyperfine Tensor **A** / mT
N5	$\begin{pmatrix} -0.0936 & 0 & 0 \\ 0 & -0.0840 & 0 \\ 0 & 0 & 1.8807 \end{pmatrix}$
N10	$\begin{pmatrix} 0.0221 & -0.0120 & -0.0812 \\ -0.0120 & 0.0164 & 0.0479 \\ -0.0812 & 0.0479 & 0.6850 \end{pmatrix}$
H6	$\begin{pmatrix} -0.2519 & -0.1238 & 0.0014 \\ -0.1238 & -0.4087 & -0.0004 \\ 0.0014 & -0.0004 & -0.4090 \end{pmatrix}$
$H\beta_1$	$\begin{pmatrix} 0.1212 & -0.0575 & 0.0308 \\ -0.0575 & 0.1564 & -0.0391 \\ 0.0308 & -0.0391 & 0.0823 \end{pmatrix}$
$H8_1$	$\begin{pmatrix} 0.5918 & 0.0302 & 0.0110 \\ 0.0302 & 0.6695 & 0.0258 \\ 0.0110 & 0.0258 & 0.5890 \end{pmatrix}$
$H8_2$	$\begin{pmatrix} -0.0047 & -0.0239 & 0 \\ -0.0239 & 0.0727 & 0.0017 \\ 0 & 0.0017 & -0.0078 \end{pmatrix}$
$H8_3$	$\begin{pmatrix} 0.6153 & 0.0292 & -0.0092 \\ 0.0292 & 0.6968 & -0.0247 \\ -0.0092 & -0.0247 & 0.6132 \end{pmatrix}$

Table S5: Averaged hyperfine tensors for the $TrpH^{\bullet+}$ radical. Data were taken from 550 ns molecular dynamics trajectory of the European Robin cryptochrome 4a protein [19]. Each hyperfine tensor was rotated to the frame of N5 of $FAD^{\bullet-}$. $TrpH^{\bullet+}$ structure and labels of each atom can be seen in Fig. S10.

Atom	Hyperfine Tensor $\mathbf{A}$ / mT
$H\beta_1$	$\begin{pmatrix} 0.2784 & -0.1152 & 0.0349 \\ -0.1152 & 0.2505 & -0.0144 \\ 0.0349 & -0.0144 & 0.1536 \end{pmatrix}$
$H\beta_2$	$\begin{pmatrix} 1.2805 & -0.0282 & 0.1112 \\ -0.0282 & 1.2083 & 0.0032 \\ 0.1112 & 0.0032 & 1.3298 \end{pmatrix}$
HD_1	$\begin{pmatrix} -0.8471 & 0.0489 & -0.0942 \\ 0.0489 & -0.2943 & 0.2469 \\ -0.0942 & 0.2469 & -0.5479 \end{pmatrix}$
HE_1	$\begin{pmatrix} -0.3039 & 0.2069 & 0.2781 \\ 0.2069 & -0.6888 & 0.0419 \\ 0.2781 & 0.0419 & -0.3646 \end{pmatrix}$
HE_3	$\begin{pmatrix} -0.3705 & 0.0700 & 0.1894 \\ 0.0700 & -0.7662 & -0.0660 \\ 0.1893 & -0.0660 & -0.5116 \end{pmatrix}$
HH_2	$\begin{pmatrix} -0.4729 & -0.1917 & -0.1287 \\ -0.1917 & -0.2737 & -0.0019 \\ -0.1287 & -0.0019 & -0.5365 \end{pmatrix}$
NE_1	$\begin{pmatrix} 0.0831 & 0.1647 & -0.2668 \\ 0.1647 & 0.0756 & -0.2784 \\ -0.2668 & -0.2784 & 0.3663 \end{pmatrix}$

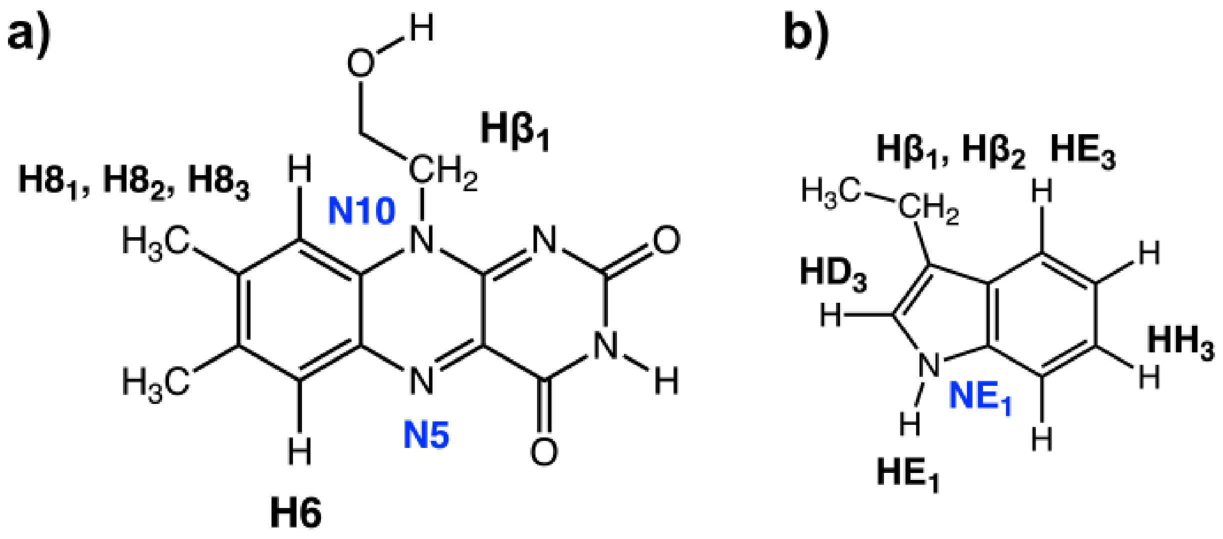

Figure S10: Molecular structures and atomic label convention fo **a)** $FAD^{\bullet -}$ and **b)** $TrpH^{\bullet +}$ radicals according to the MD data of Grüning *et al.* [19].

All of the hyperfine tensors were rotated to the frame of N5 in which the hyperfine tensor of N5 has only diagonal elements by:

$$\mathbf{A}_{Rot} = \mathbf{R}^T \mathbf{A} \mathbf{R} \tag{S27}$$

$\mathbf{R}$ is the matrix of eigenvectors of $\mathbf{A}(N5)$:

$$\mathbf{R} = \begin{pmatrix} 0.8216 & -0.1777 & -0.5417 \\ 0.3158 & 0.9329 & 0.1730 \\ -0.4746 & 0.3132 & -0.8226 \end{pmatrix} \tag{S28}$$

N5 hyperfine tensor before diagonalization was:

$$\mathbf{A}(N5) = \begin{pmatrix} 0.4860 & -0.1866 & 0.8792 \\ -0.1866 & -0.02619 & -0.2782 \\ 0.8792 & -0.2782 & 1.2433 \end{pmatrix} \tag{S29}$$

S6 Time-step Choice for Electronic Structure Calculations of 953 ns MD trajectory

In Sections S5, S5.1, and S5.2 we have analysed the time-dependent hyperfine interactions under the effect of rotational and librational motions induced spin relaxation from Ref. [19] for a 550 ns MD trajectory. These data were required for an accurate comparison with the BRW theory since any other more complicated motion would be challenging and/or impossible to model with the BRW theory.

Nonetheless, to showcase the SSE method's capabilities, we have also produced a longer MD trajectory (with a total time of 953 ns) and used hyperfine interactions in simulations calculated with DFT in each frame. Hence, the simulation with these hyperfine interactions would include all motions, i.e. rotations, translations and vibrations. In contrast, the previous data set had hyperfine interactions that were rotated according to the MD trajectory from a single initial DFT calculation. This section overviews the choice of the time step of the simulation that was sufficient to include all interactions. It is an important parameter to estimate correctly because calculating hyperfine interactions with DFT for each 500 fs frame of the MD trajectory would be too expensive and unnecessary to capture accurate spin dynamics. Hence, we wanted to choose the highest time step that would not compromise the accuracy of the simulations.

We have chosen to simulate the first 10 ns of the MD trajectory with different time steps dt = 500 fs, 1 ps, 5 ps, 10 ps, 50 ps, 100 ps, 200 ps, 500 ps and plot singlet survival probability against time to see at which dt the plots diverge. Simulating 10 ns with dt = 500 fs already required 20000 DFT calculations of both FAD and Trp radicals.

In Fig. S11 we see plots of singlet survival probability versus time for a 4 spin system with different time steps dt. This system included N5, N10 nuclear spins for $FAD^{\bullet -}$ radical, and N1, H1 for $TrpH^{\bullet +}$ radical, as described in Section S7. $|\mathbf{B}| = 1$mT and was aligned along the z-axis. The recombination rate was $k = 10\mu s^{-1}$. Moreover, a time-dependent dipolar interaction was included in the calculations that was computed from the MD trajectory.

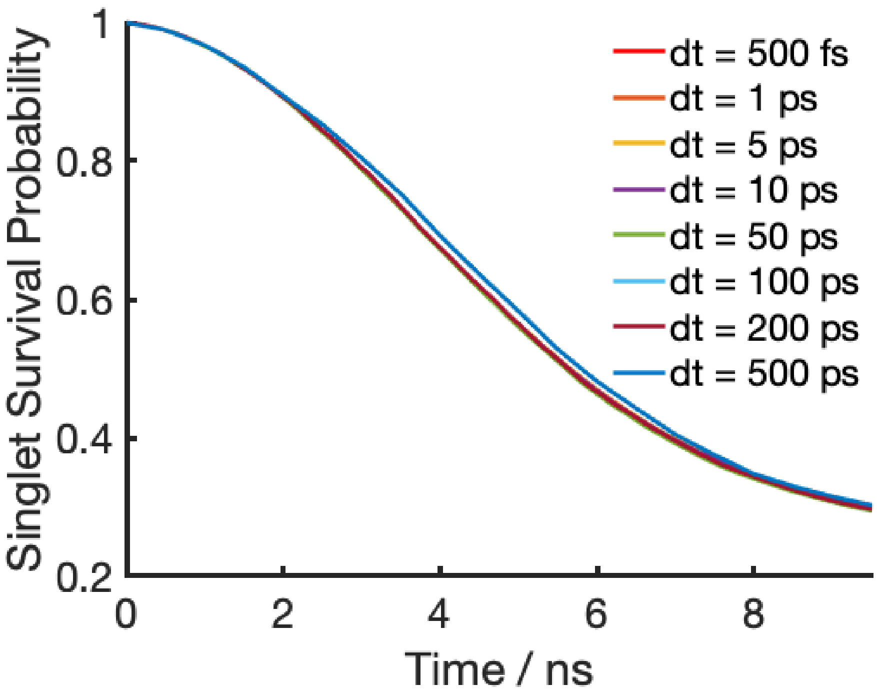

Figure S11: Singlet survival probability of a radical pair interaction with 4-nuclear spins vs. simulation time-steps. Parameters used in the calculation are described in the text of Section S6. The simulations were performed with different time-step dt to gauge what time-step is sufficient to model radical pair processes with the described MD data.

From this data, it can be concluded that $dt = 500$ ps most likely does not capture all the motions and $dt = 200$ ps was sufficient to model this system accurately. This is a surprising result since vibrations are known to have a shorter time scale for which the determined time step would be too long to capture these motions effectively. We believe that either the faster motions do not affect singlet survival probability, or the hyperfine interactions do not significantly change due to vibrations. Moreover, it would have been more accurate to compare longer simulation times because maybe some effects would only be visible for longer trajectories. As mentioned before, this is not computationally feasible due to the many DFT calculations required, which would require additional strategies. Also, this publication serves as an introduction to new *MolSpin* capabilities and methods and does not aim to fully analyse these results, which will be done in subsequent publications aimed at analysing short-time scale motion effects on spin dynamics of radical pairs in a protein environment. We will use $dt = 50$ ps for further simulations to ensure capturing all important motions of the spin interaction while still being computationally

feasible. Note, that the calculations of hyperfine interactions for the 0.953 μs MD trajectories and 50 ps step size led to the calculation of 19,060 quantum chemical calculations including a geometry optimization of the Hydrogen used to saturate the dangling bonds and the calculation of hyperfine coupling tensors using the Gaussian16 software [20].

S7 Average Hyperfines from MD data (953 ns)

This section includes average hypefine interactions and the average dipolar interaction for 953 ns trajectory MD data. The hyperfine interactions in this data set were computed with DFT for each 50 ps time frame. Average hyperfine interactions are displayed in Tables S6 and S7.

The average dipolar tensor:

$$D = \begin{pmatrix} 0.446 & 0.386 & 0.123 \\ 0.386 & -0.136 & 0.061 \\ 0.123 & 0.061 & -0.309 \end{pmatrix} \text{ mT} \tag{S30}$$

Table S6: Averaged hyperfine tensors for the $FAD^{\bullet-}$ radical of the European Robin cryptochrome 4a protein for a 953 μs MD-trajactory. $FAD^{\bullet-}$ structure and labels of each atom can be found in Fig. S12.

Atom	Hyperfine Tensor **A** / mT
N5	$\begin{pmatrix} 0.520 & -0.173 & 0.773 \\ -0.173 & 0.044 & -0.243 \\ 0.773 & -0.243 & 1.143 \end{pmatrix}$
N10	$\begin{pmatrix} 0.182 & -0.052 & 0.254 \\ -0.052 & 0.021 & -0.070 \\ 0.254 & -0.070 & 0.392 \end{pmatrix}$
H6	$\begin{pmatrix} -0.243 & -0.047 & -0.101 \\ -0.047 & -0.440 & 0.016 \\ -0.101 & 0.016 & -0.343 \end{pmatrix}$
$H8_1$	$\begin{pmatrix} 0.140 & -0.030 & -0.054 \\ -0.030 & 0.138 & 0.043 \\ -0.054 & 0.043 & 0.166 \end{pmatrix}$
$H8_2$	$\begin{pmatrix} 0.394 & -0.004 & -0.001 \\ -0.004 & 0.467 & 0.016 \\ -0.001 & 0.016 & 0.39743516 \end{pmatrix}$
$H8_3$	$\begin{pmatrix} 0.392 & -0.004 & -0.001 \\ -0.004 & 0.465 & 0.016 \\ -0.001 & 0.016 & 0.395 \end{pmatrix}$
$H\beta_1$	$\begin{pmatrix} 0.396 & -0.004 & -0.001 \\ -0.004 & 0.468 & 0.016 \\ -0.001 & 0.016 & 0.399 \end{pmatrix}$

Table S7: Averaged hyperfine tensors for $\text{TrpH}^{\bullet+}$ radical of the European Robin cryptochrome 4a protein for a 953 μs MD-trajectory. $\text{TrpH}^{\bullet+}$ structure and labels of each atom can be found in Fig. S12.

Atom	Hyperfine Tensor $\mathbf{A}$ / mT
N1	$\begin{pmatrix} 0.527 & 0.358 & 0.293 \\ 0.358 & 0.291 & 0.214 \\ 0.293 & 0.214 & 0.226 \end{pmatrix}$
H1	$\begin{pmatrix} -0.664 & 0.180 & -0.013 \\ 0.180 & -0.507 & -0.340 \\ -0.013 & -0.340 & -0.046 \end{pmatrix}$
H2	$\begin{pmatrix} -0.384 & -0.207 & 0.096 \\ -0.207 & -0.168 & -0.031 \\ 0.096 & -0.031 & -0.589 \end{pmatrix}$
H4	$\begin{pmatrix} -0.650 & 0.167 & -0.115 \\ 0.167 & -0.708 & -0.153 \\ -0.115 & -0.153 & -0.345 \end{pmatrix}$
H6	$\begin{pmatrix} -0.201 & -0.137 & -0.046 \\ -0.137 & -0.240 & 0.119 \\ -0.046 & 0.119 & -0.355 \end{pmatrix}$
H7	$\begin{pmatrix} -0.421 & 0.104 & -0.091 \\ 0.104 & -0.427 & -0.122 \\ -0.091 & -0.122 & -0.196 \end{pmatrix}$
Hβ_1	$\begin{pmatrix} 0.228 & -0.053 & -0.089 \\ -0.053 & 0.196 & 0.044 \\ -0.089 & 0.044 & 0.269 \end{pmatrix}$

Structures of both radicals with atoms labels are shown in Fig. S12.

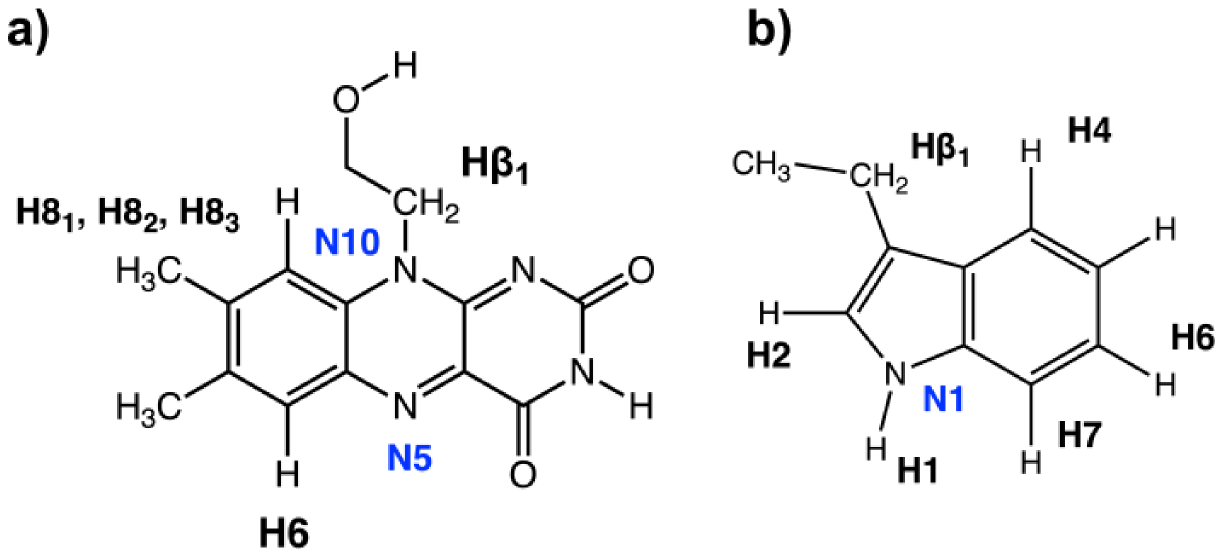

Figure S12: Molecular structures and labeled atoms of a) $FAD^{\bullet-}$ and b) $TrpH^{\bullet+}$ used for MD data (953 ns) modelling. Labels are defined according to the manuscript and Ref. [2] and a different to the 500 ns labeling by Ref. [19]

S8 *MolSpin* Features & Input Files

This section contains the input files we have used for various simulations presented in this article, that would enable another user new to *MolSpin* to try and test out the methods themselves.

S8.1 Format of .mst Files

The *MolSpin* trajectory (**mst**) files are used to integrate complex time-dependencies into a spin dynamics calculation. The files are constucted in a ways that either specific interactions or reactions rates can be adjusted with time-dependencies. The following example shows a .mst file for an N5 nucleus in its eigenframe extracted from the 550 ns MD simulation, when only modulating the A_{zz} component of the hyperfine coupling tensor:

```
time  mat.xx mat.xy mat.xz mat.yx mat.yy mat.yz mat.zx mat.zy mat.zz
0.00000000 -0.00009359 0.00000000 0.00000000 0.00000000 -0.00009359 0.00000000 0.00000000 0.00000000 0.00185977
0.05000000 -0.00009359 0.00000000 0.00000000 0.00000000 -0.00009359 0.00000000 0.00000000 0.00000000 0.00188710
0.10000000 -0.00009359 0.00000000 0.00000000 0.00000000 -0.00009359 0.00000000 0.00000000 0.00000000 0.00188667
0.15000000 -0.00009359 0.00000000 0.00000000 0.00000000 -0.00009359 0.00000000 0.00000000 0.00000000 0.00188617
0.20000000 -0.00009359 0.00000000 0.00000000 0.00000000 -0.00009359 0.00000000 0.00000000 0.00000000 0.00188876
0.25000000 -0.00009359 0.00000000 0.00000000 0.00000000 -0.00009359 0.00000000 0.00000000 0.00000000 0.00188732
.
.
.
```

The first column is the time which will be set in nanoseconds while the nine following rows describe the time-dependencies of the nine hyperfine tensor elements. As can be observed only the A_{zz} component is altered. *MolSpin* is recognizing the format of the .mst file through the first line. There are many other forms that can be used to include complex time-dependencies for field fluctuations, reaction rate fluctuations or fluctuations of principal components of certain coupling tensors. More details can be found in the manual.

Each .mst file can be attributed to a specific object via the *trajectory* keyword:

```
// -------------------------
// Hyperfine interactions
// -------------------------
Interaction FADHYP1
{
    IgnoreTensors = true;
    CommonPrefactor = false;
    type = hyperfine;
    group1 = E1;
    group2 = FADN5;
    tensor = trajectory("motions_azz005ns.mst");
    trajectory = "motions_azz_005ns.mst";
}
```

S8.2 12 Nuclear Spin System - Figure 1b

The template for running the 12 nuclear spin radical pair system of $\left[\mathrm{FAD}^{\bullet-}\ \mathrm{TrpH}^{\bullet+}\right]$ that was used to compare the accuracy of the stochastic method to the full dynamics method that utilizes diagonalization (described in Ref. [21]). This is an example template for a simulation with $MC = 1$ and this variable should be adjusted to other values if needed. The calculations done in the article had this number ranging from 1 to 50.

```
SpinSystem system1
{
    // ---------------------------------------------------------
    // Spins
    // ---------------------------------------------------------

    // Electrons
    Spin E1 {type = electron; spin = 1/2; tensor = isotropic(2.0023);}
    Spin E2 {type = electron; spin = 1/2; tensor = isotropic(2.0023);}

    // Nitrogens
    Spin FADN5 {type = nucleus; spin = 1; tensor = isotropic(1);}
    Spin FADN10{type = nucleus; spin = 1;tensor = isotropic(1);}

    Spin TRPN1 {type = nucleus; spin = 1;tensor = isotropic(1);}

    // Hydrogens
    Spin FADH6 {type = nucleus; spin = 1/2;tensor = isotropic(1);}
    Spin FADH81 {type = nucleus; spin = 1/2;tensor = isotropic(1);}
    Spin FADH82 {type = nucleus; spin = 1/2;tensor = isotropic(1);}
    Spin FADH83 {type = nucleus; spin = 1/2;tensor = isotropic(1);}
    Spin FADHbeta {type = nucleus; spin = 1/2;tensor = isotropic(1);}

    Spin TRPH1 {type = nucleus; spin = 1/2;tensor = isotropic(1);}
    Spin TRPH2 {type = nucleus; spin = 1/2;tensor = isotropic(1);}
    Spin TRPH4 {type = nucleus; spin = 1/2;tensor = isotropic(1);}
    Spin TRPH6 {type = nucleus; spin = 1/2;tensor = isotropic(1);}

    // -------------------------
    // Zeeman interactions
    // -------------------------

    Interaction Zeeman1 {prefactor= 1e-3; type = zeeman; field = "0.0 0.0 1.0"; spins = E1;}
    Interaction Zeeman2 {prefactor= 1e-3; type = zeeman; field = "0.0 0.0 1.0"; spins = E2;}

    // -------------------------
    // Hyperfine interactions
    // -------------------------

    Interaction FADHYP1 {prefactor = 1e-3; type = hyperfine; group1 = E1; group2 = FADN5; tensor = matrix("-0.0989, 0.0039,
      0.0; 0.0039, -0.0881, 0.0; 0.0, 0.0, 1.7569");}
    Interaction FADHYP2 {prefactor = 1e-3; type = hyperfine; group1 = E1; group2 = FADN10; tensor = matrix("-0.0190, -0.0048,
      0.0; -0.0048, -0.0196, 0.0; 0.0, 0.0, 0.6046");}
    Interaction FADHYP3 {prefactor = 1e-3; type = hyperfine; group1 = E1; group2 = FADH6; tensor = matrix("-0.2569, -0.1273,
      0.0; -0.1273, -0.4711, 0.0; 0.0, 0.0, -0.4336");}
    Interaction FADHYP4 {prefactor = 1e-3; type = hyperfine; group1 = E1; group2 = FADH81; tensor = matrix("0.4399, 0.0, 0.0;
      0.0, 0.4399, 0.0; 0.0, 0.0, 0.4399");}
    Interaction FADHYP5 {prefactor = 1e-3; type = hyperfine; group1 = E1; group2 = FADH82; tensor = matrix("0.4399, 0.0, 0.0;
      0.0, 0.4399, 0.0; 0.0, 0.0, 0.4399");}
    Interaction FADHYP6 {prefactor = 1e-3; type = hyperfine; group1 = E1; group2 = FADH83; tensor = matrix("0.4399, 0.0, 0.0;
      0.0, 0.4399, 0.0; 0.0, 0.0, 0.4399");}
    Interaction FADHYP7 {prefactor = 1e-3; type = hyperfine; group1 = E1; group2 = FADHbeta; tensor = matrix("0.4070, 0.0,
      0.0; 0.0, 0.4070, 0.0; 0.0, 0.0, 0.4070");}

    Interaction TRPHYP1 {prefactor = 1e-3; type = hyperfine; group1 = E2; group2 = TRPN1; tensor = matrix("-0.0336, 0.0924,
     -0.1354; 0.0924, 0.3303, -0.5318; -0.1354, -0.5318, 0.6680");}
    Interaction TRPHYP2 {prefactor = 1e-3; type = hyperfine; group1 = E2; group2 = TRPH1; tensor = matrix("-0.9920, -0.2091,
     -0.2003; -0.2091, -0.2631, 0.2803; -0.2003, 0.2803, -0.5398");}
    Interaction TRPHYP3 {prefactor = 1e-3; type = hyperfine; group1 = E2; group2 = TRPH2; tensor = matrix("-0.2843, 0.1757,
      0.1525; 0.1757, -0.2798, 0.0975; 0.1525, 0.0975, -0.2699");}
    Interaction TRPHYP4 {prefactor = 1e-3; type = hyperfine; group1 = E2; group2 = TRPH4; tensor = matrix("-0.5596, -0.1956,
     -0.1657; -0.1956, -0.4020, 0.0762; -0.1657, 0.0762, -0.5021");}
    Interaction TRPHYP5 {prefactor = 1e-3; type = hyperfine; group1 = E2; group2 = TRPH6; tensor = matrix("-0.0506, 0.0622,
      0.0889; 0.0622, -0.3100, -0.0297; 0.0889, -0.0297, 0.2642");}

    // -------------------------
    // Dipolar interactions
    // -------------------------

    Interaction Dipolar {prefactor = 2.0023e-3; IgnoreTensors = true; type = doublespin; group1 = E1; group2 = E2; tensor =
     matrix("-0.382276, 0.292979, -0.146796; 0.292979, -0.652196, 0.229243;-0.146796, 0.229243, -0.309528");}
     // We ignore the tensor variable from the Spin objects  and add 2.0023 to the prefactor to only account for one g-value
      for conversion from Tesla into rad/ns (MolSpin multiplies each interaction by a CommonPrefactor (Bohr Magneton/hbar) if
      not set to     false

    // ---------------------------------------------------------
    // Spin States
    // ---------------------------------------------------------

    State Singlet {spins(E1,E2) = |1/2,-1/2> - |-1/2,1/2>;}
    State T0 {spins(E1,E2) = |1/2,-1/2> + |-1/2,1/2>;}
    State Tp {spin(E2) = |1/2>; spin(E1) = |1/2>;} // |T+>
    State Tm { spin(E2) = |-1/2>; spin(E1) = |-1/2>;} // |T->
    State Identity {}

    // ---------------------------------------------------------
    // Transitions
    // ---------------------------------------------------------

    Transition Product1 {type = sink; source = Singlet; rate = 1e-3;} // in 1/ns
    Transition Product2 {type = sink; source = T0; rate = 1e-3;}
    Transition Product3 {type = sink; source = Tp; rate = 1e-3;}
    Transition Product4 {type = sink; source = Tm; rate = 1e-3;}

}
// ---------------------------------------------------------
Settings
{
    Settings general {steps = 51; notifications = details;}

```

```
    // -----------------------------------------------------------
    // Actions
    // -----------------------------------------------------------

    Action scan1 {type = rotatevector; vector = system1.Zeeman1.field; axis = "0 1 0"; value = 3.6;}
    Action scan2 {type = rotatevector; vector = system1.Zeeman2.field; axis = "0 1 0"; value = 3.6;}

    // -----------------------------------------------------------
    // Outputs objects
    // -----------------------------------------------------------

    Output orientation1 {type = vectorangle; vector = system1.Zeeman1.field; reference = "0 0 1";}
    Output orientation2 {type = vectorangle; vector = system1.Zeeman2.field; reference = "0 0 1";}
}
// -----------------------------------------------------------
Run
{
    Task Static12SpinSystem {type = "statichs-stoch-yields"; logfile = "staticyield_12spins.log"; datafile =
      "staticyield_12spins.dat"; transitionyields = true; initialstate = singlet; samplingmethod = "SUZ"; autoseed = false;
      seed = 2.0;  montecarlosamples = 1; epsilon = 0.001; timestep = 4; propagationmethod = "AutoExpm"; precision =
      "single"; yieldcorrections = true;}
}
```

S8.3 20 Nuclear Spin System - Figure 1c

This input template was used to calculate the spin dynamics of the 20 nuclear spin system of $\left[\mathrm{FAD}^{\bullet-}\ \mathrm{TrpH}^{\bullet+}\right]$, to highlight the computational capabilities of the method. The actual calculation was done by calculating each θ point separately in the cluster just for the reason that the whole computation could be finished faster than running it on one node for a long period of time.

```
SpinSystem system1
{
    // -----------------------------------------------------------
    // Spins
    // -----------------------------------------------------------

    // Electrons
    Spin E1 {type = electron; spin = 1/2; tensor = isotropic(2.0023);}
    Spin E2 {type = electron; spin = 1/2; tensor = isotropic(2.0023);}

    // Nitrogens
    Spin FADN5 {type = nucleus; spin = 1;  tensor = isotropic(1);}
    Spin FADN10 {type = nucleus; spin = 1;  tensor = isotropic(1);}

    Spin TRPN1 {type = nucleus; spin = 1;  tensor = isotropic(1);}

    // Hydrogens
    Spin FADH6 {type = nucleus; spin = 1/2;  tensor = isotropic(1);}
    Spin FADH81 {type = nucleus; spin = 1/2;  tensor = isotropic(1);}
    Spin FADH82 {type = nucleus; spin = 1/2;  tensor = isotropic(1);}
    Spin FADH83 {type = nucleus; spin = 1/2;  tensor = isotropic(1);}
    Spin FADHbeta1 {type = nucleus; spin = 1/2;  tensor = isotropic(1);}
    Spin FADHbeta2 {type = nucleus; spin = 1/2;  tensor = isotropic(1);}
    Spin FADH71 {type = nucleus; spin = 1/2;  tensor = isotropic(1);}
    Spin FADH72 {type = nucleus; spin = 1/2;  tensor = isotropic(1);}

    Spin TRPH1 {type = nucleus; spin = 1/2;  tensor = isotropic(1);}
    Spin TRPH2 {type = nucleus; spin = 1/2;  tensor = isotropic(1);}
    Spin TRPH4 {type = nucleus; spin = 1/2;  tensor = isotropic(1);}
    Spin TRPH6 {type = nucleus; spin = 1/2;  tensor = isotropic(1);}
    Spin TRPH7 {type = nucleus; spin = 1/2;  tensor = isotropic(1);}
    Spin TRPHbeta1 {type = nucleus; spin = 1/2;  tensor = isotropic(1);}
    Spin TRPHalpha {type = nucleus; spin = 1/2;  tensor = isotropic(1);}
    Spin TRPNasterisk {type = nucleus; spin = 1;  tensor = isotropic(1);}
    Spin TRPHbeta2 {type = nucleus; spin = 1/2;  tensor = isotropic(1);}

    // -------------------------
    // Zeeman interaction
    // -------------------------

    Interaction Zeeman1 {prefactor= 1e-3; type = zeeman; field = "0.0 0.0 1.0"; spins = E1;}

    Interaction Zeeman2 {prefactor= 1e-3; type = zeeman; field = "0.0 0.0 1.0"; spins = E2;}

    // -------------------------
    // Hyperfine interactions
    // -------------------------
    Interaction FADHYP1 {prefactor = 1e-3; type = hyperfine; group1 = E1; group2 = FADN5; tensor = matrix("-0.0989, 0.0039,
      0.0; 0.0039, -0.0881, 0.0; 0.0, 0.0, 1.7569");}
    Interaction FADHYP2 {prefactor = 1e-3; type = hyperfine; group1 = E1; group2 = FADN10; tensor = matrix("-0.0190, -0.0048,
      0.0; -0.0048, -0.0196, 0.0; 0.0, 0.0, 0.6046");}
    Interaction FADHYP3 {prefactor = 1e-3; type = hyperfine; group1 = E1; group2 = FADH6; tensor = matrix("-0.2569, -0.1273,
      0.0; -0.1273, -0.4711, 0.0; 0.0, 0.0, -0.4336");}
    Interaction FADHYP4 {prefactor = 1e-3; type = hyperfine; group1 = E1; group2 = FADH81; tensor = matrix("0.4399, 0.0, 0.0;
      0.0, 0.4399, 0.0; 0.0, 0.0, 0.4399");}
    Interaction FADHYP5 {prefactor = 1e-3; type = hyperfine; group1 = E1; group2 = FADH82; tensor = matrix("0.4399, 0.0, 0.0;
      0.0, 0.4399, 0.0; 0.0, 0.0, 0.4399");}
    Interaction FADHYP6 {prefactor = 1e-3; type = hyperfine; group1 = E1; group2 = FADH83; tensor = matrix("0.4399, 0.0, 0.0;
      0.0, 0.4399, 0.0; 0.0, 0.0, 0.4399");}
    Interaction FADHYP7 {prefactor = 1e-3; type = hyperfine; group1 = E1; group2 = FADHbeta1; tensor = matrix("0.4070, 0.0,
      0.0; 0.0, 0.4070, 0.0; 0.0, 0.0, 0.4070");}
    Interaction FADHYP8 {prefactor = 1e-3; type = hyperfine; group1 = E1; group2 = FADHbeta2; tensor = matrix("0.4070, 0.0,
      0.0; 0.0, 0.4070, 0.0; 0.0, 0.0, 0.4070");}
    Interaction FADHYP9 {prefactor = 1e-3; type = hyperfine; group1 = E1; group2 = FADH71; tensor = matrix("-0.1416, 0.0,
      0.0; 0.0, -0.1416, 0.0; 0.0, 0.0, -0.1416");}
    Interaction FADHYP10 {prefactor = 1e-3; type = hyperfine; group1 = E1; group2 = FADH72; tensor = matrix("-0.1416, 0.0,
      0.0; 0.0, -0.1416, 0.0; 0.0, 0.0, -0.1416");}
```

```

    Interaction TRPHYP1 {prefactor = 1e-3; type = hyperfine; group1 = E2; group2 = TRPN1; tensor = matrix("-0.0336, 0.0924,
      -0.1354; 0.0924, 0.3303, -0.5318; -0.1354, -0.5318, 0.6680");}
    Interaction TRPHYP2 {prefactor = 1e-3; type = hyperfine; group1 = E2; group2 = TRPH1; tensor = matrix("-0.9920, -0.2091,
      -0.2003; -0.2091, -0.2631, 0.2803; -0.2003, 0.2803, -0.5398");}
    Interaction TRPHYP3 {prefactor = 1e-3; type = hyperfine; group1 = E2; group2 = TRPH2; tensor = matrix("-0.2843, 0.1757,
      0.1525; 0.1757, -0.2798, 0.0975; 0.1525, 0.0975, -0.2699");}
    Interaction TRPHYP4 {prefactor = 1e-3; type = hyperfine; group1 = E2; group2 = TRPH4; tensor = matrix("-0.5596, -0.1956,
      -0.1657; -0.1956, -0.4020, 0.0762; -0.1657, 0.0762, -0.5021");}
    Interaction TRPHYP5 {prefactor = 1e-3; type = hyperfine; group1 = E2; group2 = TRPH6; tensor = matrix("-0.0506, 0.0622,
      0.0889; 0.0622, -0.3100, -0.0297; 0.0889, -0.0297, 0.2642");}
    Interaction TRPHYP6 {prefactor = 1e-3; type = hyperfine; group1 = E2; group2 = TRPH7; tensor = matrix("-0.4355, -0.1541,
      -0.1239; -0.1541, -0.2777, 0.0864; -0.1239, 0.0864, -0.3770");}
    Interaction TRPHYP7 {prefactor = 1e-3; type = hyperfine; group1 = E2; group2 = TRPHbeta1; tensor = matrix("1.5808,
      -0.0453, -0.0506; -0.0453, 1.5575, 0.0988; -0.0506, 0.0988, 1.6752");}
    Interaction TRPHYP8 {prefactor = 1e-3; type = hyperfine; group1 = E2; group2 = TRPHalpha; tensor = matrix("-0.0601,
      0.0037, 0.0331; 0.0037, -0.0251, 0.0111; 0.0331, 0.0111, -0.1940");}
    Interaction TRPHYP9 {prefactor = 1e-3; type = hyperfine; group1 = E2; group2 = TRPNasterisk; tensor = matrix("0.1295,
      -0.0134, 0.0075; -0.0134, 0.1729, -0.0249; 0.0075, -0.0249, 0.1371");}
    Interaction TRPHYP10 {prefactor = 1e-3; type = hyperfine; group1 = E2; group2 = TRPHbeta2; tensor = matrix("0.1634,
      -0.0230, -0.0064; -0.0230, -0.0082, 0.0158; -0.0064, 0.0158, -0.0182");}

    // -------------------------
    // Dipolar interactions
    // -------------------------

    Interaction Dipolar {prefactor = 2.0023e-3; IgnoreTensors = true; type = doublespin; group1 = E1; group2 = E2; tensor =
      matrix("-0.382276, 0.292979, -0.146796; 0.292979, -0.652196, 0.229243;-0.146796, 0.229243, -0.309528");}
    // We ignore the tensor variable from the Spin objects  and add 2.0023 to the prefactor to only account for one g-value
      for conversion from Tesla into rad/ns (MolSpin multiplies each interaction by a CommonPrefactor (Bohr Magneton/hbar) if
      not set to    false

    // -------------------------
    // Spin States
    // ---------------------------------------------------------
    State Singlet {spins(E1,E2) = |1/2,-1/2> - |-1/2,1/2>;}
    State T0 {spins(E1,E2) = |1/2,-1/2> + |-1/2,1/2>;}
    State Tp {spin(E2) = |1/2>; spin(E1) = |1/2>;} // |T+>
    State Tm {spin(E2) = |-1/2>; spin(E1) = |-1/2>;} // |T->
    State Identity {}

    // ---------------------------------------------------------
    // Transitions
    // ---------------------------------------------------------

    Transition Product1 {type = sink; source = Singlet; rate = 1e-3;} // in 1/ns
    Transition Product2 {type = sink; source = T0; rate = 1e-3;}
    Transition Product3 {type = sink; source = Tp; rate = 1e-3;}
    Transition Product4 {type = sink; source = Tm; rate = 1e-3;}
}
// ---------------------------------------------------------
Settings
{
    Settings general {steps = 51; notifications = details;}

    // ---------------------------------------------------------
    // Actions
    // ---------------------------------------------------------

    Action scan1 {type = rotatevector; vector = system1.Zeeman1.field; axis = "0 1 0"; value = 3.6;}
    Action scan2 {type = rotatevector; vector = system1.Zeeman2.field; axis = "0 1 0"; value = 3.6;}

    // ---------------------------------------------------------
    // Outputs objects
    // ---------------------------------------------------------

    Output orientation1 {type = vectorangle; vector = system1.Zeeman1.field; reference = "0 0 1";}
    Output orientation2 {type = vectorangle; vector = system1.Zeeman2.field; reference = "0 0 1";}
}
// ---------------------------------------------------------
Run
{
    Task Stati20SpinSystem{type = "statichs-stoch-yields"; logfile = "staticyield_20spins.log"; datafile =
      "staticyield_20spins.dat"; transitionyields = true; initialstate = singlet; samplingmethod = "SUZ"; autoseed = false;
      seed = 2.0; montecarlosamples = 1; epsilon = 0.5; timestep = 4; propagationmethod = "AutoExpm"; precision = "single";
      yieldcorrections = true;}
}
```

S8.4 Molecular Wires - Figure 2b

This is an input file that was used to replicate the relative yield versus magnetic field data for the molecular wire $PTZ^{\bullet+} - Ph_4 - PDI^{\bullet-}$from Ref. [22]. To replicate the whole data set the variable m in the Zeeman interaction objects should be set to the magnetic field of interest (in mT). The magnetic field range used to replicate the graph was $0 - 12\ mT$.

```
SpinSystem system1
{
    // ---------------------------------------------------------
    // Spins
    // ---------------------------------------------------------

    // Electrons
    Spin E1 { type = electron; spin = 1/2; tensor = isotropic(2.0023); }
    Spin E2 { type = electron; spin = 1/2; tensor = isotropic(2.0023); }

    //Nitrogens
    Spin Rad1N1 {type=nucleus; spin=1; tensor = isotropic(1);}
    Spin Rad2N1 {type=nucleus; spin=1; tensor = isotropic(1);}
    Spin Rad2N2 {type=nucleus; spin=1; tensor = isotropic(1);}

    //Hydrogens
    Spin Rad1H1 {type=nucleus; spin=1/2; tensor = isotropic(1);}
    Spin Rad1H2 {type=nucleus; spin=1/2; tensor = isotropic(1);}
    Spin Rad1H3 {type=nucleus; spin=1/2; tensor = isotropic(1);}
    Spin Rad1H4 {type=nucleus; spin=1/2; tensor = isotropic(1);}
    Spin Rad1H5 {type=nucleus; spin=1/2; tensor = isotropic(1);}
    Spin Rad1H6 {type=nucleus; spin=1/2; tensor = isotropic(1);}
    Spin Rad1H7 {type=nucleus; spin=1/2; tensor = isotropic(1);}
    Spin Rad1H8 {type=nucleus; spin=1/2; tensor = isotropic(1);}
    Spin Rad2H1 {type=nucleus; spin=1/2; tensor = isotropic(1);}
    Spin Rad2H2 {type=nucleus; spin=1/2; tensor = isotropic(1);}
    Spin Rad2H3 {type=nucleus; spin=1/2; tensor = isotropic(1);}
    Spin Rad2H4 {type=nucleus; spin=1/2; tensor = isotropic(1);}
    Spin Rad2H5 {type=nucleus; spin=1/2; tensor = isotropic(1);}
    Spin Rad2H6 {type=nucleus; spin=1/2; tensor = isotropic(1);}

    // -------------------------
    // Zeeman interaction
    // -------------------------

    Interaction Zeeman1 {prefactor=1e-3; type=zeeman; field="0.0 0.0 m"; spins=E1;}
    Interaction Zeeman2 {prefactor=1e-3; type=zeeman; field="0.0 0.0 m"; spins=E2;}

    // -------------------------
    // Hyperfine interactions
    // -------------------------

    Interaction Rad1HYP1 {prefactor=1e-3; type=hyperfine; group1=E1; group2=Rad1H1; tensor=matrix("-0.113, 0.0, 0.0; 0.0, -0.113, 0.0; 0.0, 0.0, -0.113");}
    Interaction Rad1HYP2 {prefactor=1e-3; type=hyperfine; group1=E1; group2=Rad1H2; tensor=matrix("-0.113, 0.0, 0.0; 0.0, -0.113, 0.0; 0.0, 0.0, -0.113");}
    Interaction Rad1HYP3 {prefactor=1e-3; type=hyperfine; group1=E1; group2=Rad1H3; tensor=matrix("-0.05, 0.0, 0.0; 0.0, -0.05, 0.0; 0.0, 0.0, -0.05");}
    Interaction Rad1HYP4 {prefactor=1e-3; type=hyperfine; group1=E1; group2=Rad1H4; tensor=matrix("-0.05, 0.0, 0.0; 0.0, -0.05, 0.0; 0.0, 0.0, -0.05");}
    Interaction Rad1HYP5 {prefactor=1e-3; type=hyperfine; group1=E1; group2=Rad1H5; tensor=matrix("-0.249, 0.0, 0.0; 0.0, -0.249, 0.0; 0.0, 0.0, -0.249");}
    Interaction Rad1HYP6 {prefactor=1e-3; type=hyperfine; group1=E1; group2=Rad1H6; tensor=matrix("-0.249, 0.0, 0.0; 0.0, -0.249, 0.0; 0.0, 0.0, -0.249");}
    Interaction Rad1HYP7 {prefactor=1e-3; type=hyperfine; group1=E1; group2=Rad1H7; tensor=matrix("0.050, 0.0, 0.0; 0.0, 0.050, 0.0; 0.0, 0.0, 0.050");}
    Interaction Rad1HYP8 {prefactor=1e-3; type=hyperfine; group1=E1; group2=Rad1H8; tensor=matrix("0.050, 0.0, 0.0; 0.0, 0.050, 0.0; 0.0, 0.0, 0.050");}
    Interaction Rad1HYP9 {prefactor=1e-3; type=hyperfine; group1=E1; group2=Rad1N1; tensor=matrix("0.634, 0.0, 0.0; 0.0, 0.634, 0.0; 0.0, 0.0, 0.634");}

    Interaction Rad2Hyp1 {prefactor=1e-3; type=hyperfine; group1=E2; group2=Rad2H1; tensor=matrix("0.0785, 0.0, 0.0; 0.0, 0.0785, 0.0; 0.0, 0.0, 0.0785");}
    Interaction Rad2Hyp2 {prefactor=1e-3; type=hyperfine; group1=E2; group2=Rad2H2; tensor=matrix("0.0785, 0.0, 0.0; 0.0, 0.0785, 0.0; 0.0, 0.0, 0.0785");}
    Interaction Rad2Hyp3 {prefactor=1e-3; type=hyperfine; group1=E2; group2=Rad2H3; tensor=matrix("-0.1720, 0.0, 0.0; 0.0, -0.1720, 0.0; 0.0, 0.0, -0.1720");}
    Interaction Rad2Hyp4 {prefactor=1e-3; type=hyperfine; group1=E2; group2=Rad2H4; tensor=matrix("-0.1720, 0.0, 0.0; 0.0, -0.1720, 0.0; 0.0, 0.0, -0.1720");}
    Interaction Rad2Hyp5 {prefactor=1e-3; type=hyperfine; group1=E2; group2=Rad2H5; tensor=matrix("0.0575, 0.0, 0.0; 0.0, 0.0575, 0.0; 0.0, 0.0, 0.0575");}
    Interaction Rad2Hyp6 {prefactor=1e-3; type=hyperfine; group1=E2; group2=Rad2H6; tensor=matrix("0.0575, 0.0, 0.0; 0.0, 0.0575, 0.0; 0.0, 0.0, 0.0575");}
    Interaction Rad2Hyp7 {prefactor=1e-3; type=hyperfine; group1=E2; group2=Rad2N1; tensor=matrix("-0.0621, 0.0, 0.0; 0.0, -0.0621, 0.0; 0.0, 0.0, -0.0621");}
    Interaction Rad2Hyp8 {prefactor=1e-3; type=hyperfine; group1=E2; group2=Rad2N2; tensor=matrix("-0.0621, 0.0, 0.0; 0.0, -0.0621, 0.0; 0.0, 0.0, -0.0621");}

    // -------------------------
    // Dipolar interactions
    // -------------------------

     Interaction Dipolar {prefactor=2.0023e-3; IgnoreTensors=true; type=doublespin; group1=E1; group2=E2; tensor=matrix("6.4, 0.0, 0.0; 0.0, 6.4, 0.0; 0.0, 0.0, 6.4");}
    // We ignore the tensor variable from the Spin objects  and add 2.0023 to the prefactor to only account for one g-value for conversion from Tesla into rad/ns (MolSpin multiplies each interaction by a CommonPrefactor (Bohr Magneton/hbar) if not set to    false

    // -------------------------
    // Spin States
    // ---------------------------------------------------------

    State Singlet {spins(E1,E2)=|1/2,-1/2>-|-1/2,1/2>;}
    State T0 {spins(E1,E2)=|1/2,-1/2>+|-1/2,1/2>;}
    State Tp {spin(E2)=|1/2>; spin(E1)=|1/2>;}
    State Tm {spin(E2)=-|1/2>; spin(E1)=-|1/2>;}
    State Identity {}

    // ---------------------------------------------------------
    // Transitions
    // ---------------------------------------------------------

    Transition Product1 {type=sink; source=Singlet; rate=2.45e-3;} // in 1/ns
    Transition Product2 {type=sink; source=T0; rate=350.0e-3;}
    Transition Product3 {type=sink; source=Tp; rate=350.0e-3;}
```

```
    Transition Product4 {type=sink; source=Tm; rate=350.0e-3;}

}
// -------------------------------------------------------------
Settings
{
    Settings general {steps = 1; notifications = details;}
}
Run
{
    Task StaticMolecularWires {type = "statichs-stoch-yields"; logfile = "molecularwires.log"; datafile =
      "molecularwires.dat"; transitionyields = true; initialstate = singlet; samplingmethod = "SUZ"; autoseed = false; seed =
      2.0; montecarlosamples = 1; totaltime = 55; timestep = 0.1; propagationmethod = "AutoExpm";  precision = "single";
      yieldcorrections = false;}
}
```

S8.5 Quantum Needle - Figure 3b

This is the input file for the singlet yield versus inclination angle graph for the $\left[\mathrm{FAD}^{\bullet-}\ \mathrm{TrpH}^{\bullet+}\right]$ 14 nuclear spin radical pair system. This is the same system as the Quantum Needle example from Ref. [2], yet with EED and exchange interactions included, taken from Ref. [3].

```
SpinSystem system1
{
    // -------------------------------------------------------
    // Spins
    // --------------------------------------------------------

    // Electrons
    Spin E1 {type = electron; spin = 1/2; tensor = isotropic(2.0023);}
    Spin E2 {type = electron; spin = 1/2; tensor = isotropic(2.0023);}

    //Nitrogens
    Spin FADN5 {type = nucleus; spin = 1; tensor = isotropic(1);}
    Spin FADN10 {type = nucleus; spin = 1; tensor = isotropic(1);}

    Spin TRPN1 {type = nucleus; spin = 1; tensor = isotropic(1);}

    // Hydrogens
    Spin FADH6 {type = nucleus; spin = 1/2; tensor = isotropic(1);}
    Spin FADH81 {type = nucleus; spin = 1/2; tensor = isotropic(1);}
    Spin FADH82 {type = nucleus; spin = 1/2; tensor = isotropic(1);}
    Spin FADH83 {type = nucleus; spin = 1/2; tensor = isotropic(1);}
    Spin FADHbeta {type = nucleus; spin = 1/2; tensor = isotropic(1);}

    Spin TRPH1 {type = nucleus; spin = 1/2; tensor = isotropic(1);}
    Spin TRPH2 {type = nucleus; spin = 1/2; tensor = isotropic(1);}
    Spin TRPH4 {type = nucleus; spin = 1/2; tensor = isotropic(1);}
    Spin TRPH6 {type = nucleus; spin = 1/2; tensor = isotropic(1);}
    Spin TRPH7 {type = nucleus; spin = 1/2; tensor = isotropic(1);}
    Spin TRPHbeta1 {type = nucleus; spin = 1/2; tensor = isotropic(1);}

    // ------------------------
    // Zeeman interaction
    // ------------------------

    Interaction Zeeman1 {prefactor= 1e-3; type = zeeman; field = "0.0 0.0 0.050"; spins = E1;}
    Interaction Zeeman2 {prefactor= 1e-3; type = zeeman; field = "0.0 0.0 0.050"; spins = E2;}

    // ------------------------
    // Hyperfine interactions
    // ------------------------

    Interaction FADHYP1 {prefactor = 1e-3; type = hyperfine; group1 = E1; group2 = FADN5; tensor = matrix("-0.0989, 0.0039,
      0.0; 0.0039, -0.0881, 0.0; 0.0, 0.0, 1.7569");}
    Interaction FADHYP2 {prefactor = 1e-3; type = hyperfine; group1 = E1; group2 = FADN10; tensor = matrix("-0.0190, -0.0048,
      0.0; -0.0048, -0.0196, 0.0; 0.0, 0.0, 0.6046");}
    Interaction FADHYP3 {prefactor = 1e-3; type = hyperfine; group1 = E1; group2 = FADH6; tensor = matrix("-0.2569, -0.1273,
      0.0; -0.1273, -0.4711, 0.0; 0.0, 0.0, -0.4336");}
    Interaction FADHYP4 {prefactor = 1e-3; type = hyperfine; group1 = E1; group2 = FADH81; tensor = matrix("0.4399, 0.0, 0.0;
      0.0, 0.4399, 0.0; 0.0, 0.0, 0.4399");}
    Interaction FADHYP5 {prefactor = 1e-3; type = hyperfine; group1 = E1; group2 = FADH82; tensor = matrix("0.4399, 0.0, 0.0;
      0.0, 0.4399, 0.0; 0.0, 0.0, 0.4399");}
    Interaction FADHYP6 {prefactor = 1e-3; type = hyperfine; group1 = E1; group2 = FADH83; tensor = matrix("0.4399, 0.0, 0.0;
      0.0, 0.4399, 0.0; 0.0, 0.0, 0.4399");}
    Interaction FADHYP7 {prefactor = 1e-3; type = hyperfine; group1 = E1; group2 = FADHbeta; tensor = matrix("0.4070, 0.0,
      0.0; 0.0, 0.4070, 0.0; 0.0, 0.0, 0.4070");}

    Interaction TRPHYP1 {prefactor = 1e-3; type = hyperfine; group1 = E2; group2 = TRPN1; tensor = matrix("-0.0336, 0.0924,
      -0.1354; 0.0924, 0.3303, -0.5318; -0.1354, -0.5318, 0.6680");}
    Interaction TRPHYP2 {prefactor = 1e-3; type = hyperfine; group1 = E2; group2 = TRPH1; tensor = matrix("-0.9920, -0.2091,
      -0.2003; -0.2091, -0.2631, 0.2803; -0.2003, 0.2803, -0.5398");}
    Interaction TRPHYP3 {prefactor = 1e-3; type = hyperfine; group1 = E2; group2 = TRPH2; tensor = matrix("-0.2843, 0.1757,
      0.1525; 0.1757, -0.2798, 0.0975; 0.1525, 0.0975, -0.2699");}
    Interaction TRPHYP4 {prefactor = 1e-3; type = hyperfine; group1 = E2; group2 = TRPH4; tensor = matrix("-0.5596, -0.1956,
      -0.1657; -0.1956, -0.4020, 0.0762; -0.1657, 0.0762, -0.5021");}
    Interaction TRPHYP5 {prefactor = 1e-3; type = hyperfine; group1 = E2; group2 = TRPH6; tensor = matrix("-0.0506, 0.0622,
      0.0889; 0.0622, -0.3100, -0.0297; 0.0889, -0.0297, 0.2642");}
    Interaction TRPHYP6 {prefactor = 1e-3; type = hyperfine; group1 = E2; group2 = TRPH7; tensor = matrix("-0.4355, -0.1541,
      -0.1239; -0.1541, -0.2777, 0.0864; -0.1239, 0.0864, -0.3770");}
    Interaction TRPHYP7 {prefactor = 1e-3; type = hyperfine; group1 = E2; group2 = TRPHbeta1; tensor = matrix("1.5808,
      -0.0453, -0.0506; -0.0453, 1.5575, 0.0988; -0.0506, 0.0988, 1.6752");}

    // ------------------------
    // Dipolar interactions
    // ------------------------

    Interaction Dipolar {prefactor = 2.0023e-3; IgnoreTensors = true; type = doublespin; group1 = E1;  group2 = E2; tensor =
      matrix("-0.382276, 0.292979, -0.14679; 0.292979, -0.652196, 0.229243;-0.146796, 0.229243, -0.309528");}
    // We ignore the tensor variable from the Spin objects  and add 2.0023 to the prefactor to only account for one g-value
      for conversion from Tesla into rad/ns (MolSpin multiplies each interaction by a CommonPrefactor (Bohr Magneton/hbar) if
      not set to    false
    // ------------------------
    // Spin States
    // --------------------------------------------------------

    State Singlet {spins(E1,E2) = |1/2,-1/2> - |-1/2,1/2>;}
    State T0 {spins(E1,E2) = |1/2,-1/2> + |-1/2,1/2>;}
    State Tp {spin(E2) = |1/2>; spin(E1) = |1/2>;}
    State Tm {spin(E2) = |-1/2>; spin(E1) = |-1/2>;}
    State Identity { }

    // --------------------------------------------------------
    // Transitions
    // --------------------------------------------------------

    Transition Product1 {type = sink; source = Singlet; rate = 1e-3;} // in 1/ns
    Transition Product2 {type = sink; source = T0; rate = 1e-3;}
    Transition Product3 {type = sink; source = Tp; rate = 1e-3;}
    Transition Product4 {type = sink; source = Tm; rate = 1e-3;}
}
// ------------------------------------------------------------
Settings
{
    Settings general {steps = 51; notifications = details;}

    // --------------------------------------------------------
```

```
    // Actions
    // -----------------------------------------------------------

    Action scan1 {type = rotatevector; vector = system1.Zeeman1.field; axis = "0 1 0"; value = 3.6;}
    Action scan2 {type = rotatevector; vector = system1.Zeeman2.field; axis = "0 1 0"; value = 3.6;}

    // -----------------------------------------------------------
    // Outputs objects
    // -----------------------------------------------------------

    Output orientation1 {type = vectorangle; vector = system1.Zeeman1.field; reference = "0 0 1";}
    Output orientation2 {type = vectorangle; vector = system1.Zeeman2.field; reference = "0 0 1";}

}
// -----------------------------------------------------------------
Run
{
    Task Static14SpinsYield {type = "statichs-stoch-yields"; logfile = "test_staticyields_14spins.log"; datafile =
      "test_staticyields_14spins.dat"; transitionyields = true; initialstate = singlet; samplingmethod = "SUZ"; autoseed =
      false; seed = 2.0; montecarlosamples = 1; epsilon = 0.001; timestep = 4; propagationmethod = "AutoExpm"; precision =
      "single"; yieldcorrections = true;}
}
```

S8.6 Driven Recombination Dynamics - Figure 4b

This is the input file used to replicate results from Ref. [4]. Depending on the simulation case, the trajectories of the exchange interaction and recombination kinetics changed. The equations to form the trajectories were discussed in the article.

```
SpinSystem system1
{
    // ---------------------------------------------------------
    // Spins
    // ---------------------------------------------------------

    // Electrons
    Spin E1 {type=electron; spin=1/2; tensor=isotropic(2.0023);}
    Spin E2 {type=electron; spin=1/2; tensor=isotropic(2.0023);}
    // Nitrogens
    Spin FADN5 {type=nucleus; spin=1; tensor=isotropic(1);}

    // -------------------------
    // Zeeman interaction
    // -------------------------

    Interaction Zeeman1 {prefactor=1e-3; type=zeeman; field="0.0 0.0 0.05"; spins=E1;}
    Interaction Zeeman2 {prefactor=1e-3; type=zeeman; field="0.0 0.0 0.05"; spins=E2;}

    // -------------------------
    // Hyperfine interactions
    // -------------------------

    Interaction FADHYP1 {prefactor=1e-3; type=hyperfine; group1=E1; group2=FADN5; tensor=matrix("-0.092774, 0.0, 0.0; 0.0,
      0.092774, 0.0;  0.0, 0.0, 1.75558");}

    // -------------------------
    // Exchange interaction
    // -------------------------

    Interaction Exchange {prefactor=2.0023; IgnoreTensors=true; type=doublespin; group1=E1; group2=E2;
      tensor=trajectory("DipolarMu.mst"); trajectory="DipolarMu.mst";} // .mst file in T
    // We ignore the tensor variable from the Spin objects  and add 2.0023 to the prefactor to only account for one g-value
      for conversion from Tesla into rad/ns (MolSpin multiplies each interaction by a CommonPrefactor (Bohr Magneton/hbar) if
      not set to    false

    // -------------------------
    // Spin States
    // --------------------------------------------------------

    State Singlet {spins(E1,E2)=|1/2,-1/2>-|-1/2,1/2>;}
    State Identity {}

    // ---------------------------------------------------------
    // Transitions
    // ---------------------------------------------------------

    Transition Product1 {type=sink; source=Singlet; rate=trajectory("rate.mst"); trajectory="rate.mst";} // in 1/ns
    Transition Product2 {type=sink; source=Identity; rate=1.0e-3;}

}
// ---------------------------------------------------------
Settings
{
    Settings general {steps = 1; notifications = details;}
}
Run
{
    Task DynamicDrivenModelYield {type = "dynamichs-direct-yields"; logfile = "Dynamic1Spin.log"; datafile =
      "Dynamic1Spin.dat"; transitionyields = true; initialstate = Singlet; timestep = 1.0; totaltime = 12500;
      propagationmethod = "AutoExpm"; precision = "single"; yieldcorrections = false;}
}
```

S8.7 1 Nuclear Spin Relaxation (550 ns) - Figure S6

This script was used to compute the relaxation induced by fluctuating N5 hyperfine interaction with SSE method which was compared to the results available from BRW formalism (550 ns).

```
SpinSystem system1
{
// -----------------------------------------------------------
// Spins
// -----------------------------------------------------------

    // Electrons
    Spin E1 {type= electron; spin= 1/2; tensor= isotropic(2.0023)};
    Spin E2 {type= electron; spin= 1/2; tensor= isotropic(2.0023)};
    // Nitrogens
    Spin FADN5 {type= nucleus; spin= 1;; tensor= isotropic(1)};

    // -------------------------
    // Zeeman interaction
    // -------------------------

    Interaction Zeeman1 {prefactor=1e-3; type=zeeman; field="0.0 0.0 0.05"; spins=E1};
    Interaction Zeeman2 {prefactor=1e-3; type=zeeman; field="0.0 0.0 0.05"; spins=E2};

    // -------------------------
    // Hyperfine interactions
    // -------------------------

    Interaction FADHYP1 {type=hyperfine; group1=E1; group2=FADN5; tensor=trajectory("motions_N5.mst");
      trajectory="motions_N5.mst"}; // .mst file in T

    // -------------------------
    // Spin States
    // -----------------------------------------------------------

    State Singlet {spins(E1,E2) = |1/2,-1/2> - |-1/2,1/2>;}
    State T0 {spins(E1,E2) = |1/2,-1/2> + |-1/2,1/2>;}
    State Tp {spin(E2) = |1/2>; spin(E1) = |1/2>;}
    State Tm {spin(E2) = |-1/2>; spin(E1) = |-1/2>;}
    State Identity {}

    // -----------------------------------------------------------
    // Transitions
    // -----------------------------------------------------------

    Transition Product1 {type=sink; source=Singlet; rate=1.0e-3}; // in 1/ns
    Transition Product2 {type=sink; source=T0; rate=1.0e-3};
    Transition Product3 {type=sink; source=Tp; rate=1.0e-3};
    Transition Product4 {type=sink; source=Tm; rate=1.0e-3};

}
// -----------------------------------------------------------------
Settings
{
    Settings general {steps = 51; notifications = details;}

    // -----------------------------------------------------------
    // Actions
    // -----------------------------------------------------------

    Action scan1 {type = rotatevector; vector = system1.Zeeman1.field; axis = "0 1 0"; value = 3.6;}
    Action scan2 {type = rotatevector; vector = system1.Zeeman2.field; axis = "0 1 0"; value = 3.6;}

    // -----------------------------------------------------------
    // Outputs objects
    // -----------------------------------------------------------

    Output orientation1 {type = vectorangle; vector = system1.Zeeman1.field; reference = "0 0 1";}
    Output orientation2 {type = vectorangle; vector = system1.Zeeman2.field; reference = "0 0 1";}

}
Run
{
    Task DynamicDirectYield {type = "dynamichs-direct-yields"; logfile = "Dynamic4Spins.log"; datafile = "Dynamic4Spins.dat";
      transitionyields = true; initialstate = Singlet; totaltime = 550.0; timestep = 0.5; propagationmethod = "AutoExpm";
      precision = "single"; yieldcorrections = true;}
}
```

To acquire results for a static system, this script was used:

```
SpinSystem system1
{
// -----------------------------------------------------------
// Spins
// -----------------------------------------------------------

    // Electrons
    Spin E1 {type= electron; spin= 1/2; tensor= isotropic(2.0023)};
    Spin E2 {type= electron; spin= 1/2; tensor= isotropic(2.0023)};
    // Nitrogens
    Spin FADN5 {type= nucleus; spin= 1;tensor= isotropic(1)};

    // -------------------------
    // Zeeman interaction
    // -------------------------

    Interaction Zeeman1 {prefactor=1e-3; type=zeeman; field="0.0 0.0 0.05"; spins=E1};
    Interaction Zeeman2 {prefactor=1e-3; type=zeeman; field="0.0 0.0 0.05"; spins=E2};

    // -------------------------
    // Hyperfine interactions
    // -------------------------

    Interaction FADHYP1 {prefactor=1e-3; type=hyperfine; group1=E1; group2=FADN5; tensor = matrix("-0.09359, 0.00000,
      0.00000; 0.00000, -0.09359, 0.00000; 0.00000, 0.00000, 1.88074");};

```

```
    // ------------------------
    // Spin States
    // -------------------------------------------------------

    State Singlet {spins(E1,E2) = |1/2,-1/2> - |-1/2,1/2>;}
    State T0 {spins(E1,E2) = |1/2,-1/2> + |-1/2,1/2>;}
    State Tp {spin(E2) = |1/2>; spin(E1) = |1/2>;}
    State Tm {spin(E2) = |-1/2>; spin(E1) = |-1/2>;}
    State Identity {}

    // -------------------------------------------------------
    // Transitions
    // -------------------------------------------------------

    Transition Product1 {type=sink; source=Singlet; rate=1.0e-3}; // in 1/ns
    Transition Product2 {type=sink; source=T0; rate=1.0e-3};
    Transition Product3 {type=sink; source=Tp; rate=1.0e-3};
    Transition Product4 {type=sink; source=Tm; rate=1.0e-3};

}
// -----------------------------------------------------------
Settings
{
    Settings general {steps = 51; notifications = details;}

    // -------------------------------------------------------
    // Actions
    // -------------------------------------------------------

    Action scan1 {type = rotatevector; vector = system1.Zeeman1.field; axis = "0 1 0"; value = 3.6;}
    Action scan2 {type = rotatevector; vector = system1.Zeeman2.field; axis = "0 1 0"; value = 3.6;}

    // -------------------------------------------------------
    // Outputs objects
    // -------------------------------------------------------

    Output orientation1 {type = vectorangle; vector = system1.Zeeman1.field; reference = "0 0 1";}
    Output orientation2 {type = vectorangle; vector = system1.Zeeman2.field; reference = "0 0 1";}

}
Run
{
    Task StaticirectYield {type = "Statichs-direct-yields"; logfile = "Dynamic4Spins.log"; datafile = "Dynamic4Spins.dat";
      transitionyields = true; initialstate = Singlet; totaltime = 550.0; timestep = 0.5; propagationmethod = "AutoExpm";
      precision = "single"; yieldcorrections = true;}
}
```

S8.8 14 Nuclear Spin Relaxation (550 ns) - Figure S7

This is the input file used to calculate the spin relaxation effects for the 14 nuclear spin system for the 550 ns MD trajectory.

```
SpinSystem system1
{
    // -------------------------------------------------------
    // Spins
    // -------------------------------------------------------

    // Electrons
    Spin E1 {type = electron; spin = 1/2; tensor = isotropic(2.0023);}
    Spin E2 {type = electron; spin = 1/2; tensor = isotropic(2.0023);}

   // Nitrogens
    Spin FADN5 {type = nucleus; spin = 1; tensor = isotropic(1);}
    Spin FADN10 {type = nucleus; spin = 1; tensor = isotropic(1);}

    Spin TRPNE1 {type = nucleus; spin = 1; tensor = isotropic(1);}

    // Hydrogens
    Spin FADH6 {type = nucleus; spin = 1/2; tensor = isotropic(1);}
    Spin FADHBeta1 {type = nucleus; spin = 1/2; tensor = isotropic(1);}
    Spin FADH81 {type = nucleus; spin = 1/2; tensor = isotropic(1);}
    Spin FADH82 {type = nucleus; spin = 1/2; tensor = isotropic(1);}
    Spin FADH83 {type = nucleus; spin = 1/2; tensor = isotropic(1);}

   Spin TRPHB1 {type = nucleus; spin = 1/2; tensor = isotropic(1);}
    Spin TRPHB2 {type = nucleus; spin = 1/2; tensor = isotropic(1);}
    Spin TRPHD1 {type = nucleus; spin = 1/2; tensor = isotropic(1);}
    Spin TRPHE1 {type = nucleus; spin = 1/2; tensor = isotropic(1);}
    Spin TRPHE3 {type = nucleus; spin = 1/2; tensor = isotropic(1);}
    Spin TRPHH2 {type = nucleus; spin = 1/2; tensor = isotropic(1);}
    Spin TRPNE1 {type = nucleus; spin = 1; tensor = isotropic(1);}

    // ------------------------
    // Zeeman interaction
    // ------------------------

    Interaction Zeeman1 {prefactor=1e-3; type=zeeman; field="0.0 0.0 0.05"; spins=E1;}
    Interaction Zeeman2 {prefactor=1e-3; type=zeeman; field="0.0 0.0 0.05"; spins=E2;}

    // ------------------------
    // Hyperfine interactions
    // ------------------------

    Interaction FADHYP1 {prefactor = 1.0; type = hyperfine; group1 = E1; group2 = FADN5; tensor =
      trajectory("motions_N5.mst"); trajectory = "motions_N5.mst";}
    Interaction FADHYP2 {prefactor = 1.0; type = hyperfine; group1 = E1; group2 = FADN10; tensor =
      trajectory("motions_N10.mst"); trajectory = "motions_N10.mst";}
    Interaction FADHYP3 {prefactor = 1.0; type = hyperfine; group1 = E1; group2 = FADH6; tensor =
      trajectory("motions_H6.mst"); trajectory = "motions_H6.mst";}
    Interaction FADHYP4 {prefactor = 1.0; type = hyperfine; group1 = E1; group2 = FADHBeta1; tensor =
      trajectory("motions_H1beta.mst"); trajectory = "motions_H1beta.mst";}
    Interaction FADHYP5 {prefactor = 1.0; type = hyperfine; group1 = E1; group2 = FADH81; tensor =
      trajectory("motions_H81.mst"); trajectory = "motions_H81.mst";}
    Interaction FADHYP6 {prefactor = 1.0; type = hyperfine; group1 = E1; group2 = FADH82; tensor =
      trajectory("motions_H82.mst"); trajectory = "motions_H82.mst";}
```

```
    Interaction FADHYP7 {prefactor = 1.0; type = hyperfine; group1 = E1; group2 = FADH83; tensor =
      trajectory("motions_H83.mst"); trajectory = "motions_H83.mst";}

    Interaction TRPHYP1 {prefactor = 1.0; type = hyperfine; group1 = E2; group2 = TRPHB1; tensor =
      trajectory("motions_HB1_trp.mst"); trajectory = "motions_HB1_trp.mst";}
    Interaction TRPHYP2 {prefactor = 1.0; type = hyperfine; group1 = E2; group2 = TRPHB2; tensor =
      trajectory("motions_HB2_trp.mst"); trajectory = "motions_HB2_trp.mst";}
    Interaction TRPHYP3 {prefactor = 1.0; type = hyperfine; group1 = E2; group2 = TRPHD1; tensor =
      trajectory("motions_HD1_trp.mst"); trajectory = "motions_HD1_trp.mst";}
    Interaction TRPHYP4 {prefactor = 1.0; type = hyperfine; group1 = E2; group2 = TRPHE1; tensor =
      trajectory("motions_HE1_trp.mst"); trajectory = "motions_HE1_trp.mst";}
    Interaction TRPHYP5 {prefactor = 1.0; type = hyperfine; group1 = E2; group2 = TRPHE3; tensor =
      trajectory("motions_HE3_trp.mst"); trajectory = "motions_HE3_trp.mst";}
    Interaction TRPHYP6 {prefactor = 1.0; type = hyperfine; group1 = E2; group2 = TRPHH2; tensor =
      trajectory("motions_HH2_trp.mst"); trajectory = "motions_HH2_trp.mst";}
    Interaction TRPHYP7 {prefactor = 1.0; type = hyperfine; group1 = E2; group2 = TRPNE1; tensor =
      trajectory("motions_NE1_trp.mst"); trajectory = "motions_NE1_trp.mst";}

    // -------------------------
    // Spin States
    // -------------------------------------------------------

    State Singlet {spins(E1,E2) = |1/2,-1/2> - |-1/2,1/2>;}
    State T0 {spins(E1,E2) = |1/2,-1/2> + |-1/2,1/2>;}
    State Tp {spin(E2) = |1/2>; spin(E1) = |1/2>;}
    State Tm {spin(E2) = |-1/2>; spin(E1) = |-1/2>;}
    State Identity {}

    // -------------------------------------------------------
    // Transitions
    // -------------------------------------------------------

    Transition Product1 {type = sink; source = Singlet; rate = 1.0e-3;} // in 1/ns
    Transition Product2 {type = sink; source = T0; rate = 1.0e-3;}
    Transition Product3 {type = sink; source = Tp; rate = 1.0e-3;}
    Transition Product4 {type = sink; source = Tm; rate = 1.0e-3;}

}
// -------------------------------------------------------------
Settings
{
    Settings general {steps = 51; notifications = details;}

    // -------------------------------------------------------
    // Actions
    // -------------------------------------------------------

    Action scan1 {type = rotatevector; vector = system1.Zeeman1.field; axis = "0 1 0"; value = 3.6;}
    Action scan2 {type = rotatevector; vector = system1.Zeeman2.field; axis = "0 1 0"; value = 3.6;}

    // -------------------------------------------------------
    // Outputs objects
    // -------------------------------------------------------

    Output orientation1 {type = vectorangle; vector = system1.Zeeman1.field; reference = "0 0 1";}
    Output orientation2 {type = vectorangle; vector = system1.Zeeman2.field; reference = "0 0 1";}
}
Run
{
    Task DynamicStochYield {type = "dynamichs-stoch-yields"; logfile = "Dynamic14Spins.log"; datafile = "Dynamic14Spins.dat";
      transitionyields = true; initialstate = Singlet; montecarlosamples = 5; samplingmethod = "SUZ"; autoseed = false; seed
      = 1; totaltime = 550.0; timestep = 0.5; propagationmethod = "AutoExpm"; precision = "single"; yieldcorrections = true;}
}
```

This is the input file used to calculate the static case without time-dependent motions.

```
SpinSystem system1
{
// -------------------------------------------------------
// Spins
// -------------------------------------------------------

    // Electrons
    Spin E1 {type = electron; spin = 1/2; tensor = isotropic(2.0023);}
    Spin E2 {type = electron; spin = 1/2; tensor = isotropic(2.0023);}
    // Nitrogens
    Spin FADN5 {type = nucleus; spin = 1; tensor = isotropic(1);}
    Spin FADN10 {type = nucleus; spin = 1; tensor = isotropic(1);}
    Spin TRPNE1 {type = nucleus; spin = 1; tensor = isotropic(1);}
    // Hydrogens
    Spin FADH6 {type = nucleus; spin = 1/2; tensor = isotropic(1);}
    Spin FADHBeta1 {type = nucleus; spin = 1/2; tensor = isotropic(1);}
    Spin FADH81 {type = nucleus; spin = 1/2; tensor = isotropic(1);}
    Spin FADH82 {type = nucleus; spin = 1/2; tensor = isotropic(1);}
    Spin FADH83 {type = nucleus; spin = 1/2 tensor = isotropic(1);}
    Spin TRPHB1 {type = nucleus; spin = 1/2; tensor = isotropic(1);}
    Spin TRPHB2 {type = nucleus; spin = 1/2; tensor = isotropic(1);}
    Spin TRPHD1 {type = nucleus; spin = 1/2; tensor = isotropic(1);}
    Spin TRPHE1 {type = nucleus; spin = 1/2; tensor = isotropic(1);}
    Spin TRPHE3 {type = nucleus; spin = 1/2; tensor = isotropic(1);}
    Spin TRPHH2 {type = nucleus; spin = 1/2; tensor = isotropic(1);}
    Spin TRPNE1 {type = nucleus; spin = 1; tensor = isotropic(1);}

    // -------------------------
    // Zeeman interaction
    // -------------------------

    Interaction Zeeman1 {prefactor=1e-3; type=zeeman; field="0.0 0.0 0.05"; spins=E1;}
    Interaction Zeeman2 {prefactor=1e-3; type=zeeman; field="0.0 0.0 0.05"; spins=E2;}

    // -------------------------
    // Hyperfine interactions
    // -------------------------

    Interaction FADHYP1 {prefactor = 1e-3; type = hyperfine; group1 = E1; group2 = FADN5; tensor = matrix("-0.09359, 0.00000,
      0.00000; 0.00000, -0.09359, 0.00000; 0.00000, 0.00000, 1.88074");}
    Interaction FADHYP2 {prefactor = 1e-3; type = hyperfine; group1 = E1; group2 = FADN10; tensor = matrix("0.022096,
      -0.011987, -0.081181; -0.011987, 0.016364, 0.047916; -0.081181, 0.047916, 0.684963");}
    Interaction FADHYP3 {prefactor = 1e-3; type = hyperfine; group1 = E1; group2 = FADH6; tensor = matrix("-0.251908,
      -0.123828, 0.001443; -0.123828, -0.408730, -0.000380; 0.001443, -0.000380, -0.408936");}
    Interaction FADHYP4 {prefactor = 1e-3; type = hyperfine; group1 = E1; group2 = FADHBeta1; tensor = matrix("0.121195,
      -0.057543, 0.030752; -0.057543, 0.156364, -0.039068; 0.030752, -0.039068, 0.082324");}
    Interaction FADHYP5 {prefactor = 1e-3; type = hyperfine; group1 = E1; group2 = FADH81; tensor = matrix("0.59178,
      0.03024, 0.01102; 0.03024, 0.66945, 0.02582; 0.01102, 0.02582, 0.58894");}
    Interaction FADHYP6 {prefactor = 1e-3; type = hyperfine; group1 = E1; group2 = FADH82; tensor = matrix("-0.00471,
      -0.02388, 0.00000; -0.02388, 0.07270, 0.00167; 0.0000, 0.00167, -0.00784");}
    Interaction FADHYP7 {prefactor = 1e-3; type = hyperfine; group1 = E1; group2 = FADH83; tensor = matrix("0.61530, 0.02921,
      -0.00917; 0.02921, 0.69678, -0.02473; -0.00917, -0.02473, 0.61306");}
```

```

    Interaction TRPHYP1 {prefactor = 1e-3; type = hyperfine; group1 = E2; group2 = TRPHB1; tensor = matrix("0.27841,
      -0.11523, 0.03489; -0.11523, 0.25048, -0.01437; 0.03489, -0.01437, 0.15359");}
    Interaction TRPHYP2 {prefactor = 1e-3; type = hyperfine; group1 = E2; group2 = TRPHB2; tensor = matrix("1.28050,
      -0.02818, 0.11121; -0.02818, 1.20832, 0.00315; 0.11121, 0.00315, 1.32975");}
    Interaction TRPHYP3 {prefactor = 1e-3; type = hyperfine; group1 = E2; group2 = TRPHD1; tensor = matrix("-0.84706,
      0.04893, -0.09419; 0.04893, -0.29433, 0.24691; -0.09419, 0.24691, -0.54792");}
    Interaction TRPHYP4 {prefactor = 1e-3; type = hyperfine; group1 = E2; group2 = TRPHE1; tensor = matrix("-0.3039, 0.20685,
      0.27805; 0.20685, -0.68879, 0.04186; 0.27805, 0.04186, -0.36458");}
    Interaction TRPHYP5 {prefactor = 1e-3; type = hyperfine; group1 = E2; group2 = TRPHE3; tensor = matrix("-0.37054,
      0.06995, 0.18925; 0.06995, -0.76615, -0.06602; 0.18925, -0.06602, -0.51155");}
    Interaction TRPHYP6 {prefactor = 1e-3; rtype = hyperfine; group1 = E2; group2 = TRPHH2; tensor = matrix("-0.47286,
      -0.19165, -0.12872; -0.19165, -0.27373, -0.00185; -0.12872, -0.00185, -0.53654");}
    Interaction TRPHYP7 {prefactor = 1e-3; type = hyperfine; group1 = E2; group2 = TRPNE1; tensor = matrix("0.08309, 0.16473,
      -0.26682; 0.16473, 0.07569, -0.27837; -0.26682, -0.27837, 0.36617");}

    // -------------------------
    // Spin States
    // -------------------------------------------------------

    State Singlet {spins(E1,E2) = |1/2,-1/2> - |-1/2,1/2>;}
    State T0 {spins(E1,E2) = |1/2,-1/2> + |-1/2,1/2>;}
    State Tp {spin(E2) = |1/2>; spin(E1) = |1/2>;}
    State Tm {spin(E2) = |-1/2>; spin(E1) = |-1/2>;}
    State Identity {}

    // -------------------------------------------------------
    // Transitions
    // -------------------------------------------------------

    Transition Product1 {type = sink; source = Singlet; rate = 1.0e-3;} // in 1/ns
    Transition Product2 {type = sink; source = T0; rate = 1.0e-3;}
    Transition Product3 {type = sink; source = Tp; rate = 1.0e-3;}
    Transition Product4 {type = sink; source = Tm; rate = 1.0e-3;}
}
// -------------------------------------------------------------
Settings
{
    Settings general {steps = 51; notifications = details;}

    // -------------------------------------------------------
    // Actions
    // -------------------------------------------------------

    Action scan1 {type = rotatevector; vector = system1.Zeeman1.field; axis = "0 1 0"; value = 3.6;}
    Action scan2 {type = rotatevector; vector = system1.Zeeman2.field; axis = "0 1 0"; value = 3.6;}

    // -------------------------------------------------------
    // Outputs objects
    // -------------------------------------------------------

    Output orientation1 {type = vectorangle; vector = system1.Zeeman1.field; reference = "0 0 1";}
    Output orientation2 {type = vectorangle; vector = system1.Zeeman2.field; reference = "0 0 1";}
}
Run
{
    Task StaticHSStochYield {type = "statichs-stoch-yields"; logfile = "Static14Spins.log"; datafile = "Static14Spins.dat";
      transitionyields = true; initialstate = Singlet; montecarlosamples = 5; samplingmethod = "SUZ"; autoseed = false; seed
      = 1; epsilon = 0.5; timestep = 4; propagationmethod = "AutoExpm"; precision = "single"; yieldcorrections = true;}
}
```

S8.9 14 Nuclear Spin Relaxation for Full Dynamic Picture (0.953 μs) - Figure 6

The following input was used to calculated the static scenario of the 0.953 ns MD trajectory extracted hyperine and dipolar couplings using a 1 mT magnetic field.

```
SpinSystem system1
{
  // Electrons
  Spin E1 {type = electron;spin = 1/2;tensor = isotropic(2.0023);}
  Spin E2 {type = electron;spin = 1/2;tensor = isotropic(2.0023);}

  // Nitrogens
  Spin FADN5 {type = nucleus;spin = 1; tensor = isotropic(1);}
          Spin FADN10 {type = nucleus;spin = 1; tensor = isotropic(1);}
  // Hydrogens
  Spin FADH6 {type = nucleus;spin = 1/2; tensor = isotropic(1);}
  Spin FADHBeta1 {type = nucleus;spin = 1/2; tensor = isotropic(1);}
  Spin FADH81 {type = nucleus;spin = 1/2; tensor = isotropic(1);}
          Spin FADH82 {type = nucleus;spin = 1/2; tensor = isotropic(1);}
         Spin FADH83 {type = nucleus;spin = 1/2; tensor = isotropic(1);}

  Spin TRPN1 {type = nucleus;spin = 1; tensor = isotropic(1);}
  Spin TRPH1 {type = nucleus;spin = 1/2; tensor = isotropic(1);}
          Spin TRPH2 {type = nucleus;spin = 1/2; tensor = isotropic(1);}
  Spin TRPH4 {type = nucleus;spin = 1/2; tensor = isotropic(1);}
  Spin TRPH6 {type = nucleus;spin = 1/2; tensor = isotropic(1);}
  Spin TRPH7 {type = nucleus;spin = 1/2; tensor = isotropic(1);}
  Spin TRPHbeta1 {type = nucleus;spin = 1/2; tensor = isotropic(1);}
  // -------------------------
  // Zeeman interaction
  // -------------------------

  Interaction Zeeman1 {prefactor= 1e-3;type = zeeman;field = "0.0 0.0 1.0"; spins = E1;}
  Interaction Zeeman2 {prefactor= 1e-3;type = zeeman;field = "0.0 0.0 1.0"; spins = E2;}

  // -------------------------
  // Hyperfine interactions
  // -------------------------

  Interaction FADHYP1 { prefactor = 1.0e-3;  type = hyperfine; group1 = E1; group2 = FADN5; tensor = matrix("0.52007444,
      -0.17396341,  0.77386319; -0.17396341, 0.04412077, -0.24317156; 0.77386319, -0.24317156, 1.14359574");}
```

```
  Interaction FADHYP2 { prefactor = 1.0e-3; type = hyperfine; group1 = E1; group2 = FADN10; tensor = matrix("0.18298346,
     -0.05277072,  0.25433445; -0.05277072,  0.02190145, -0.07020126; 0.25433445, -0.07020126,  0.39242808");}
  Interaction FADHYP3 { prefactor = 1.0e-3; type = hyperfine; group1 = E1; group2 = FADH6; tensor = matrix("-0.2436865,
     -0.04785683, -0.10072132; -0.04785683, -0.44075707, 0.01618169; -0.10072132, 0.01618169, -0.3433513");}
  Interaction FADHYP4 { prefactor = 1.0e-3; type = hyperfine; group1 = E1; group2 = FADHBeta1; tensor = matrix("0.14096681,
     -0.03056939, -0.05442738;-0.03056939,  0.13897252,  0.04320126;-0.05442738,  0.04320126,  0.16649513");}
  Interaction FADHYP5 {prefactor=1.0e-3; type = hyperfine; group1 = E1; group2 = FADH81; tensor = matrix("0.39422546,
     -0.00411337, -0.00150647; -0.00411337, 0.46730559, 0.01639295; -0.00150647, 0.01639295, 0.39743516");}
        Interaction FADHYP6 { prefactor = 1.0e-3; type = hyperfine; group1 = E1; group2 = FADH82; tensor =
     matrix("0.39264967, -0.00429489, -0.00158974; -0.00429489,  0.46542371, 0.01649609; -0.00158974, 0.01649609,
     0.39573982");}
        Interaction FADHYP7 { prefactor = 1.0e-3; type = hyperfine; group1 = E1; group2 = FADH83; tensor =
     matrix("0.39603212, -0.00424982, -0.00160015; -0.00424982,  0.46886296,  0.01667159; -0.00160015,  0.01667159,
     0.39929243");}

  Interaction TRPHYP1 { prefactor = 1.0e-3; type = hyperfine; group1 = E2; group2 = TRPN1; tensor = matrix("0.52785038,
     0.35898356, 0.29370566; 0.35898356, 0.29105545, 0.21422574; 0.29370566, 0.21422574, 0.22693535");}
        Interaction TRPHYP2 { prefactor = 1.0e-3; type = hyperfine; group1 = E2; group2 = TRPH1; tensor =
     matrix("-0.66464468, 0.18025424, -0.01356128; 0.18025424, -0.5074232, -0.34080836; -0.01356128, -0.34080836,
     -0.04602107"7);}
        Interaction TRPHYP3 { prefactor = 1.0e-3; type = hyperfine; group1 = E2; group2 = TRPH2; tensor =
     matrix("-0.38499246, -0.20772771,  0.09630866; -0.20772771, -0.16899912, -0.03159051; 0.09630866, -0.03159051,
     -0.58958077");}
  Interaction TRPHYP4 { prefactor = 1.0e-3; type = hyperfine; group1 = E2; group2 = TRPH4; tensor = matrix("-0.65093005,
     0.16700591, -0.11514255; 0.16700591, -0.70875244, -0.15378603; -0.11514255, -0.15378603, -0.34527729");}
        Interaction TRPHYP5 { prefactor = 1.0e-3; type = hyperfine; group1 = E2; group2 = TRPH6; tensor =
     matrix("-0.2019179,  -0.13717574, -0.04670033; -0.13717574, -0.24083608,  0.11927501; -0.04670033,  0.11927501,
     -0.35520921");}
        Interaction TRPHYP6 { prefactor = 1.0e-3; type = hyperfine; group1 = E2; group2 = TRPH7; tensor =
     matrix("-0.42155253,  0.10419954, -0.09128151; 0.10419954, -0.42748508, -0.12276762; -0.09128151, -0.12276762,
     -0.19691742");}
        Interaction TRPHYP7 { prefactor = 1.0e-3; type = hyperfine; group1 = E2; group2 = TRPHbeta1; tensor =
     matrix("0.22845924, -0.05356532, -0.08962462; -0.05356532, 0.19649593, 0.04482989; -0.08962462, 0.04482989,
     0.26994276");}

  // -------------------------
  // Dipolar interactions
  // -------------------------

  Interaction Dipolar { prefactor = 2.0023e-3; IgnoreTensors = true; type = doublespin; group1 = E1; group2 = E2;
     tensor=matrix("0.44628577, 0.38647182, 0.12319905; 0.38647182, -0.13644849, 0.0615624; 0.12319905, 0.0615624,
     -0.30983728");}
      // We ignore the tensor variable from the Spin objects  and add 2.0023 to the prefactor to only account for one g-value
     for conversion from Tesla into rad/ns (MolSpin multiplies each interaction by a CommonPrefactor (Bohr Magneton/hbar) if
     not set
              to false

  // ---------------------------------------------------------
  // Spin States
  // ----------------------------------------------------------

           State Singlet {spins(E1,E2) = |1/2,-1/2> - |-1/2,1/2>;}
  State T0 {spins(E1,E2) = |1/2,-1/2> + |-1/2,1/2>;}
  State Tp {spin(E2) = |1/2>; spin(E1) = |1/2>;}
  State Tm {spin(E2) = |-1/2>; spin(E1) = |-1/2>;}
  State Identity {}

        // ---------------------------------------------------------
        // Transitions
        // ---------------------------------------------------------

  Transition Product1 {type = sink; source = Singlet; rate=1.0e-3;} // in 1/ns
  Transition Product2 {type = sink; source = T0; rate=1.0e-3;}
  Transition Product3 {type = sink; source = Tp; rate=1.0e-3;}
  Transition Product4 {type = sink; source = Tm; rate=1.0e-3;}

}
// -------------------------------------------------------------
Settings { Settings general {steps = 1; notifications = details;} }
Run
{
  Task StaticHS_Stoch_Yield
        {
    type = "statichs-stoch-yields";
    logfile = "Dynamic14Spins.log";
                  datafile = "Dynamic14Spins.dat";
    transitionyields = true;
    initialstate = Singlet;
    montecarlosamples = 5;
                  samplingmethod = "SUZ";
                  autoseed = false;
                  seed = 1;
    epsilon = 0.1;
    timestep=0.05;
    propagationmethod = "AutoExpm";
    precision = "single";
    yieldcorrections = true;
        }
}
```

The following input was used to calculated the full dynamics scenario of the 0.953 ns MD trajectory extracted hyperine and dipolar couplings using a 1 mT magnetic field.

```
SpinSystem system1
{
        // Electrons
        Spin E1 {type = electron;spin = 1/2; tensor = isotropic(2.0023);}
        Spin E2 {type = electron;spin = 1/2; tensor = isotropic(2.0023);}

        // Nitrogens (FAD)
        Spin FADN5 {type = nucleus;spin = 1; tensor = isotropic(1.0);}
        Spin FADN10 {type = nucleus;spin = 1; tensor = isotropic(1.0);}

        // Hydrogens (FAD)
        Spin FADH6 {type = nucleus;spin = 1/2; tensor = isotropic(1.0);}
        Spin FADHBeta1 {type = nucleus;spin = 1/2; tensor = isotropic(1.0);}
        Spin FADH81 {type = nucleus;spin = 1/2; tensor = isotropic(1.0);}
        Spin FADH82 {type = nucleus;spin = 1/2; tensor = isotropic(1.0);}
        Spin FADH83 {type = nucleus;spin = 1/2; tensor = isotropic(1.0);}

        // Nitrogens (TRP)
        Spin TRPN1 {type = nucleus;spin = 1; tensor = isotropic(1.0);}

        // Hydrogens (TRP)
        Spin TRPH1 {type = nucleus;spin = 1/2; tensor = isotropic(1.0);}
```

```
        Spin TRPH2 {type = nucleus;spin = 1/2; tensor = isotropic(1.0);}
        Spin TRPH4 {type = nucleus;spin = 1/2; tensor = isotropic(1.0);}
        Spin TRPH6 {type = nucleus;spin = 1/2; tensor = isotropic(1.0);}
        Spin TRPH7 {type = nucleus;spin = 1/2; tensor = isotropic(1.0);}
        Spin TRPHbeta1 {type = nucleus;spin = 1/2; tensor = isotropic(1.0);}

        // -------------------------
        // Zeeman interaction
        // -------------------------

        Interaction Zeeman1 {prefactor= 1e-3;type = zeeman;field = "0.000 0.000 1.000";spins = E1;}
        Interaction Zeeman2 {prefactor= 1e-3;type = zeeman;field = "0.000 0.000 1.000";spins = E2;}

        // -------------------------
        // Hyperfine interactions
        // -------------------------

        Interaction FADHYP1 {type = hyperfine; group1 = E1; group2 = FADN5;
                tensor = trajectory("motions_FAD_N5_dt_50ps.mst"); trajectory = "motions_FAD_N5_dt_50ps.mst";}

        Interaction FADHYP2 {type = hyperfine; group1 = E1; group2 = FADN10;
                tensor = trajectory("motions_FAD_N10_dt_50ps.mst"); trajectory = "motions_FAD_N10_dt_50ps.mst";}

        Interaction FADHYP3 {type = hyperfine; group1 = E1; group2 = FADH6;
                tensor = trajectory("motions_FAD_H6_dt_50ps.mst"); trajectory = "motions_FAD_H6_dt_50ps.mst";}

        Interaction FADHYP4 {type = hyperfine; group1 = E1; group2 = FADHBeta1;
                tensor = trajectory("motions_FAD_Hbeta1_dt_50ps.mst"); trajectory = "motions_FAD_Hbeta1_dt_50ps.mst";}

        Interaction FADHYP5 {type = hyperfine; group1 = E1; group2 = FADH81;
                tensor = trajectory("motions_FAD_H81_dt_50ps.mst"); trajectory = "motions_FAD_H81_dt_50ps.mst";}

        Interaction FADHYP6 {type = hyperfine; group1 = E1; group2 = FADH82;
                tensor = trajectory("motions_FAD_H82_dt_50ps.mst"); trajectory = "motions_FAD_H82_dt_50ps.mst";}

        Interaction FADHYP7 {type = hyperfine; group1 = E1; group2 = FADH83;
                tensor = trajectory("motions_FAD_H83_dt_50ps.mst"); trajectory = "motions_FAD_H83_dt_50ps.mst";}

        Interaction TRPHYP1 {type = hyperfine; group1 = E2; group2 = TRPN1;
                tensor = trajectory("motions_TRP_N1_dt_50ps.mst"); trajectory = "motions_TRP_N1_dt_50ps.mst";}

        Interaction TRPHYP2 {type = hyperfine; group1 = E2; group2 = TRPH1;
                tensor = trajectory("motions_TRP_H1_dt_50ps.mst"); trajectory = "motions_TRP_H1_dt_50ps.mst"; }

        Interaction TRPHYP3 {type = hyperfine; group1 = E2; group2 = TRPH2;
                tensor = trajectory("motions_TRP_H2_dt_50ps.mst"); trajectory = "motions_TRP_H2_dt_50ps.mst";}

        Interaction TRPHYP4 {type = hyperfine; group1 = E2; group2 = TRPH4;
                tensor = trajectory("motions_TRP_H4_dt_50ps.mst"); trajectory = "motions_TRP_H4_dt_50ps.mst";}

        Interaction TRPHYP5 {type = hyperfine; group1 = E2; group2 = TRPH6;
                tensor = trajectory("motions_TRP_H6_dt_50ps.mst"); trajectory = "motions_TRP_H6_dt_50ps.mst";}

        Interaction TRPHYP6 {type = hyperfine; group1 = E2; group2 = TRPH7;
                tensor = trajectory("motions_TRP_H7_dt_50ps.mst"); trajectory = "motions_TRP_H7_dt_50ps.mst";}

        Interaction TRPHYP7 {type = hyperfine; group1 = E2; group2 = TRPHbeta1;
                tensor = trajectory("motions_TRP_Hbeta1_dt_50ps.mst"); trajectory = "motions_TRP_Hbeta1_dt_50ps.mst";}


        // -------------------------
        // Dipolar interactions
        // -------------------------
        Interaction Dipolar { prefactor = 2.0023; IgnoreTensors = true;type = doublespin; group1 = E1; group2 = E2;
                tensor = trajectory("dipolar_dt_50ps.mst"); trajectory = "dipolar_dt_50ps.mst";}

        // -------------------------
        // Spin States
        // ---------------------------------------------------------

        State Singlet {spins(E1,E2) = |1/2,-1/2> - |-1/2,1/2>;}

        State T0 {spins(E1,E2) = |1/2,-1/2> + |-1/2,1/2>;}

        State Tp {spin(E2) = |1/2>; spin(E1) = |1/2>;}

        State Tm {spin(E2) = |-1/2>; spin(E1) = |-1/2>;}

        State Identity {}

        // ---------------------------------------------------------
        // Transitions
        // ---------------------------------------------------------

        Transition Product1 {type = sink; source = Singlet; rate=1.0e-3;}

        Transition Product2 {type = sink; source = T0; rate=1.0e-3;}

        Transition Product3 {type = sink; source = Tp; rate=1.0e-3;}

        Transition Product4 {type = sink; source = Tm; rate=1.0e-3;}

}
// -------------------------------------------------------------
Settings {

        Settings general {steps = 1; notifications = details;}

//        Action scan1 {type = rotatevector; vector = system1.Zeeman1.field ; axis = "0 1 0"; value = 3.6;}
//        Action scan2 {type = rotatevector; vector = system1.Zeeman2.field; axis = "0 1 0"; value = 3.6;}

}
Run
{
        Task StaticHS_Stoch_Yield
        {
                type = "dynamichs-stoch-yields";
                logfile = "14SpinsCorrectedUnitsFDynamic_0.000.log";
                datafile = "14SpinsCorrectedUnitsFDynamic_0.000.dat";
                transitionyields = true;
                initialstate = Singlet;
                montecarlosamples = 5;
                samplingmethod = "SUZ";
                autoseed = false;
                seed = 1;
                totaltime = 953.0;
                timestep=0.05;
                propagationmethod = "AutoExpm";
                precision = "single";
                yieldcorrections = true;
        }
}
```

References

(1) Brocklehurst, B.; McLauchlan, K. A. *Int. J. Radiat. Biol.* **1996**, *69*, 3–24.

(2) Hiscock, H. G.; Worster, S.; Kattnig, D. R.; Steers, C.; Jin, Y.; Manolopoulos, D. E.; Mouritsen, H.; Hore, P. J. *Proc. Natl. Acad. Sci.* **2016**, *113*, 4634–4639.

(3) Fay, T. P.; Lindoy, L. P.; Manolopoulos, D. E.; Hore, P. J. *Faraday Discussions* **2020**, *221*, 77–91.

(4) Smith, L. D.; Chowdhury, F. T.; Peasgood, I.; Dawkins, N.; Kattnig, D. R. *J. Phys. Chem. Lett.* **2022**, *13*, 10500–10506.

(5) Wong, S. Y.; Benjamin, P.; Hore, P. J. *Phys. Chem. Chem. Phys.* **2023**, *25*, 975–982.

(6) Gerhards, L.; Nielsen, C.; Kattnig, D. R.; Hore, P. J.; Solov'yov, I. A. *J. Comput. Chem.* **2023**, *44*, 1704–1714.

(7) Fay, T. P.; Lindoy, L. P.; Manolopoulos, D. E. *J. Chem. Phys.* **2019**, *151*, 154117.

(8) Weiße, A.; Wellein, G.; Alvermann, A.; Fehske, H. *Reviews of modern physics* **2006**, *78*, 275.

(9) Fay, T. P.; Lindoy, L. P.; Manolopoulos, D. E. *J. Chem. Phys.* **2021**, *154*, 084121.

(10) Al-Mohy, A. H.; Higham, N. J. *SIAM J. Sci. Comput.* **2011**, *33*, 488–511.

(11) Leforestier, C.; Bisseling, R.; Cerjan, C.; Feit, M.; Friesner, R.; Guldberg, A.; Hammerich, A.; Jolicard, G.; Karrlein, W.; Meyer, H.-D., et al. *Journal of Computational Physics* **1991**, *94*, 59–80.

(12) Tal-Ezer, H.; Kosloff, R.; Schaefer, I. *Journal of Scientific Computing* **2012**, *53*, 211–221.

(13) Neuhauser, D.; Baer, M.; Judson, R. S.; Kouri, D. J. *Computer physics communications* **1991**, *63*, 460–481.

(14) Pollard, W. T.; Friesner, R. A. *The Journal of chemical physics* **1994**, *100*, 5054–5065.

(15) **http://www.molspin.eu**.

(16) Kattnig, D. R.; Solov'yov, I. A.; Hore, P. J. *Phys. Chem. Chem. Phys.* **2016**, *18*, 12443–12456.

(17) Kattnig, D. R. *J. Phys. Chem. B* **2017**, *121*, 10215–10227.

(18) Breuer, H.-P.; Petruccione, F., *The theory of open quantum systems*; Oxford University Press: 2002.

(19) Grüning, G.; Wong, S. Y.; Gerhards, L.; Schuhmann, F.; Kattnig, D. R.; Hore, P. J.; Solov'yov, I. A. *J. Am. Chem. Soc.* **2022**, *144*, PMID: 36459632, 22902–22914.

(20) Frisch, M. J. et al. **2013**, Gaussian Inc. Wallingford CT 2009.

(21) Timmel, C.; Till, U.; Brocklehurst, B.; Mclauchlan, K.; Hore, P. J. *Mol. Phys.* **1998**, *95*, 71–89.

(22) Fay, T. P.; Lewis, A. M.; Manolopoulos, D. E. *J. Chem. Phys.* **2017**, *147*, 064107.
